# Toolkit and distance coaching strategies: a mixed methods evaluation of a trial to implement care coordination quality improvement projects in primary care

**DOI:** 10.1186/s12913-021-06850-1

**Published:** 2021-08-14

**Authors:** Lauren S. Penney, Purnima S. Bharath, Isomi Miake-Lye, Mei Leng, Tanya T. Olmos-Ochoa, Erin P. Finley, Neetu Chawla, Jenny M. Barnard, David A. Ganz

**Affiliations:** 1 Elizabeth Dole Center of Excellence for Veteran & Caregiver Research, South Texas Veterans Health Care System, San Antonio, TX USA; 2 Department of Medicine, University of Texas Health San Antonio, San Antonio, TX USA; 3 HSR&D Center for the Study of Healthcare Innovation, Implementation and Policy (CSHIIP), VA Greater Los Angeles Healthcare System, Sepulveda, CA USA; 4 Fielding School of Public Health, University of California at Los Angeles, Los Angeles, CA USA; 5 David Geffen School of Medicine, University of California at Los Angeles, Los Angeles, CA USA; 6 RAND Corporation, Santa Monica, California USA

**Keywords:** Quality improvement, Care coordination, Primary care, External facilitation, Toolkit, Consolidated framework for implementation research

## Abstract

**Background:**

Care coordination tools and toolkits can be challenging to implement. Practice facilitation, an active but expensive strategy, may facilitate toolkit implementation. We evaluated the comparative effectiveness of distance coaching, a form of practice facilitation, for improving the implementation of care coordination quality improvement (QI) projects.

**Methods:**

We conducted a mixed methods evaluation of the Coordination Toolkit and Coaching (CTAC) initiative. Twelve matched US Veterans Health Administration primary care clinics were randomized to receive coaching and an online care coordination toolkit (“coached”; *n* = 6) or access to the toolkit only (“non-coached”; *n* = 6). We did interviews at six, 12, and 18 months. For coached sites, we‘ly collected site visit fieldnotes, prospective coach logs, retrospective coach team debriefs, and project reports. We employed matrix analysis using constructs from the Consolidated Framework for Implementation Research and a taxonomy of outcomes. We assessed each site’s project(s) using an adapted Complexity Assessment Tool for Systematic Reviews.

**Results:**

Eleven sites implemented a local CTAC project. Eight sites (5 coached, 3 non-coached) used at least one tool from the toolkit. Coached sites implemented significantly more complex projects than non-coached sites (11.5 vs 7.5, 95% confidence interval 1.75–6.25, *p* < 0.001); engaged in more formal implementation processes (planning, engaging, reflecting and evaluating); and generally had larger, more multidisciplinary QI teams. Regardless of coaching status, sites focused on internal organizational improvement and low-intensity educational projects rather than the full suite of care coordination tools. At 12 months, half the coached and non-coached sites had clinic-wide project implementation; the remaining coached sites had implemented most of their project(s), while the remaining non-coached sites had either not implemented anything or conducted limited pilots. At 18 months, coached sites reported ongoing effort to monitor, adapt, and spread their CTAC projects, while non-coached sites did not report much continuing work. Coached sites accrued benefits like improved clinic relationships and team QI skill building that non-coached sites did not describe.

**Conclusions:**

Coaching had a positive influence on QI skills of (and relationships among) coached sites’ team members, and the scope and rigor of projects. However, a 12-month project period was potentially too short to ensure full project implementation or to address cross-setting or patient-partnered initiatives.

**Trial registration:**

NCT03063294.

**Supplementary Information:**

The online version contains supplementary material available at 10.1186/s12913-021-06850-1.

## Background

Care coordination in health care is “the deliberate organization of patient care activities between two or more participants (including the patient) involved in a patient’s care to facilitate the appropriate delivery of health care services.” [1] Coordination includes engaging staff and resources, and exchanging key information among responsible parties to properly deliver care [2]. Care coordination bridges gaps along patients’ care pathways [1], and may involve a range of tasks from simple (e.g., notifying a patient of lab results) to complex (e.g., planning comprehensive cancer care with the patient, their family, and multiple health care providers).

Significant gaps in care coordination exist in healthcare systems. In the USA, poor care coordination is associated with suboptimal outcomes [3], patient and family dissatisfaction [4–6], and financial waste [7]. Even in systems that are more clinically integrated (e.g., by sharing the same electronic health record), such as the US Veterans Health Administration (VA), care coordination remains a challenge [8–12]. Improving care coordination is a priority area for health system improvement [13, 14].

Effective tools [2] and multiple frameworks exist for improving care coordination [15], but are challenging to implement. Use of implementation strategies, such as evidence-based toolkits and practice facilitation, may help [16]. Toolkits facilitate action by providing information, resources, and tools to bring practice in alignment with evidence-based recommendations or standards [17]. While toolkits show promise for facilitating translation into practice [18], toolkits have variable perceived utility and uptake [19], and there is little evidence regarding their effectiveness on knowledge, behaviors, and patient outcomes [19, 20]. Toolkits on their own, without a more active facilitation strategy that encourages closer engagement with the materials, may not be enough for effective knowledge translation [18]. Combining toolkits with practice facilitation, or assistance from quality improvement (QI) experts, can help implementers to problem-solve challenges, tailor tools, and establish accountability [21] and may be critical for fully implementing toolkits in practice [22, 23]. It has yet to be established, however, whether practice facilitation, as a more intensive and expensive implementation strategy that enables implementers to effectively implement change in complex settings [24], is comparatively a better strategy for implementing effective practices in primary care settings.

In a recently completed study, we sought to determine whether patient experience of care coordination could be improved by combining distance QI coaching (facilitation) and a toolkit, compared to a toolkit-only approach. The primary findings from our evaluation were inconclusive: surveyed patients across both strategies had modest and similar improvements in experience [25]. To interpret these results and inform future work, we also evaluated the comparative effectiveness of the coached and non-coached strategies for improving the implementation of care coordination QI projects.

## Methods

### Aim, design, and study setting

**Fig. 1:**
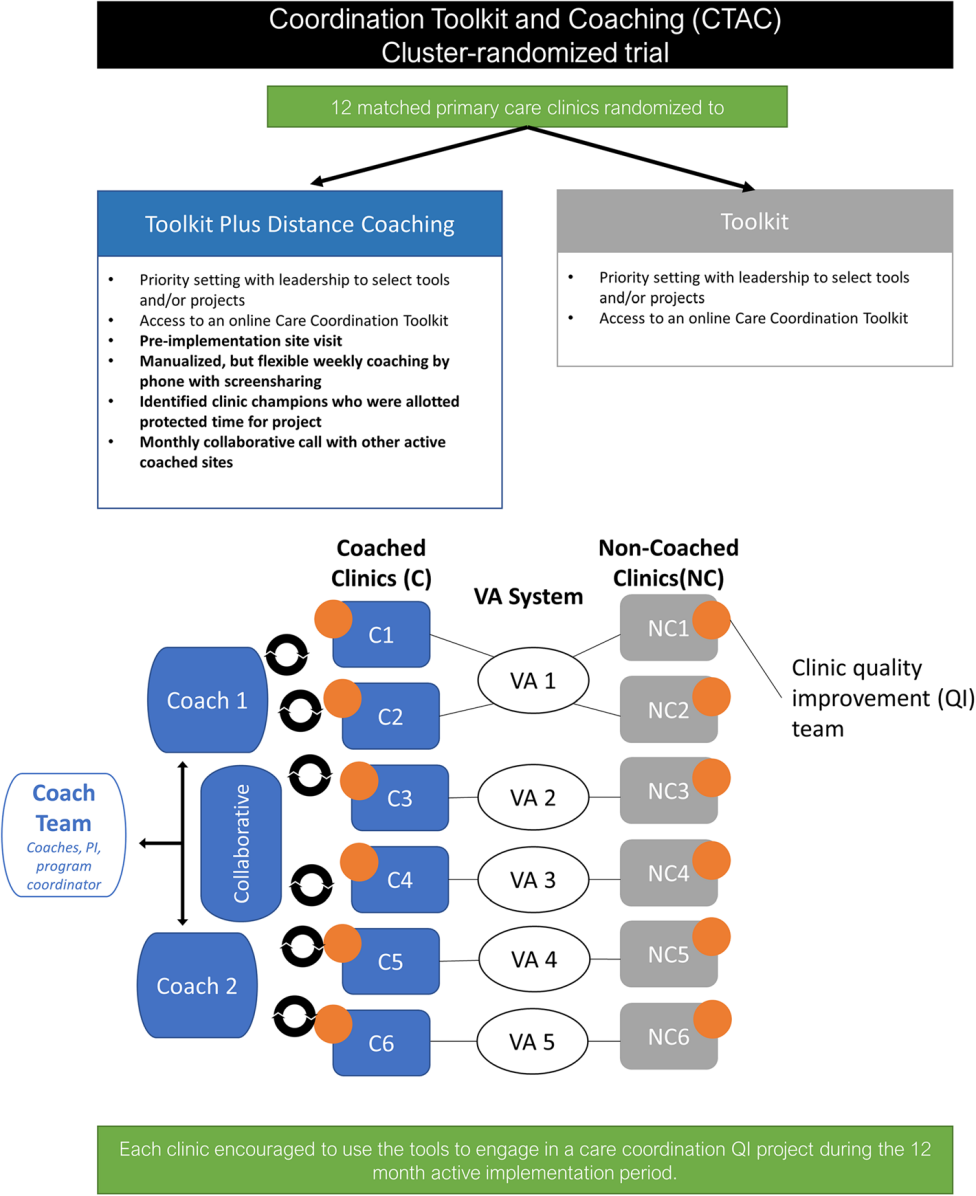
Coordination Toolkit and Coaching (CTAC) design

The Coordination Toolkit and Coaching (CTAC) QI initiative randomized 12 VA primary care clinics (6 matched pairs) to receive either access to an online toolkit (“non-coached”) or distance coaching plus access to an online toolkit (“coached”) (see Fig. 1) [26]. Toolkit development has been described elsewhere [27]. The toolkit included 18 tools and resources for implementation, selected from among 300 candidate tools using a 3-step process that included clinician and patient input (see Additional file 1 for a summary of tools from the toolkit). The VA Office of Primary Care and the Institutional Review Board of the VA Greater Los Angeles Healthcare System determined CTAC to be non-research.

Each CTAC QI coach was the primary coach for three sites and worked 20 h per week on the project. Each coached site received approximately 12 months of access to a coach, including weekly calls with their coach, as needed email and phone support from their coach, and the ability to attend monthly community of practice calls for all currently coached sites. Coaching was piloted at a pilot site and manualized (see Additional file 2). The manual included guidance on supporting local, multidisciplinary QI teams through the process of identifying local care coordination gap(s) and selecting, implementing, and evaluating care coordination tool(s) using Plan-Do-Study-Act cycles. Coaches had flexibility to tailor coaching to site needs. Coaches observed each other’s coaching calls and held debrief meetings after each call; they were also supported by weekly and as-needed consultation with the principal investigator and project coordinator (the four together are hereafter referred to as the “coach team”). CTAC was designed with the thought that sites would select tools from the toolkit that best addressed their care coordination needs as identified in CTAC’s baseline survey of patient experience [26], and then the coach would assist sites in implementing these tools.

### Data collection

Data were collected through semi-structured interviews and supplemented with prospective coach notes, debrief meetings, and site documents. Six and 12 months after the start of active implementation, leadership from the five VA systems, and project champions and frontline staff (who were almost exclusively from the clinics’ CTAC QI teams) from the 12 clinics were invited to participate in semi-structured phone interviews; champions were also invited to participate in interviews at 18 months. Participants were purposively sampled based on engagement in pre-implementation activities (e.g., provided leadership approvals, were part of agenda-setting at the clinic) and snowball sampling (e.g., were identified by champion as engaged frontline staff), and invited to participate by email. Interview guides inquired about project purpose and main activities, use of tools, (if applicable) coach role, and project penetration and impacts; 18-month interviews focused on sustainability and final reflections on CTAC participation. All interviews were conducted by one of two PhD medical anthropologists (LP, EF) who had no other contact with the site participants. Interviews were audio recorded and transcribed.

For coached sites, the coach team took field notes during initial site visits, which detailed objective observations and reflections on meetings with site participants. Coaches also logged interactions with sites in a Microsoft Access database, documenting each of their weekly coach calls, including who was present, the agenda, what happened, next steps, and reflections on what was challenging and what went well. The coach team also engaged in retrospective, guided debriefs about each site with the lead author (LP). Debriefs concerned site project adoption and implementation process, coaching strategies and challenges, and project outcomes. Debriefs were recorded as detailed summary notes. Coached sites also produced final project reports that described their goals, activities, and outcomes.

### Data analysis

Data were analyzed and triangulated using a matrix approach [28], principally organized by domains from the Consolidated Framework for Implementation Research (CFIR) [29] and Proctor et al. outcome measures (coded broadly as implementation, service, patient, and staff outcomes) [30]. These were supplemented with domains based on preliminary review of a sample of interview data (e.g., characteristics and activities and staff outcomes; see Additional file 3). The data matrix was organized by site; data sources were denoted by text color. Once data was inputted from each data source, for each site, data for each domain was summarized to allow for theming within domains and comparisons across sites.

Interview data were analyzed in Atlas.ti [31] by two members of the evaluation team (LP, PB) using a codebook based on the domains described above. Approximately 10% of the interview data was independently coded by each coder and differences in coding reconciled through discussion. For the remaining data, one person independently coded the data while the second person reviewed coding; coding differences were discussed and coding updated as needed. Code reports for each domain were reviewed and summarized by site, and inputted into the data matrix.

Coach logs and site debriefs were reviewed by the lead author (LP) for data related to barriers and facilitators to implementation, and implementation processes. Site documents were reviewed by two members of the evaluation team (LP, PB) for information related to project goals, implementation activities, and outcomes. Data from these reviews were summarized and inputted by domain into the data matrix.

Because we observed variability in the scope of sites’ unique QI projects, we also retrospectively assessed site QI project complexity by adapting the Complexity Assessment Tool for Systematic Reviews [32] for use outside a systematic review setting. Using the data sources described above, a summary description of each project was created and de-identified. Two members of the evaluation team (LP, IML) reviewed each anonymized project description and cooperatively assessed it using the six core dimensions of the tool: active components, behavior or actions of intervention recipients, organizational levels, degree of tailoring, skill required for delivering the intervention, and skill required for the targeted behavior (Additional file 4). Each domain was assessed on a 0 to 3 scale representing more or less complexity (0 = no project, 1 = simplest form to 3 = most complex form of the dimension). Assessments were discussed with the coach team, who had varying knowledge of participating sites’ projects, to ensure agreement on scoring; no adjustments were requested and the scores were finalized. We used hierarchical linear regression with a random intercept for each clinic pair to model the effect of coaching on project complexity, with the unit of observation being the rating of a clinic on a given attribute of project complexity. The six attributes of each clinic’s project complexity, scored 0 to 3, were treated as repeated measures; total scores for each clinic’s project complexity could range from 0 to 18. (a score of 0 indicated that no project was implemented).

## Results

### Participants

We conducted 79 interviews over time with 45 individuals (see Table 1). On average, people participated in two interviews over the course of the study. We had no interviews with the non-coached site that did not carry out a CTAC project. Most participants were clinic frontline nurses and clerks, and their clinic supervisors; other roles included nurse educators, clinic physicians, and system leadership. We conducted more interviews with coached sites (*n* = 45) than non-coached sites (*n* = 17); we had 17 interviews with system leadership. There were more interviews with coached sites in part because those clinics had larger local CTAC QI teams, resulting in numerically more people to invite for interviews. Members of those teams also had much more contact with CTAC personnel, which may have influenced their willingness to participate in interviews.

**Table 1:** Number of interviews by coaching status and participant type, over time

	6-month interviews	12-month interviews^a^	18-month interviews	Total interviews
Coached site	17	19	9	45
Champions	8	8	8	
Frontline staff	9	11	1^d^	
Non-coached only site^b^	6	7	4^c^	17
Champions	5	5	4	
Frontline staff	1	2	0	
System leadership	10	7^e^	n/a	17

^a^Two interviews at 12 months involved 2 participants each (one non-coached and one coached site)

^b^For one non-coached site, we had no interviews

^c^At 18 months, we did not have interviews with 2 non-coached sites

^d^One interview at 18 months involved a sub-group lead who was not an overall project champion

^e^At 12 months, there were no leadership interviews for 2 of the 5 participating VA systems

### QI projects

Sites undertook a variety of CTAC projects (Additional file 5). Six projects (4 coached sites, 2 non-coached sites) focused on unscheduled patient visits (“walk-ins”). Other projects targeted different issues (e.g., reducing administrative discontinuations of consults, educating patients with pre-diabetes). All but two sites (both non-coached) attempted to introduce a tool (e.g., patient letter and/or brochure, missed opportunity list) and process change (e.g., new workflow).

Projects aligned with clinic concerns, but according to interview data these were not always the top priorities. Three coached sites and one non-coached site had a focus that they identified before participating in CTAC. Two more sites (1 coached, 1 non-coached) selected projects which their clinics had worked on before.

“we were kind of in the process of making a pamphlet and then when my nurse manager had recommended that we do a CTAC project because it met the guidelines and coordination of care” (champion, site 2, coached)

“[my medical director] kind of proposed, hey, why don’t you do this other piece [decrease the number of consults that are not scheduled]. We’ve been talking about how this consult, this (dis) continuation thing was really inefficient and irritating” (champion, site 6, non-coached)

The other six sites (2 coached, 4 non-coached) identified projects after starting CTAC: “the focus was to implement a tool that was available … [co-champion] just kind of brought a copy [of the save a trip form] and said, okay, let’s try this.” (champion, site 3, non-coached).

Reasons for adoption varied. For the 6 sites implementing walk-in projects, people said walk-ins caused staff frustration because they disrupted clinic flow and could delay meeting Veterans’ needs. A champion at a non-coached site explained:

“we decided to implement a form that’s called the Save a Trip form … We also had hoped that giving patients specific phone numbers would stop some of the walk-ins because walk-ins we have to fit them in in the middle of the day and sometimes they jam up our days and sometimes they’re pretty inappropriate in terms of either patients walking in with incredibly overwhelming issues in which we have to send them out 911 or they’re tiny things that could have even been taken care of on the phone. So it’s kind of a dual thing, helping the patients help manage but also helping us control our walk-ins” (champion, site 3, non-coached)

Eight sites used tools from the CTAC toolkit (1 coached site used 2 tools). These included the Save a Trip form (*n* = 3; 1 coached site, 2 non-coached sites), clinic information brochure (*n* = 5; all coached sites), and a patient medication tracker (*n* = 1; non-coached site). Coaches shared tools developed by other coached sites. Coached sites spent considerable time tailoring tools for local clinic use; generally, non-coached sites did not describe adapting tools. Most interview participants either could not recall using the toolkit or remembered looking at it once or twice.

Coached clinics (mean 11.5 project complexity) had more complex projects than non-coached clinics (mean 7.5; difference in score, 4 points; 95% confidence interval 1.75–6.25; *p* < 0.001). (Table 2) Non-coached sites had more variability in and less complexity across almost every dimension compared to coached sites. Coached site projects had more components and more targets. Coached projects were usually focused on standardizing clinic practices rather than tailored delivery. By contrast, several non-coached sites allowed staff to decide how they would operationalize parts of their intervention. All projects required only basic skills (i.e., no additional specialized training) (e.g., handing a brochure to a patient, calling a clinic for a prescription refill) to accomplish the targeted behavior change.

**Table 2:** Site project complexity, comparing coached and non-coached sites across each dimension

Dimension of complexity	Coached Site Mean (Range)	Non-Coached Site Mean (Range)
1. Active components included in the intervention in relation to usual care	2.3 (2–3)	1.5 (0–3)
2. Behavior or actions of intervention recipients or participants to which the intervention is directed	3 (3)	1.5 (0–3)
3. Organizational levels and categories targeted by the intervention	2.5 (2–3)	1.7 (0–3)
4. The degree of tailoring intended or flexibility permitted across sites or individuals in applying or implementing the intervention	1 (1)	1.2 (0–2)
5. The level of skill required by those delivering the intervention in order to meet the intervention objectives	1.7 (1–2)	0.8 (0–1)
6. The level of skill required for the targeted behavior by those receiving the intervention in order to meet the intervention objectives	1 (1)	0.8 (0–1)
TOTAL	11.5 (11–12)^a^	7.5 (0–12)^a^

For each item, projects were assessed on a 1–3 scale, from lower to higher level of complexity; 0 indicates that no project was completed. See Additional file 5

^a^The difference in total score was statistically significant at the *p* < 0.001 level

### Implementation processes

Coached sites engaged in more CFIR implementation processes (planning, engaging, and reflecting and evaluating) than non-coached sites (see Table 3). All coached sites began planning during the coach team site visit and QI teams met weekly with their coach to iteratively plan, reflect on, and evaluate their projects. As part of their coaching process, coaches led their teams through the development of Specific, Measurable, Achievable, Realistic, and Time-bound (SMART) goals.

**Table 3:** CTAC sites’ engagement in CFIR implementation processes, coached versus non-coached

	Coached Sites	Toolkit Only Sites
Champions	1–2 champions, usually a nurse, displayed champion behaviors^a^	1–2 champions, usually a nurse, displayed champion behaviors ^a^
QI Teams	2–8+ people Often composed of nursing and clerk staff, +/− providers, +/− supervisors	1–4 people Often a nurse champion with assistance from a supervisor
Planning	Began between leaders and champions during site visit Scheduled, structured weekly meetings Organized through SMART^b^ goals	Driven by champions with input from supervisors Lack of consistent meetings or organizational structure Generally did not describe having elaborated goals
Engaging	Formal outreach (e.g., scripted education or printed materials) to staff and patients to standardize practice	Utilized informal outreach to notify staff of intervention
Reflecting and evaluating	Developed and/or used structured data collection tools Data primarily fed back to refine tools and processes, less effort to document implementation and impacts	Sometimes used single data points on administrative reports to track impacts and/or informal methods to evaluate Did not describe using data feedback mechanisms to improve tools or implementation

^a^Champion behaviors included but were not limited to serving as a team leader, helping to plan and troubleshooting problems, engaging staff in training and education, and advocating for the local initiative [33]

^b^*SMART* Specific, Measurable, Attainable, Relevant, and Time-Bound

### Champions and QI teams

Each CTAC site identified 1–2 project champions, usually nurses. Most champions expressed ownership over and commitment to their project, and described performing most of the implementation tasks, which sometimes involved engaging others to help. Many champions, regardless of coaching status, were volunteered into their role rather than freely adopting it. Four sites (3 coached sites) had champion disruption or turnover during the project (e.g., a co-champion was detailed to another clinic).

All but one CTAC coached site had large interdisciplinary teams composed of nurses, often clerks, and sometimes physicians; team members sometimes turned over and were more or less engaged (e.g., by attendance in meetings, taking on tasks). Most of the non-coached projects were accomplished by a champion, who periodically had guidance or material help from a colleague; in two cases, there were small, interdisciplinary working groups.

### Planning

Coached sites had weekly, moderated team meetings during which they developed action plans, tailored tools to implement, created new workflows, planned implementation activities, and distributed tasks. Non-coached projects generally lacked protected time for people to come together to plan; when they did, they often were periodic in the first couple months of active implementation and then ceased.

### Engaging

Coached sites described more formal mechanisms for engaging staff and patients. This involved developing materials and setting aside time for staff education (e.g., in-service to introduce a new form, distributing pre-diabetes education packets, creating scripts for staff to use when giving patients information). Often these efforts were intended to standardize the delivery of the intervention.

Champions at non-coached sites described informally engaging staff through one-on-one conversation or introducing a new form or process at a meeting; they did not usually emphasize standardizing practice and two sites explicitly allowed individuals and clinic teams to determine tool use.

### Reflecting and evaluating

Coaches walked teams through testing, evaluating, and tailoring the tools they implemented. Coached sites tracked and assessed the most common reasons for walk-ins, conducted usability testing with patients and staff on clinic brochures, received input and feedback from staff on new workflows, and did pre- and post-tests to assess the impacts of patient education. The amount and depth of data collection sites conducted varied. Due to data fatigue and/or lack of time, most sites did little to evaluate impacts after implementation. All coached sites, with the guidance and assistance of their coach, produced midterm and final project reports that described their goals, activities, assessments, and reflected on lessons learned and plans for sustainability. One coached site formally presented findings to leadership at 6 months (as part of an ask to change clinic hours) and another presented to its leadership at 12 months.

Most non-coached sites did not collect their own data. Three sites used administrative data to track impacts. Champions described checking in with staff to see how things were going (in at least one case, the CTAC evaluation interviews motivated check-ins) or, in one case, relying on administrative data only? to evaluate impacts. Usually sites were looking at single data points and generally did not describe discussing the data in a group or using it to alter implementation. One non-coached site formally presented its findings to leadership at 12 months.

### Facilitators and barriers

Regardless of coaching status, sites reported barriers and facilitators related to readiness for implementation, networks and communication, champions, and personal attributes (see Additional file 6). Some factors, such as leadership engagement, team QI experience, and team turnover, could either help or hinder implementation depending on timing (e.g., after key decisions were already made) and interaction with other factors (e.g., CTAC’s QI model, team dynamics).

### Coaching

**Fig. 2:**
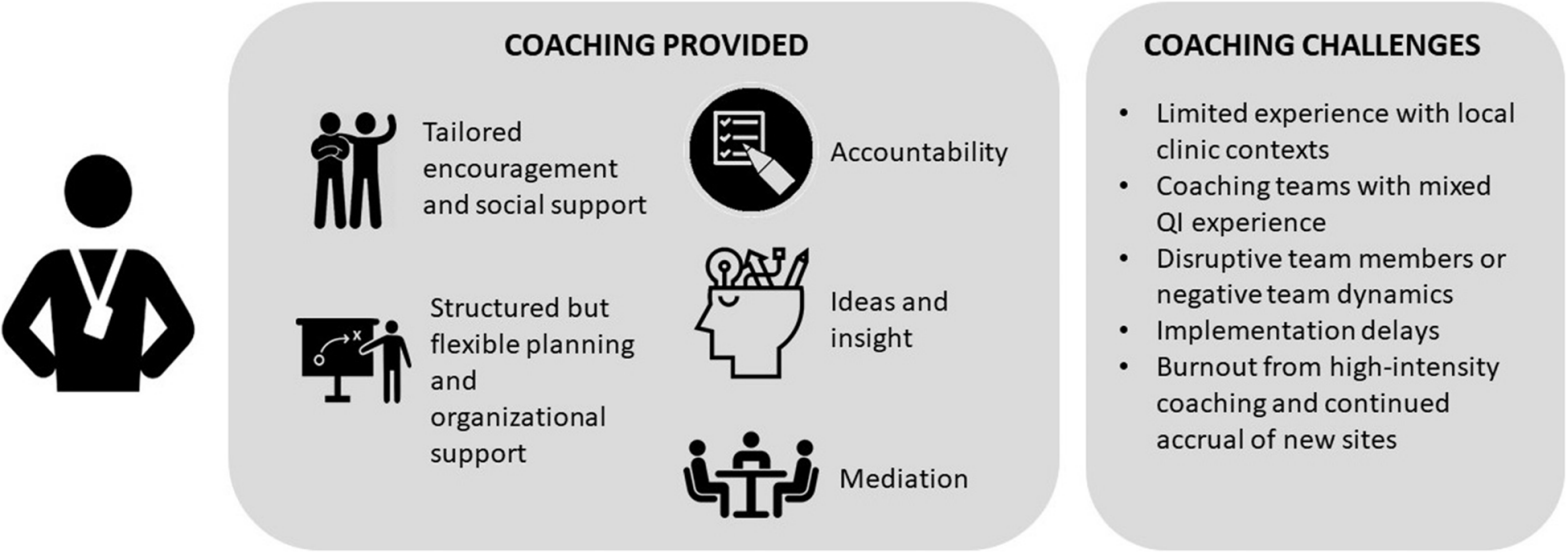
CTAC coaching activities and support as reported by participants, and coaching challenges

Coached participants generally found coaching valuable (Fig. 2). Coaches provided social support and mediated group dynamics (e.g., bridged silos), while offering administrative support, generating new perspectives, and fostering accountability. Coaches hosted weekly meetings, which kept teams on the same page, and focused on tasks, and projects moving:

“[the weekly meeting with the coach] was a time where we could all be together on the phone to discuss the plan for the project. So I think she really did a great job at bringing everyone together ... I think the project would not have come very far if it wasn’t for the coaching.” (931–2 12 m)

The coaching manual was a resource rather than a strict blueprint for coaching. Some elements, such as having the sites complete action plans (including SMART goals) and mid-point and final reports, were conducted across coached sites. These activities drove planning, reflecting and evaluating, and instilled accountability. Coaches selected other manual components (e.g., report templates, process mapping using Microsoft Visio) to address site issues. The coaches observed and debriefed after coach calls, and learned from, problem-solved, and provided social support to each other [34]. The weekly coach team meetings were an additional opportunity to describe and reflect on progress, discuss how to move forward, and maintain fidelity to certain CTAC components (e.g., action plans and reports).

Using action plans, coaches broke down QI steps for teams. Participants were surprised that seemingly simple projects could involve many steps. Over time, coaches honed and adapted similar product development and evaluation tools (e.g., brochure and walk-in tracking forms, patient and staff feedback forms), and products (e.g., patient education handouts, staff workflows, patient outreach letters) that they shared when relevant to a site’s focus.

For some staff, coaches could overcomplicate projects by suggesting what was perceived to be unnecessary data collection and iterations of tool development that enhanced project demands. At one site, champions expressed concerns about the time taken up by planning “paperwork” (action plans):

“we have highly, highly educated, highly proficient, highly eager people to work on these projects. … one of my recommendations to the facilitator of the CTAC call is, that I don’t want to lose the momentum. … I know with our group here successes are a huge thing and if people can see those they’re willing to do more.” (354–1 6 m)

During site debriefs, coaches discussed facilitation challenges similar to those described by participants: limited coach experience with clinic teams’ context; adapting coaching to team members with the least QI experience, which could make the process feel too slow for more experienced members; and disruptive team members or negative team dynamics that could impede progress. Delays made it take longer than expected to complete certain processes, leaving little time at the end of the 12 months to fully evaluate implementation impacts and plan for sustainment. However, learning from experiences with early sites fed back into coaching for later sites (e.g., updating action plans in parallel with implementation instead of a standalone first step). Coaches described the cumulative positive benefits (e.g., gained confidence, knowledge and skills) and negative impacts (e.g., feeling burned out from the intensity of effort to gain buy-in and adapting to teams and projects) of having a new site to coach every few months.

### Project-specific implementation impacts

Sites’ reports of impacts varied in detail given that teams undertook site-specific projects and may or may not have monitored project-specific outcomes (see Additional file 5). Across sites, we assessed implementation penetration within and outside the clinic at 12 months and sustainability at 18 months, project-specific outcomes (often a service or patient outcome), and staff-reported outcomes (see Table 4).

**Table 4:** CTAC sites’ project outcomes, coached versus non-coached

	Coached Sites	Non-Coached Sites
Implementation outcomes at 12 months		
Penetration	Half of sites fully implemented across their clinic	Half of sites fully implemented across their clinic
	Other half had implemented most of their projects, but had components of their complex projects still being worked on or that had been infeasible to implement	Other half either had not implemented anything or conducted limited pilots (at 12 months, one pilot was adapted to be implemented clinic-wide)
Fidelity to site project	Good but some variable fidelity across clinic	Good but some variable fidelity across clinic
Implementation outcomes at 18 months		
Sustainability	Variable, but most components still in use, though some drop-off in practice	Variable, but most components still in use, though some drop-off in practice
Dissemination	Ongoing work to monitor, adapt, and spread projects	Little ongoing work, several projects being fully implemented and one targeted for system-wide spread
Project reported outcomes		
Service and patient	Staff and patient satisfaction with intervention Some tracking that identified positive service impacts	Did not systematically collect satisfaction data Few described assessing impacts, but overall perceived positive impacts
Staff outcomes	Improved clinic communication and relationships, gains in QI knowledge and skill, and a sense of pride and accomplishment	When learning was described it was often in relation to content of their intervention

By the end of 12 months of active implementation, three coached and three non-coached sites had fully implemented their projects. Regardless of coaching status, fidelity across clinic teams was variable (see Additional file 5). The three coached sites that did not achieve full project implementation had implemented most of their projects, but some components were still being worked on (e.g., patient education about labs orders), had been piloted or developed but not approved for full implementation (e.g., extended clinic hours), or were not feasible to implement (e.g., new medication list in the electronic health record). Among non-coached sites that did not achieve full implementation, one site did not implement anything, another conducted a two-week pilot, and a third piloted a process that it decided to implement in an adapted form at the end of 12 months.

At 18 months, regardless of coaching status, there was varying sustainment of tools and processes. Drop-offs of some of the project components were due to, for example, no one being responsible for assuring continued use, or a newer tool superseding use of the project tool. However, seven clinics had ongoing monitoring, active adaptations, and/or spread efforts related to their projects. In five clinics (four coached sites), specific individuals were assigned to support continued tool use (e.g., updating forms, order copies), metrics were routinely reviewed with other clinic measures, or the tool or process had been enfolded into an existing clinic process (e.g., new patient orientation). It was unclear, however if these activities were adequate to sustain implementation (e.g., by identifying and responding to emergent problems). One non-coached site was planning to implement its pilot project across its clinic and another non-coached site’s form was to be spread systemwide. Ongoing work to adapt, implement, or spread seemed to be related to the tool or process having a champion and/or leadership encouragement (which required an engaged leader who was aware of the project). There was no clear relationship between active spread and evidence of impact on patient or clinic outcomes. Some tools (forms or brochures) were also passively spread when staff from other clinics saw them and asked for a copy.

As noted, coached sites formally collected and analyzed data, especially during tool and process development. These measures included feedback from staff and patients, which was generally positive. Four coached sites also reported positive service impacts (e.g., reduced volume of unscheduled nursing visits). Three non-coached sites reported service impacts (one for a limited pilot); two showed positive impacts and one had null findings.

During interviews, participants described other impacts. Coached sites mentioned improved communication and relationships among clinic staff. This was especially true for the five clinics that implemented projects related to unscheduled visits, where new workflows required nursing and clerk staff to come together to discuss roles, current processes, and problem-solve improved processes. In these clinics, there was a better sense of shared mission, respect, and “unity.” Coached participants, especially champions, reported professional development from the mentoring and modeling provided by their coach, and hands-on engagement in their QI projects. They also described feeling pride and accomplishment. When describing learning, non-coached participants discussed it in relation to the target of their intervention (e.g., how “no-shows” can negatively impact patient care).

## Discussion

Coached and non-coached sites had distinct implementation patterns that showed how coaching influenced choice of projects, implementation and evaluation approaches, and to a lesser extent, sustainment. Coached sites engaged a numerically larger group of people from more diverse clinic roles in the project team, and had projects that often involved the coordination between nurses and clerks, and utilized more formal methods (e.g., in-service training, scripts) to standardize clinic practices. Teams at coached sites tailored tools to the local site (e.g., usability testing), gained staff buy-in, and ensured their interventions were acceptable to staff and patients. In sustainment, coached sites had mechanisms in place to monitor, and had efforts to adapt or spread their interventions. Non-coached site projects were more variable, and tended to be simpler in terms of organization (often relying on planning and action by champions rather than a team), implementation (informal engagement and tracking), and efforts to sustain. Regardless of coaching status, sites did little to assess impact but those measures tended to be positive where documentation was available. Coached sites emphasized additional staff-related impacts (e.g., improved relationships, QI skills).

Fidelity to the original expectations of the CTAC project was limited across sites. For example, we expected that both coached and non-coached sites would collectively draw on a variety of tools from the toolkit, and that coaching would facilitate tool implementation and potentially help bridge multiple care pathway gaps. Although more coached sites (*n* = 5) used tools from the toolkit than non-coached sites (*n* = 3), the toolkit was not a resource that any site consistently drew from or that participants easily recalled during interviews. Among coached sites, tool adoption was mediated by the coach, but was limited primarily to one tool (i.e., the clinic brochure), which coaches helped adapt at one site and then shared and helped further tailor with subsequent sites. This finding is consistent with that of previous studies that identified variable toolkit uptake [19], challenges to primary care practice implementation of tools from toolkits that were similar to barriers we identified (e.g., competing demands, technological challenges, team dynamics) [21, 35], and illustrated how external QI experts can assist practice toolkit use [21].

The lack of fidelity to the original expectations was a combination of “status quo bias” (selection of projects that were already in progress or that were most convenient given available staff), the predominance of certain problems (e.g., unscheduled patient visits), and a tendency to gravitate toward simpler projects and tools, either by choice or by necessity (e.g., more complex projects could not be accomplished as planned, resulting in a reversion to simpler projects). Giving sites flexibility to adopt projects that aligned with their perceived needs helped with initial buy-in and motivation, but resulted in projects of more limited scope than expected. The few coached sites that attempted projects requiring broader systems change (e.g., expanded clinic hours, establishment of a triage nurse, improving a medication note template in the EHR), were unsuccessful even with support of CTAC to engage the correct stakeholders. In CTAC, because sites chose their own projects, early months were often spent deciding on and honing targets; 12 months was not always enough to plan for and engage stakeholders needed for complex changes that were outside the CTAC toolkit. The findings of this study suggest that coaching clinic-based teams may be inadequate for achieving complex change, and may require broader efforts to engage across the larger system to be successful.

While coached sites developed more complex, tailored interventions and implemented their projects in a standardized way, beyond patient acceptability, patient impacts (e.g., on patient experience of care) were unclear. In parallel with findings from prior work [36], the tools that sites developed (e.g., clinic brochures) were generally low-intensity patient education, which placed the impetus for care coordination on patients rather than engaging patients as partners in care. These were often readily accepted by staff and perceived to be achievable and to address pressing clinic organizational issues. These examples suggest that adoption targets may be selected in large part because of perceived feasibility rather than impact. Workflow changes, for which buy-in was harder, focused on nurse and clerk workflows, but did not change primary care provider or cross-setting practice (though could potentially impact experiences of providers or staff in other settings, such as through patients who reported and requested medications differently). This emphasis on clinic-based solutions may reflect the fact that both coached and non-coached teams, in all their variety, were based in single clinics; achieving cross-setting and organization-level solutions to improve care coordination, given the complex dynamics of those systems, may require broader teams and time and resources for relationship building [37].

Although these findings do not support a direct link between activities undertaken by coached or non-coached sites and the modest improvement in patient experience of care noted at both groups of sites [25], our observations suggest that coaching catalyzed an investment in relationships and team-building among coached site staff that may support improved staff morale and team function. This is consistent with Crabtree and colleague’s work emphasizing the importance of attending to the quality of the interactions among staff and addressing dysfunctional relationships in primary care transformation [38]. The coach-led weekly meetings generally involved multidisciplinary QI teams and space for people to talk who did not normally talk, which created opportunities for sensemaking and learning [39, 40]. These meetings created a scaffolding to plan and reflect, to hold people accountable and move the project forward, and to foster cross-service collaboration. Such meetings were important for building relationships and communication pathways, which have implications for the broader effort to improve care coordination and innovation within these clinics [41]. This investment in multidisciplinary teams may have longer term impacts on communication and coordination efforts.

This evaluation has several limitations. First, we had more details about what happened at the coached sites than the non-coached sites. We mitigated this by focusing on domains for which we had comparable information across sites and not assuming activities did not occur at non-coached sites just because we did not hear about them. Second, our evaluative approach was primarily descriptive, and not designed to determine whether the results achieved through coaching warranted the resources devoted to it. Future sites can use our characterizations of how coached and non-coached sites implemented their projects to weigh the benefits and drawbacks of investing in coaching. Third, the unique design of the CTAC project (e.g., the flexibility that sites had in choosing their projects) means that our findings may not generalize to more structured interventions. However, we think that CTAC’s design generalizes to important initiatives such as QI collaboratives, where many different QI activities may take place under a broad thematic umbrella. Fourth, CTAC was implemented within clinics using the VA patient centered medical home model and within integrated systems that often had some QI capacity (even though we often found that at the clinic level, team dynamics and QI experience were highly variable). This may limit generalizability to primary care settings with less integration, team development, and experience with QI.

## Conclusion

In conclusion, we found that relative to the experience at non-coached sites, coaching had a positive influence on QI skills of coached sites’ team members, relationships between team members at a site, and the scope and rigor of projects that teams carried out. Coaching provided structure to support, guide, and hold team members accountable. However, both coached and non-coached sites often implemented projects focusing on patient education and self-management, meaning that key care coordination challenges known to affect patient experience of care (e.g., improving care coordination across settings) were not attempted. A year’s time was potentially too short to enact cross-setting and structural change given the relationships that had to be built and competing priorities within these settings. Future work will more deeply explore the strengths and limitations of the coaching model employed within CTAC to better understand the scenarios in which it can best be deployed.

## Supplementary Information

**Additional file 1.** Summary of tools in the CTAC toolkit. Table describing the types of tools in the CTAC toolkit.

**Additional file 2.** CTAC coaching manual. Coaching manual developed for and used by CTAC coaches during sites’ active implementation.

**Additional file 3.** CTAC codebook. Codebook used for analyzing qualitative data.

**Additional file 4.** Guide for Assessing Site CTAC QI Project Complexity. Dimensions and criteria used to assess the complexity of sites’ CTAC projects.

**Additional file 5.** Site project and outcomes table. Summary table of each CTAC site’s project, use of CTAC tools, project complexity rating, and reported project outcomes at 12 months, and implementation outcomes at 12 and 18 months.

**Additional file 6.** Barriers and Facilitators to Project Implementation. Detailed description of the barriers and facilitators sites encountered when implementing their QI projects.

## Data Availability

The datasets generated and/or analysed during the current study are not publicly available due to lack of participant consent to share interview transcripts and transcripts in repositories. De-identified administrative datasets may be eligible for future data sharing once national VA guidance on request and distribution processes are provided (in process). Final datasets will be maintained locally until enterprise-level resources become available for long-term storage and access.

## Abbreviations

CFIR: Consolidated Framework for Implementation Research
CTAC: Coordination Toolkit and Coaching
QI: Quality improvement
SMART: Specific, Measurable, Achievable, Realistic, and Time-bound
VA: Veterans Health Administration
