# Supplementary material for: Toolkit and distance coaching strategies: a mixed methods evaluation of a trial to implement care coordination quality improvement projects in primary care
Source: BMC Health Serv Res. 2021 Aug 14;21:817. doi: 10.1186/s12913-021-06850-1 (PMC8364700; doi:10.1186/s12913-021-06850-1)
Supplement: Supplementary file 1 — Additional file 1. Summary of tools in the CTAC toolkit. Table describing the types of tools in the CTAC toolkit. [file 12913_2021_6850_MOESM1_ESM.zip › Additional File 1.docx]

Additional File 1. Summary of tools in the CTAC toolkit (from Noël et al. 2021)

| **Tool Category** | Description of Tool |
| --- | --- |
| **After-Visit Instructions for patients (electronic)** | An electronic tool that produces a customizable, printable patient summary that can be provided to a patient after his/her primary care office visit to summarize visit content and subsequent action steps, if appropriate. |
| **After-Visit Instructions for patients (paper)** | A customizable document instructing patients about where to go before leaving the VA the day of their appointment, as well as any follow-up actions which need to be taken. Includes contact information for specialty clinics as well as a map of the campus, which can be used to direct patients to their next destination on campus. |
| **AudioRENEWAL: Phone-based Medication Renewal for Patients** | Allows patients to request a renewal of their prescription directly from within the AudioCARE telephone refill system 24 hours a day, 7 days a week. |
| **Clinic Information Brochure** | A template that clinics can customize to create a clinic information pamphlet for patients that includes pertinent information about the clinic.  Information may include appointment-making instructions, provider contact information, prescription refill instructions, and other clinic details. |
| **Communication with Community Providers to Co-Manage Veterans’ Care** | A two-page document that includes a letter to help facilitate communication between a VA provider and an outside healthcare provider regarding management of the Veteran patient and instructions for obtaining medications through the VA, and a guide for providers (non-VA) to request a prescription medication that is non-formulary. |
| **Consult Guides for Primary Care (Information to Include)** | A comprehensive list of clinical conditions and for each, a standard set of information to be included in a referral request, to help ensure a meaningful visit when the referred patient visits the relevant specialist. |
| **Medication Tracker for Patients** | A one-page form with sections to be filled in by a member of the PACT team for the patient. Spaces are provided to fill in a patient's medication details and healthcare provider name and contact information.  The medication list includes details that can help patients remember when to take their medications and what dose to take. |
| **My Primary Care Team** | Contact and Other Information: A one-page handout that lists important provider contact information for a patient.  Includes contact details for the primary care provider, PACT nurse, tele-health nurse, specialty care, pharmacy, and mental health. |
| **My Primary Care Team - Wallet Card and Contact Information** | A customizable wallet-sized card that can be printed, populated with a patient's primary care team information and given to the patient.  The back of the card includes a list of information patient's should provide when leaving a message at the clinic. |
| **Patient Agenda Setting Form** | A simple, easy to fill-out form to help patients prepare for their healthcare visit.  The form can be sent to patients in the mail with a pre-visit packet, or can be given to patients to complete in the waiting room. |
| **Patient Treatment Decision Guide** | A worksheet for patients to use when faced with a medical decision.  The form includes questions to ask the provider during the medical appointment, space to take notes, and guidance on how to proceed with making a decision. |
| **Pharmacy Safety for Patients** | A patient hand-out that explains the role of the pharmacist, pharmacy and patient during the process of obtaining medications. |
| **Relaxation and Meditation Program: An approach to self-management of mental health in primary care** | Information and tools for implementing a program that teaches patients relaxation and meditation skills, with the potential benefit of decreasing the number of referrals to specialty mental health services. |
| **Save a Trip to Primary Care** | A one-page resource for patients, explaining and illustrating the difference between routine, non-urgent, and urgent medical situations.  The sheet explains to patients what actions to take in the case of each of these situations, and provides relevant contact information. |
| **Tips for Patients: Improving Communication with your Primary Care Team** | A two-page handout that can be mailed to patients in a pre-visit packet or given to patients at check-in, before their appointment.  The tool includes tips for communicating with the primary care team, questions for patients to think about before their appointment and space to write down answers.  This tool can help ensure all of a patient’s questions and concerns are addressed during their medical appointment. |
| **Tips for Patients: Questions to ask Before, During and After your Visit** | A guide that includes tips, ideas, and questions for patients to use before, during, and after their appointment that will help facilitate a successful healthcare visit. |
| **VA Care Coordination Service Agreement Template** | A template with the purpose of facilitating timely access and patient-centered care for patients by promoting an effective relationship between the PACT team and the specialty care team during the management of a patient’s care. |
| **VA Formulary (Abbreviated)** | The Primary Care Service in the VA Palo Alto Healthcare System worked with its pharmacy department to create an abbreviated and searchable VA formulary. This resource was posted on the healthcare system’s external web page and is updated periodically for accuracy. |

Noël, P.H., Barnard, J.M., Leng, M. *et al.* The Coordination Toolkit and Coaching Project: Cluster-Randomized Quality Improvement Initiative to Improve Patient Experience of Care Coordination. *J GEN INTERN MED* (2021). https://doi.org/10.1007/s11606-021-06926-y
