# Supplementary material for: Toolkit and distance coaching strategies: a mixed methods evaluation of a trial to implement care coordination quality improvement projects in primary care
Source: BMC Health Serv Res. 2021 Aug 14;21:817. doi: 10.1186/s12913-021-06850-1 (PMC8364700; doi:10.1186/s12913-021-06850-1)
Supplement: Supplementary file 2 — Additional file 2. CTAC coaching manual. Coaching manual developed for and used by CTAC coaches during sites’ active implementation. [file 12913_2021_6850_MOESM2_ESM.docx]

Additional file 2. CTAC coaching manual

This file contains the CTAC coaching manual. It has been anonymized for public distribution. Material linked to in the manual may have been changed, removed, or moved since the manual was last updated in October 2018. Links within the VA domain may be inaccessible outside the VA firewall.

*
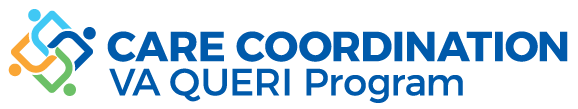
*

Coaching Manual

Coordination Toolkit and Coaching (CTAC) Project

*For more information on the Care Coordination QUERI program, visit* <http://www.queri.research.va.gov/programs/pact.cfm>

Prepared by:

The Coordination Toolkit and Coaching (CTAC) Project ,

part of The Care Coordination QUERI Program^[[1]](#footnote-1)^

We want to acknowledge the sources^[[2]](#footnote-2)^ we used to develop this manual, including:

- VA Office of Academic Affiliations (OAA) Centers of Excellence in Primary Care Education (CoEPCE) Implementation Kit
- VA Geriatric Scholars Program: The Foundations of Health Quality Improvement
- VISN 22 PACT Demonstration Laboratory (VAIL) Evidence Based Quality Improvement (EBQI) Toolkit
- National Center for Patient Safety’s Virtual Breakthrough Series (NCPS)
- Implementation Facilitation Training Manual Revisions Workgroup, Chair QUERI for Team-based Behavioral Health

Table of Contents

[Overview 6](#_Toc486409270)

[Organizational Readiness 6](#_Toc486409271)

[Leadership Support 7](#_Toc486409272)

[Coaching Roles and Responsibilities 7](#_Toc486409273)

[Project Timeline and Steps 11](#_Toc486409274)

[Project Timeline & Gantt Chart 11](#_Toc486409275)

[**I.** **MONTHS 0 – 2** 11](#_Toc486409276)

[**II.** **MONTHS 3 – 9** 12](#_Toc486409277)

[**III.** **MONTHS 10 – 12** 12](#_Toc486409278)

[Project Steps 12](#_Toc486409279)

[**A.** **Team building** 12](#_Toc486409280)

[**B.** **Tool Identification** 14](#_Toc486409281)

[**C.** **Educate the team on quality improvement change processes** 14](#_Toc486409282)

[**D.** **Change Management** 19](#_Toc486409283)

[**E.** **Measurement** 20](#_Toc486409284)

[**F.** **Plan-Do-Study-Act (PDSA) cycle** 23](#_Toc486409285)

[**G.** **Sustainability and Spread** 25](#_Toc486409286)

[Project Evaluation 26](#_Toc486409287)

[**A.** **Ongoing Evaluation (Informal & Coach Directed)** 27](#_Toc486409288)

[**B.** **Mid-Point and Final Reports** 27](#_Toc486409289)

[Tips for Facilitating Effective Meetings 27](#_Toc486409290)

[**A.** **Tips for Facilitators** 27](#_Toc486409291)

[**B.** **Language to Support Structure** 28](#_Toc486409292)

[**C.** **The 4 Ps** 28](#_Toc486409293)

[**D.** **Interactivity** 29](#_Toc486409294)

[**E.** **Handling Common Challenges** 30](#_Toc486409295)

[Summary 33](#_Toc486409296)

[Management Tools 35](#_Toc486409297)

[Tool A: Organizational Readiness Interview Guide 38](#_Toc486409298)

[Tool B: Organizational Readiness Checklist 42](#_Toc486409299)

[Tool C: Introduction and Overview for Stakeholders 43](#_Toc486409300)

[Tool D: Leadership Support Assessment 44](#_Toc486409301)

[Tool E: Business Case Form 45](#_Toc486409302)

[Tool F: Site Visit Agenda 46](#_Toc486409303)

[Tool G: CTAC Project Staff List 48](#_Toc486409304)

[Tool H: CTAC Project Description 49](#_Toc486409305)

[Tool I: Quality Improvement Project Proposal Template and Sample 51](#_Toc486409306)

[Tool J. Patient Experience Survey (Hassles Scale) 53](#_Toc486409307)

[Tool K: Tool Catalog 57](#_Toc486409308)

[Tool L: CTAC Project Timeline for Participating Sites 59](#_Toc486409309)

[Tool M: Sample Weekly Timeline and Plan (Weeks 1-8) 63](#_Toc486409310)

[Tool N. Meeting Call Log 65](#_Toc486409311)

[Tool O: Quarterly Leadership Call Topics 68](#_Toc486409312)

[Tool P: Quality Improvement Project Action Plan Template and Sample 69](#_Toc486409313)

[Tool Q: SMART Goals Template and Sample 86](#_Toc486409314)

[Tool R: A3 Worksheet 88](#_Toc486409315)

[Tool S: Mid-Point Project Report Template 89](#_Toc486409316)

[Tool T: Final Project Report Template 90](#_Toc486409317)

[Tool U: Clinical Query Form 91](#_Toc486409318)

[Tool V: Team Contact and Information Sheet 92](#_Toc486409319)

[Tool W: Interdisciplinary Team 93](#_Toc486409320)

[Tool X: Quality Improvement Process 95](#_Toc486409321)

[Tool Y: Stakeholder Analysis 97](#_Toc486409322)

[Tool Z: PDSA Worksheet for Testing Change 100](#_Toc486409323)

[Tool AA: Current Process Analysis 101](#_Toc486409324)

[Tool BB: Assessing Staff Education and Training 103](#_Toc486409325)

[Tool CC: Managing Change Checklist 104](#_Toc486409326)

[Tool DD. Implementation Checklist 105](#_Toc486409327)

[Tool EE. Assigning Responsibilities 106](#_Toc486409328)

[Tool FF. Staff Roles 107](#_Toc486409329)

[Tool GG. Sustainability Tool 108](#_Toc486409330)

[Appendix A: Resources 110](#_Toc486409331)

# Overview

This coaching manual was created for the QUERI Coordination Toolkit and Coaching Project (CTAC), part of the larger Care Coordination QUERI program (<http://www.queri.research.va.gov/programs/pact.cfm>). The QUERI program is funded by the Veterans Health Administration’s (VHA) Health Services Research & Development Service’s (HSR&D’s) Quality Enhancement Research Initiative (QUERI) with the overall objective of improving patient-centered care coordination for high-risk Veterans in the VA’s patient-centered medical home model, known as Patient Aligned Care Teams (PACT). The program is a collaboration between investigators in the VA Greater Los Angeles Healthcare System, the VA Palo Alto Health Care System, the South Texas Veterans Health Care System (San Antonio, TX), and the VA Center for Applied Systems Engineering in the Richard L. Roudebush VA Medical Center (Indianapolis, IN). The CTAC project compares the use of an online toolkit alone versus use of the online toolkit plus distance coaching as ways to help PACT improve patients’ experience of care coordination. The project focuses on care coordination in outpatient settings (e.g., between PACT, the Veteran, specialty care, and care in the community).

This coaching manual is designed to be used by the distance coaches for the CTAC project, to guide multi-disciplinary primary care quality improvement teams at geographically dispersed VA clinics over a one (1) year period in the following:

- Identifying a care coordination issue or problem at their facility or clinic
- Choosing one or more tools to implement from the Care Coordination Toolkit to address the identified care coordination issue. (Additional tools may be found or developed if needed)
- Planning and implementing a quality improvement strategy to implement the tool(s)
- Evaluating whether the implementation is successful and whether the tool(s) results in improvements in care coordination

The manual includes guidance, resources and tips that may be useful for addressing quality improvement challenges at each of the coached sites.

While the manual is primarily meant to be used by the CTAC distance coaches and in conjunction with the Care Coordination Toolkit, it can be used by others to facilitate quality improvement in their own ambulatory healthcare settings.

# Organizational Readiness

The CTAC distance coaches may be responsible for helping to assess the readiness of a potential clinic site. Readiness interviews will have likely taken place with each new site prior to the start of coaching, but a situation could arise in which the coach may need to administer an organizational readiness interview.

- [**Tool A: Organizational Readiness Interview Guide**](#_Tool_A:_Organizational_1)

In addition, the Organizational Readiness Checklist may come in handy during the project period if a new champion joins the project, if the scope of the project changes significantly, or if a change occurs that prompts a re-evaluation of the readiness of the organization.

- [**Tool B: Organizational Readiness Checklist:**](#_Tool_B:_Organizational) A checklist that can be used to monitor progress on completing organizational readiness activities.^[[3]](#footnote-3)^

# Leadership Support

Before distance coaching begins, CTAC investigators will have recruited a facility or site to participate in the project. The recruitment process includes multiple phone conferences between the CTAC team and various levels of leadership at the facility. Agreement by leadership to participate in the project includes:

- Approval of the randomization of pairs of the facility’s clinics; within each pair, one clinic will be coached and one will have access to the toolkit only.
- Identify and appoint a champion for the coached site. The champion will have protected time for convening multi-disciplinary teams to work on a care coordination quality improvement project at the site.
- Approval for Veterans at the facility to be contacted (by mail and phone) by a vendor under contract with CTAC, and invited to participate in a patient experience survey.
- Approval by and signature of the facility director on a letter of endorsement for the patient experience survey.

This manual does not focus on VISN, facility or site recruitment or on securing leadership support, but should there be a need to re-secure leadership support, this manual includes the following tools to assist^[[4]](#footnote-4)^:

- [**Tool C: Introduction and Overview for Stakeholders:**](#_Tool_C:_Introduction_1) A letter template to complete and send to key players in your facility or clinic to introduce them to the goals and purpose of your project.
- [**Tool D: Leadership Support Assessment**:](#_Tool_D:_Leadership) A checklist that can be used to assess senior leadership support for the program.
- [**Tool E: Business Case Form**:](#_Tool_E:_Business) A tool to create a high-level overview of the business case for the project that can be presented to senior leaders.

# Coaching Roles and Responsibilities

The two CTAC distance coaches will coach three sites each (total of six coached sites), through a quality improvement project, which the sites will conduct in their local primary care setting.

The coaches will help motivate and guide individual champions at the clinic sites through adoption of new tools to improve patients’ experience of their care coordination. Most of the coaching will be done remotely (via videoconference, webinar, or phone conferences) with one face-to-face kick off meeting per coached site. Each CTAC coach will be supported by clinical content experts on the CTAC team, including physicians, who can join calls with sites as needed. This section of the manual is divided into the two parts for which the CTAC coach is primarily responsible: the site visit and the project period.

**Site Visit**

Together with the CTAC Principal Investigator, the coach is responsible for leading one in-person kick-off site-visit meeting for each coached clinic. The purpose of the site visit is to establish rapport with the coached site and meet with site leadership to ensure agreement on the roles and responsibilities of site personnel and the CTAC team in the project. A typical site visit will include a tour of the site, meetings with site leadership, including facility leaders if possible (e.g., Chief of Staff, facility director), a discussion of the care coordination needs of the coached site, and identification of a clinical champion to serve as a liaison with the CTAC coach.

In preparation for the site visit, the CTAC coach will lead the following activities:

1. With the CTAC team and the site contact, identify potential participants in the site visit.
2. With the site contact, schedule meetings and/or facility tours for the site visit date.
3. Prepare materials for presentation during the site visit. The site visit packet may include:

- [**Tool F: Site Visit Agenda**](#_Tool_F:_Site)
- [**Tool G: CTAC Project Staff List**](#_Tool_G:_CTAC)
- [**Tool H: CTAC Project Description**](#_Tool_H:_CTAC)  – a one-pager that can be distributed to clinic staff during the site visit.
- Site performance reports on quality measures related to care coordination.
- [**Tool I: QI Project Proposal Template and Sample**](#_Tool_HI:_Quality)
- [**Tool J: Patient Experience Survey (Hassles Scale)**](#_Tool_J._Patient_1)
- [**Tool K: Tool Catalog**](#_Tool_K:_Tool_1)
- [**Tool L: CTAC Project Timeline for Participating Sites**](#_Tool_L:_CTAC_1)

1. The coach should also bring with them:

- Business cards
- Site contact information

**Project Period**

The coach is responsible for guiding the site through the quality improvement project, over a 12-month period. Responsibilities include:

- Weekly conference calls with the project site champion and team
  - *Purpose*: Progress/barrier updates and keeping team on track.
  - An example of a timeline and plan for the weekly conference calls with the sites is provided as [**Tool M: Sample Weekly Timeline and Plan (Weeks 1-8)**](#_Tool_M:_Sample).
  - [**Tool N: Meeting Call Log**](#_Tool_N._Meeting) – an example of a meeting call log to keep track of project progress.
- Quarterly conference calls with the site team and site leadership
  - *Purpose*: Keep site leaders informed of project process and keep them apprised of any changes or issues.
  - A suggested schedule of topics for the quarterly leadership calls is provided as [**Tool O: Quarterly Leadership Call Topics**](#_Tool_O:_Quarterly).
- Assist sites in the completion of the following planning documents:
  - [**Tool I: Quality Improvement Project Proposal Template and Sample**](#_Tool_F:_Sample)^[[5]](#footnote-5)^: A basic outline of the project including problems, goals, resources, and measures.
  - [**Tool P: Quality Improvement Project Action Plan Template**](#_Tool_H:_Quality)^[[6]](#footnote-6)^: A detailed description of the project including SMART goals and measures.
    - [**Tool Q: SMART Goals Template and Sample**](#_Tool_Q:_SMART). SMART Goals are^[[7]](#footnote-7)^:
      - **Specific**: well-defined and detailed information about project tasks
      - **Measureable**: to inform how change will occur; numbers and measures
      - **Achievable**: feasible within the proposed timeframe
      - **Realistic**: details about potential constraints and resource limitations
      - **Time-Bound**: a time frame for the goal
    - For additional resources about SMART goals, visit the CDC’s Public Health Information Network Communities of Practice website at <https://www.cdc.gov/phcommunities/resourcekit/evaluate/smart_objectives.html>
  - [**Tool R: A3 Worksheet**](#_Tool_R:_A3): A document created by the New England VERC that helps to identify a problem statement, aims, goals, current state, target state and plan of action.
- Assist sites through each step of the QI project process, clarification of QI concepts, and problem-solving.
  - Save emails and notes from phone communication between the coach and the site team. Important communication and progress will happen between team meetings. These records may be helpful during the evaluation stage of the project.
- Guide team discussions and help the team get results. Your role is **not** to drive innovations at the site. That is the job of the champion and implementation team at the site.
- Track the progress of your sites, review sites’ mid-point and final reports, and provide updates on site progress at the weekly CTAC internal meeting.
  - [**Tool S: Mid-Point Project Report Template**](#_Tool_S:_Mid-Point)
  - **[Tool T: Final Project Report Template](#_Tool_T:_Final)**
- Convene all-site telephone meetings between the six coached sites (and both coaches) monthly to discuss progress and challenges, and encourage the sites to help each other with quality improvement.
  - *Purpose*: To explore and document cross-site learning.
  - The monthly collaborative calls can be recorded if all participants on the call feel comfortable being recorded.
  - Due to the potentially large number of participants on the monthly call, these calls will need to be carefully planned and very organized.
    - A 1-2 minute project update from each site will help to update participants.
    - A quick didactic exercise may be needed to initiate collaboration. The sites may choose to provide the didactic portion of the meeting (ex. Share what each site is doing; one site per meeting). This should be pre-arranged with each site.
  - ***NOTE***: Make sure to ask all participants to share with the coach when other individuals who are not part of the core site team are invited to the monthly calls. Being aware of potential spread of innovations to non-coached sites will be important to know for evaluation purposes.
- Obtain verbal confirmation from project site members to audio record the weekly conference calls. These recordings will be used to track each site’s progress over the twelve-month project period.

**Events that should trigger the coach to seek help:**

- If a new champion is needed, inform CTAC investigators who will work with leadership to identify a new champion
- Failure to get a response through any means (example: no one joins the coaching call and there is no email response)
  - If some response, follow up twice; If no response, follow up only once before informing CTAC investigators
  - Inform CTAC investigators who will work with leadership to find a solution
- If the site team is insistent on proceeding with a project that is out of scope (example: move the flagpole in front of the clinic so that the location of the clinic is more visible)
  - Time to pause and check in with the CTAC investigator team
  - The local project should map to a care coordination strategy. Davies, et al. (2008) present an analysis of coordination strategies in primary care that may be help guide the scope of the project. <https://www.mja.com.au/system/files/issues/188_08_210408/pow11099_fm.pdf>
- If disruptive behavior (e.g., non-civil discussions) becomes an issue, inform CTAC investigators
- Clinical triggers – Knowing when to bring in clinical help on a topic:
  - Before the site explores the topic in depth, let them know that it may be better for CTAC investigators with clinical expertise to come online. Fill out [**Tool U: Clinical Query Form**](#_Tool_U:_Clinical) and share with CTAC investigators prior to the next project call with the site.

## Site Quality Improvement Team Responsibilities

**The site’s interdisciplinary quality improvement team is responsible for:**

- Participating in a kick-off in-person site visit with the coach and/or other CTAC team members.
- Choosing a quality improvement topic or issue that is important to the clinic (and the leadership of the facility that the clinic is part of) and implementing a project based on that topic. Tool(s) from the Care Coordination Toolkit may be used during this project to improve care coordination in the clinic, or other tools may be found or developed for use during the project period.
- Communicating with the coach on a weekly basis via conference call to report on progress and barriers.
- Participating in a quarterly call with the coach and site leadership.
- Participating in monthly all-site conference calls (with six sites and two coaches) to share experiences and serve as a community of practice.
- Completing the project planning documents as specified by the coach as well as mid-point and final reports based on the schedule outlined by the coach.

**Each team will be led by a champion who will:**

- Serve as point of contact for the coach.
- Lead the coordination of the coach’s site visit.
- Lead and organize the site’s quality improvement team.
- Provide updates during weekly calls with the coach, monthly calls with site leadership, and monthly calls with other sites.
- The champion will also debrief with the coach after each weekly call (if necessary).
- Ensure mid-point and final reports are completed and submitted on time.

# Project Timeline and Steps

## Project Timeline^[[8]](#footnote-8)^ & Gantt Chart


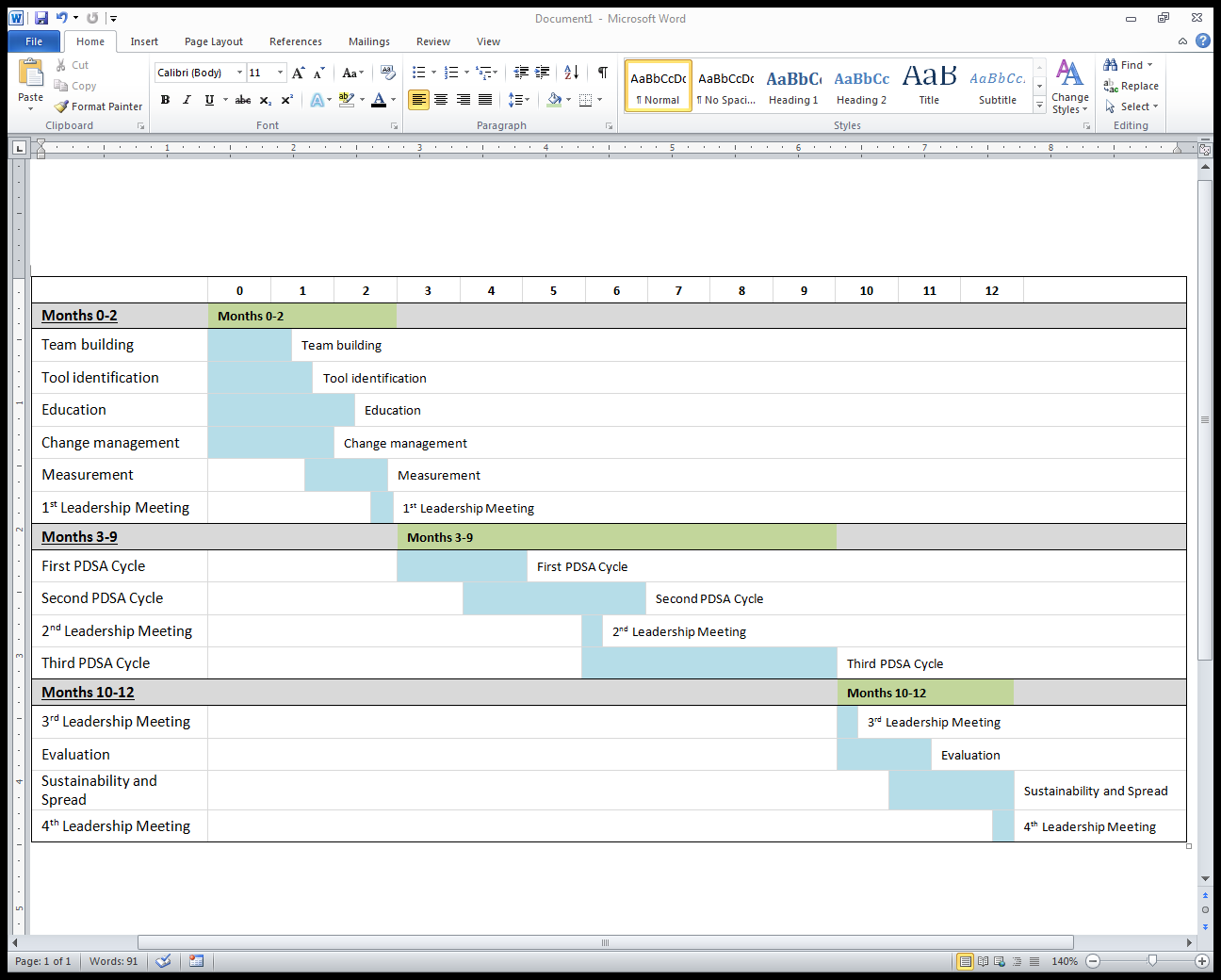


### **MONTHS 0 – 2**

- 1. **Team building:** Assist the champion in building a multi-disciplinary quality improvement team and get to know the team members. This includes introductory phone calls and e-mails and the scheduling of a weekly team meeting.
  2. **Tool identification:** Help the team identify 1+ tool(s) to implement from the Care Coordination Toolkit as part of the team’s quality improvement project. The team may also choose to find or develop additional tools.
  3. **Education:** Educate the team on quality improvement change processes including process mapping, Plan – Do – Study – Act Cycles, and measurement tools.
  4. **Change Management:** Help the team map out a change process and timeline, assign roles, and identify benchmarks. Identify performance gap/problem statement, current state, target state and action plan.
  5. **Measurement:** Help the team choose improvement measures and measurement tools, and collect baseline data.

### **MONTHS 3 – 9**

- 1. **First PDSA:** Coach the team through its first PDSA cycle and help refine the implementation plan.
  2. **Second PDSA:** Coach the team through its second PDSA cycle.
  3. **Third PDSA:** Coach the team through its third PDSA cycle.

### **MONTHS 10 – 12**

- 1. **Evaluation:** Coach the team on how to evaluate the implementation. Compare final state (after implementation) to initial state (before implementation). Reflect on insights and lessons learned.
  2. **Sustainability and Spread:** Coach the team on how to sustain progress and spread innovation to other clinics. Stress that time needs to be set aside to make successful QI projects the new routine.

## Project Steps

### **Team building**

The coach will assist the site in building a multi-disciplinary quality improvement team. An effective team for adopting and implementing a new tool into the clinic will have several characteristics. The coach will make the site aware when other team members may be needed, including team members who can be called on to engage other key individuals.

Use [**Tool V: Team Contact and Information Sheet**](#_Tool_V:_Team)**^[[9]](#footnote-9)^**: A contact and information sheet to fill out for each coached site. The sheet is meant to include information about the project site team as well as the GLA CTAC team and should be shared between teams.

1. **Team members from many areas with the necessary expertise to address the problem*.*** Senior leadership support is a prerequisite for system change, but change itself comes most effectively from the ground up. Change happens when teams that include frontline health care workers actively engage in high-priority problem solving, such as redesigning processes of care. Including clinical staff as members of the team is key to tapping their practical knowledge and engaging them in the change process.
2. [**Tool W: Interdisciplinary Team**](#_Tool_W:_Interdisciplinary)^[[10]](#footnote-10)^: may assist in identifying potential team members
3. Members of an interdisciplinary team could include^[[11]](#footnote-11)^:
   1. Facility Directors
   2. Chiefs of Staff
   3. Associate Directors for Patient Care Services/Nurse Executives
   4. Service Chiefs
   5. Physicians
   6. Physician Assistants
   7. Nurse Practitioners
   8. Registered Nurse Care Managers
   9. Licensed Vocational Nurses (LVN)/Licensed Practical Nurse (LPN)
   10. Health Techs/Medical Assistants
   11. Clerks
   12. Patient representatives
   13. Group Practice Managers
   14. Health Educators
   15. Community Health Workers
   16. Peer Mentors
   17. Patients
   18. Community Representatives
   19. Medical Records Staff
   20. Lab Technicians
   21. Pharmacists
   22. Engineering/Environmental Services
   23. Business Office
   24. Community Care
4. **Strong link to leadership*.*** Some organizations have found that an effective way to obtain senior leadership support for an initiative is to include a senior leader on the team. In this scenario, have the champion think about which senior leader is most appropriate for the implementation team. However, having a senior leader on the implementation team may not be feasible or appropriate in every case. As an alternative, consider having the site champion ask senior leadership to designate a member of the top management team to participate. The team leader should stay in frequent contact with the senior leader and can approach that person when the team encounters obstacles or needs access to senior leadership. In lieu of having a senior leader on the team, leadership may prefer to be kept updated of project progress during monthly leadership calls.
5. **Link to quality improvement expertise*.*** The implementation team will be strengthened by having a member with expertise in systematic process improvement methods and in team facilitation, potentially from a quality improvement or performance improvement department. If the site you are working with does not have a separate department with these functions, consider asking the champion to use informal channels to identify a person with these skills to recruit to the team. In some organizations, a member with improvement expertise successfully co-leads the implementation team with a clinical colleague.
6. [**Tool X: Quality Improvement Process**](#_Tool_X:_Quality)**^[[12]](#footnote-12)^** can help identify quality improvement resources within an organization.
7. **Members who influence the areas that will need to be involved in the project***.* Sometimes it is not possible to anticipate every area that needs to be involved during a project. It is always possible to add team members later, but new members will need to be oriented to the team’s history and process. Therefore, it is best to work with the site champion at the beginning of the project to identify team members that will benefit the chosen project.
8. [**Tool Y: Stakeholder Analysis**](#_Tool_Q:_Stakeholder)**^[[13]](#footnote-13)^** helps identify which departments and individuals will have an interest in the new project. Consider recruiting a representative from each stakeholder group to the team.
9. This Institute for Healthcare Improvement website (Science of Improvement: Forming the Team - <http://www.ihi.org/resources/Pages/HowtoImprove/ScienceofImprovementFormingtheTeam.aspx>) provides both general principles for team composition and several examples of different clinical improvement teams and their membership.
10. **Implementation Team Composition.** Facilities often find it very important that their team be truly interdisciplinary. This composition ensures that as a group, the team can understand quality improvement from multiple perspectives and integrate hands-on knowledge and expertise into the project. Work with the site champion to create an interdisciplinary team that will best fit the project.

### **Tool Identification**

During the site recruitment process, priority setting meeting with leadership, site visit, or at the start of the weekly meetings with the site champion and project team, a care coordination issue will likely emerge as a target for improvement. Once the care coordination issue is agreed upon, as the coach you will guide the site champion and project team in finding a tool(s) that supports and facilitates improvement of the care coordination issue. You can help the team browse the Care Coordination Toolkit to identify tool(s) that will support the chosen project, or if no tool(s) seem relevant you can brainstorm with the team about what kinds of tools would be useful. Ideas for tools that are not contained in the toolkit can either be developed into a tool, or you can assist the site in searching for an already developed tool that may work for the project. Some questions that might be useful when helping a site in their search for tools are:

- What tool(s) are you interested in implementing?
- Why this/these tool(s)? List the characteristics that are most appealing about this/these tool(s).
- Does this tool need to be changed to fit your clinic? How so?

### **Educate the team on quality improvement change processes**

Improvement efforts tend to be most successful when teams follow a systematic approach to analysis and implementation, and there are multiple approaches to consider. Team leaders and members may want to consult more general resources for approaches to quality improvement projects, such as information on the Plan, Do, Study, Act (PDSA) approach - see [**Tool Z: PDSA Worksheet for Testing Change**](#_Tool_S:_PDSA).

1. **Plan – Do – Study – Act (PDSA) Cycle^[[14]](#footnote-14)^:** PDSA is an iterative process based on the scientific method in which it is assumed that not all information or factors are known at the outset; thus, repeated cycles of change and evaluation will be needed to achieve the goal, each cycle closer to reaching the goal than the previous one. With the improved knowledge, you may choose to refine or alter specific goals. Here are the steps of the process:

- **Plan =** Describe who, what, where, when, why and how you will measure the impact of your change test.
- **Do =** Execute a small test of implementation. If the tool is patient-related, it could be tested with one patient.
- **Study =** Evaluate the results based on the metric you chose in the “plan” phase – in what ways was the test successful? In what ways was it not successful? Were there any unintended positive or negative consequences?
- **Act =** Decide what your next cycle of change will be based on the results from the “study” phase.

For more information, refer to Chapter 5 in the RAND report *Putting Practice Guidelines to Work in the Department of Defense Medical System. A Guide for Action*, available at <http://www.rand.org/content/dam/rand/pubs/monograph_reports/2007/MR1267.pdf>.

The Institute for Healthcare Improvement website includes a summary of the PDSA cycle and a clinical example of it in use: <http://www.ihi.org/resources/Pages/HowtoImprove/ScienceofImprovementTestingChanges.aspx>.

If the site you are working with already has well-established quality improvement processes and structures, it will be beneficial to connect the quality improvement project with those processes. For example, if the site has an established reporting structure to leadership, including this project will help keep it on the leadership agenda. If managers are already evaluated based on their quality improvement efforts and results, making this project a part of the larger quality improvement enterprise at the site will help ensure managers’ interest.

You can assess the site’s current resources for quality improvement by asking the champion to complete the quality improvement process inventory found in the [**Management Tools**](#_Management_Tools) section.

- [**Tool X: Quality Improvement Process**](#_Tool_P:_Quality)**^[[15]](#footnote-15)^**

For information on other QI methods, please refer to the QUERI QI Methods website: <http://www.queri.research.va.gov/implementation/quality_improvement/all_methods.cfm>

1. **Integrating Change into Current Work Routines:** Beyond understanding the nature of the problem to be addressed, the team should consider how the care coordination tool can be integrated into current workflow and processes, rather than layered on top of them. One way to approach this task is to systematically assess the barriers to using these practices. The coach can help the team think through how to integrate the tool by using various diagrams:
   1. An ***affinity diagram*** can help organize these ideas. An affinity diagram is used to organize a comprehensive list of ideas about a problem or topic by grouping together similar thoughts. It is especially helpful during group brainstorming and discussions, when diverse and perhaps disjointed information may be shared. Examples are available at <http://www.queri.research.va.gov/implementation/quality_improvement/methods.cfm?method=2>
   2. A ***fishbone diagram*** is way to potentially organize findings from the ***affinity diagram***. A fishbone diagram adds additional structure and detail compared to an affinity diagram and can also help clarify how to integrate the care coordination tool into existing practices. The example below depicts the various contributors to poor communication between PCPs and mental health providers^[[16]](#footnote-16)^:


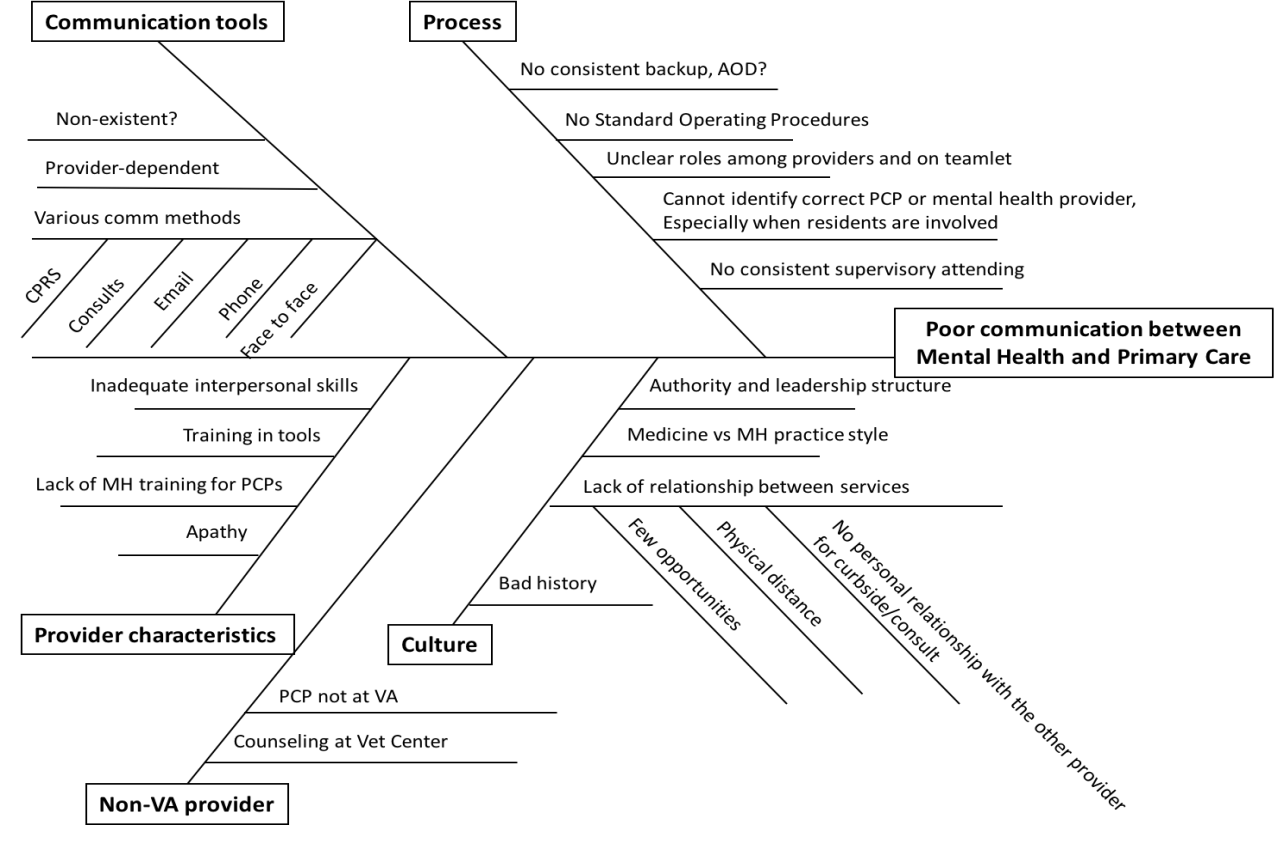


1. **Process Mapping to Document Current Practices:** One useful approach to understanding current practices is to use **process mapping** to examine key processes where activities could or should be happening. Mapping can specify which organizational unit or person carries out each step in the process. The goal of process mapping is to come to a common understanding of how a specific care process is being carried out, which then leads to further discussion about how the process *should* be carried out. There are different approaches to process mapping, but each approach provides a systematic way to **examine each step in the delivery of a specific procedure or service**. Experimentation with different approaches can be helpful during the redesign planning phase because each approach can provide different insights and answer different questions.
2. Detailed instructions on process mapping may be found in [**Tool AA: Current Process Analysis**](#_Tool_AA:_Current)^[[17]](#footnote-17)^.
3. If you would like to learn more about process mapping, the American Society for Quality’s (ASQ) website provides information on how to prepare a flowchart: <http://asq.org/learn-about-quality/process-analysis-tools/overview/flowchart.html>.

For a site to make an informed decision about what changes to make to its practice or process, the site needs to see the big picture of its current system, everyone's role in the patient’s care, and the common problems it is facing. First, the coach should work with the team to create a diagram of the ***current* process**, **NOT** how they think the process *should* be. Using the Microsoft Visio program, walk the sites through creating a current process map. Sites may need to have the Microsoft Visio program installed on one of their local computers. If site teams need a tutorial on using Visio, you can provide the following YouTube link: <https://www.youtube.com/playlist?list=PLTtplW6mJ7fWSIvHScqHCkaA4T0HhaWU->.

During a conference call the coach can share his/her screen with the site and create the Visio map while the team talks through the process, or, if the site has someone who knows how to use Visio, the site can create the Visio flow diagram and share screens with you. After creating the current process map, the team can use this information to identify where the process needs to change in order to implement the new process, tool or procedure. A second process map can be created later, as the project progresses, in order to illustrate how the clinic process has changed.

The end-product should be a process map such as the one below. This example depicts the process by which psychiatrists contact primary care providers (PCPs) in the emergency room.^[[18]](#footnote-18)^


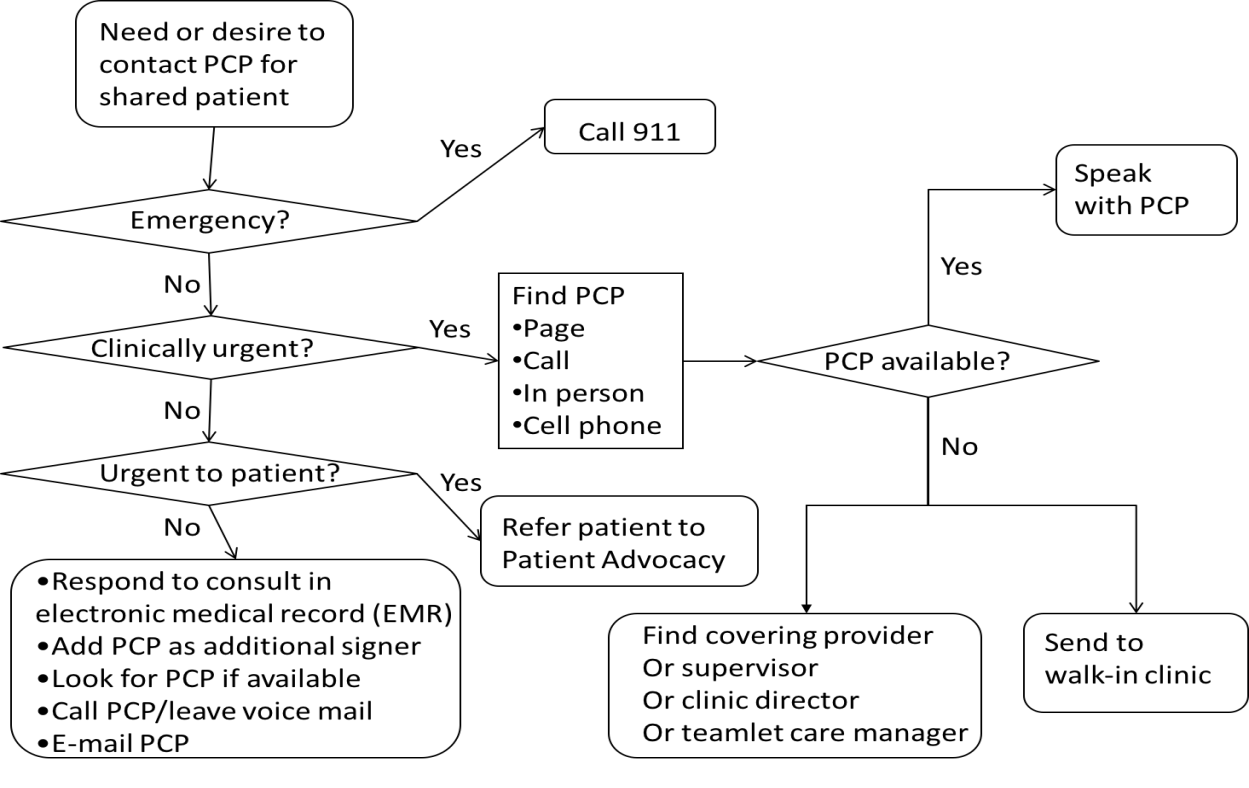


The QI Methods Toolkit can provide additional resources and examples and can be accessed at <http://www.queri.research.va.gov/implementation/quality_improvement/default.cfm>

### **Change Management**^[[19]](#footnote-19)^

Help the team map out a change process and timeline, assign roles, and identify benchmarks.

#### **How can you help the implementation team start its work?**

Changing routine processes and procedures to alter the ways people conduct their everyday work is a major challenge. Successful implementation teams—teams that achieve their goals and sustain improved performance have the following characteristics:

- They pay attention to the development of routines that make the new practices better than existing practices.
- They identify and implement new practices that are easier, more reproducible (not reliant on memory), and more efficient than old practices.

The implementation team itself needs structure to achieve its objectives. Items to settle on early include:

- How often the team should meet (e.g., weekly).
- Ground rules or guidelines for how to manage meeting time and for how to communicate, both internally and externally.
- Timeline for the team‘s work so that there is a shared understanding of the level of urgency and priority this effort requires.

To assist in these objectives, refer to the [**Tips for Facilitating Effective Meetings section**](#_Tips_for_Facilitating_2)**^[[20]](#footnote-20)^**

When helping a team with change management, consider the following questions:

1. **How will the team do its work?**

This question refers both to the resources the team may need (information, materials) and to methods of working. How will the team track issues raised, explored, and addressed? How will the team assess current knowledge and practice? How will the team use that information to redesign practice?

- [**Tool BB: Assessing Staff Education and Training**](#_Tool_BB:_Assessing)**^[[21]](#footnote-21)^** can help assess current staff education practices and facilitate the integration of new knowledge into existing or new practices.

1. **What is the team’s agenda?**

This question emphasizes the importance of giving the team a clear charge and scope for its work. Team members will need a clear understanding of the short- and long-term goals and timeframes for the implementation of improved care coordination practices. Leadership may provide the team with a written charge that specifies target dates and improvement goals or this may be something you help the team establish. [**Tool P: Quality Improvement Project Action Plan Template and Sample**](#_Tool_H:_Quality)**^[[22]](#footnote-22)^** will help the team think through the steps and timeline for the project. The Action Plan Template should be completed near the beginning of the project to set goals and a timeframe for the project tasks. See [**Tool M: Sample Weekly Timeline and Plan (Weeks 1-8)**](#_Tool_M:_Sample) for guidance on when this document should be introduced and completed.

Tools that may help the change management efforts include**^[[23]](#footnote-23)^**:

- [**Tool P: Quality Improvement Project Action Plan Template and Sample**](#_Tool_H:_Quality): An example of an action plan to design and implement a QI project.
- [**Tool CC: Managing Change Checklist**](#_Tool_CC:_Managing) and [**Tool DD: Implementation Checklist**](#_Tool_DD._Implementation) help monitor progress on managing and completing change activities.
- [**Tool EE: Assigning Responsibilities**](#_Tool_EE._Assigning) and [**Tool FF: Staff Roles**](#_Tool_FF._Staff) can help identify who will be responsible for each task.
- The Institute for Healthcare Improvement Web site (Science of Improvement: Setting Aims - <http://www.ihi.org/resources/Pages/HowtoImprove/ScienceofImprovementSettingAims.aspx>) has guidance on setting team goals and other aspects of team startup.

#### **How to get clinic staff engaged and excited about coordination?**

Obtaining the buy-in and participation of clinic staff members is particularly important for those involved in hands-on care and whose involvement will be needed to achieve the improvement project objectives.

In preparation for the project, the champion and implementation team should meet with clinic staff and review the scope of the project and any newly defined roles or responsibilities. The champion and implementation team should work together with the clinic staff to determine how to adjust roles and paths for communication and reporting as well as discuss how to address barriers to adherence.

Some clinicians and staff may be reluctant to use a new set of practices or change clinic procedures. If reluctance or active resistance is localized to specific individuals, you may decide not to include those individuals in the project at the outset. The implementation team can instead focus on the people with the greatest interest and highest likelihood of success. If resistance during early implementation is widespread, it will be important to understand why. If necessary, the implementation strategy or set of practices may need to be redesigned. The coach should use discretion in these situations and discuss any issues privately with the clinic champion. If the clinic champion is too passive, leadership may need to be brought in.

### **Measurement**^[[24]](#footnote-24)^

Help the team choose improvement measures and measurement tools, and collect baseline data.

- - What measures will you use to evaluate whether the change you hope for is occurring?
  - How will we know if a change is an improvement?
  - Measurement plans may change throughout the course of the project. Perhaps a measurement plan is too challenging to implement and needs to be refined or discarded.
  - Make sure to check in with teams not only on their implementation progress, but also on their measurement activities.

**Definitions:**

- **Process measures:** Something that you can measure quickly (in 1 week) that shows that your change is happening. Example: the number of patients who were given an after-visit summary divided by the number of patients seen in clinic over 1 week.
- **Balancing measures:** What should you measure to make sure that you are not accidentally making something worse? Example: the average time (in minutes) beyond their scheduled appointment time patients wait to be seen; measured before and after doing the after-visit summary.
- **Outcome measures:** What is the final patient outcome you hope to change in the long term? Example: Percent of patients whose experience of care coordination is improved compared to baseline.

**Tips for constructing useful measures:**

- 1. What is the population in which you expect to make the change? This will be the denominator for your measurement. Example: Patients who come in for primary care visits with the Gold Team.
  2. What is the definition of progress towards your goal? This will be the numerator for your measurement. Example: Number of patients who receive an after-visit summary at the completion of their visit.
  3. What is the source of the measure? Example: Number of patients who come in for primary care visits will be obtained through administrative data and the number of after-visit summaries given will be collected by the clerk via a tally sheet.
  4. How will you collect this data? By observation, surveys or chart review?
  5. Do you need additional members of your team to help collect the data? Example: Clinic clerks need to fill out tally sheets.
  6. How often and for how long will you need to collect this data? Be sure that you are looking at a typical one or two weeks—not a time around a major holiday, when key staff is on vacation, when the clinic is unexpectedly understaffed, or during flu season.
  7. Once you know what data you want to collect, what are your baseline results? Discuss with your team. Is this what you expected? If not, does this impact the change you planned to make? Example: The first day of the project, the clerks returned blank tally sheets – no tallies were made.

**Brainstorm Improvement Measures**

| **Measure** | **What type of measure is this?**  **(process, balancing, or outcome)** | **Expected benefit of change or impact** |
| --- | --- | --- |
| Team Member X to contact 20 patients after a month of implementing the After-Visit Summary and ask them whether they received the summary – and if it was helpful to them? | Process measure | Most patients will have received the summary and found it to be helpful. Example: At least 80% of patients received the summary. Of those who received the summary at least 75% were completely satisfied (“5” on a 1-5 scale) with the summary. |
| Team Member X to stamp arrival time of patients and compare to scheduled appointment time for 20 patients before and 20 patients after starting the after-visit summary. | Balancing measure | Arrival time relative to appointment time showed no major change (no more than a 5-minute delay between before and after implementation of the after-visit summary). |
| Team Member X to access the clinic’s SHEP data on the VA intranet 1 month before and 3 months after the implementation of the after-visit summary. | Outcome measure | The clinic’s performance on Survey of Healthcare Experiences of Patients (SHEP) measures related to care coordination improved to the 90^th^ percentile nationally. |

Below are some examples of data collection tools. Based on what measure the implementation team would like to use, help the team choose the best data collection tool.

- 1. **Tally Sheet** – A tally sheet can be used to keep track of how often something is taking place in the clinic. A tally can be done prior to implementation of a new procedure or tool and then again after introduction of the tool or procedure. A comparison can be done on pre- and post-totals. The example below shows number of clinic visits each day and how many after visit summaries are provided to patients each day.

| **WEEK** | **# of Clinic Visits** | **# of After Visit Summaries** |
| --- | --- | --- |
| Day 1 | ***/////*** | *////* |
| Day 2 | ***/////*** | *////* |
| Day 3 | ***/////*** | *////* |
| Day 4 | */////* | *////* |

- 1. **Pareto Chart** - The purpose of a Pareto chart is to show the common reasons for a specific issue or problem happening in the clinic. The implementation team can brainstorm common causes of a specific problem or issue in the clinic, with regards to the chosen topic. These ideas are input into a table and tracked over a short period of time. The data collected can then be used to create the Pareto chart or graph. The example below shows a table and then a Pareto chart for the most common reasons an after-visit summary is not provided to a patient at the end of the visit.

|  | **Printer Did Not Work** | **Doctor Did Not Fill out the Summary Before Patient Left** | **Clerk Did Not Hand the Summary to the Patient** |
| --- | --- | --- | --- |
| Number of events in 2 weeks | 13 | 11 | 7 |

The data gathered in the table can be displayed visually in a Pareto chart (see below). The bars indicate the contribution each factor makes to the problem, while the line graph visualizes the cumulative contribution of all factors. Results are typically rank-ordered from the most common to the least common causes of the problem.

### **Plan-Do-Study-Act (PDSA) cycle^[[25]](#footnote-25)^**

The Plan-Do-Study-Act (PDSA) cycle can be used to test changes in real settings. The PDSA cycle guides the test of a change to determine if the change is an improvement. When using a PDSA cycle to test the effect of a tool it is best to scale the implementation of a tool. Try implementing the tool on a small scale, for example, with one patient before choosing to implement it at the clinic level. The below list of questions can be used as a resource for guiding teams through their PDSA cycles. Other resources include:

- [**Tool CC: Managing Change Checklist**](#_Tool_V:_Managing)^[[26]](#footnote-26)^: A checklist for monitoring the managing change activities.
- The Institute for Healthcare Improvement’s PDSA worksheet is useful for documenting a test of change. Fill out one PDSA worksheet for each test you conduct. Each change will go through several PDSA cycles. <http://www.ihi.org/resources/Pages/HowtoImprove/ScienceofImprovementTipsforTestingChanges.aspx>

**PLAN: Review your plan to carry out your first intervention cycle with everyone who will be touched by the change you are planning.**

Consider preparing a flow map of how the changed process will look. Discuss the following:

- What is the change you plan to make? (e.g., every patient will receive an after-visit summary after his/her primary care visit)
- Where will the change take place? (e.g., in the clinic at the checkout station and/or in the provider’s office)
- Who will carry out the different parts of the change? (e.g., the clerk will hand out the after-visit summary to the patient. The provider will...)
  - Who will implement the change?
  - Who will collect the data?
  - Who will analyze the data?
  - Do you require extra resources? E.g., Clinical Application Coordinators to program changes into CPRS? Who will be responsible for contacting them and acquiring their services?
  - How will you disseminate your plans for the intervention to all those who need to be aware?
  - Is there additional data that you will need to collect that is specific to your planned change? (e.g., Number of after-visit summaries handed out by the clerk compared to the number printed by the provider)
  - What is your proposed timeline?
- What date will you implement the change?
- When will you start to collect data?
- How long will the data be collected?
- When will the data be analyzed?
- What barriers do you anticipate that may keep your intervention from being successful?
- Is everyone involved clear about their role, when to begin and end baseline data collection, and who to report to if any problems or questions develop?
- Have you built in the task and time to review the first few days following your change start date to be sure everything is going as planned?
- Might there be unintended consequences of the change that you also need to measure?

**DO: Strategy for Change: Describe what is happening during your test of change.**

- Are you able to implement your change as planned?
- Are there any problems with communication within your team? If yes, what are they? How can they be prevented moving forward?
- Has anyone run into problems in carrying out their role in the change? How can you work around this to get back on track?
- Are there any unexpected delays or unforeseen barriers to your change? If yes, what are they and how will you compensate for them?
- How is the team functioning during the change?
- Are there any other staff members participating in your intervention who were not on the planning team? If so, who are they? Do you need to add them to the team?

**STUDY: Effects of the test of change. Analyze and interpret your data.**

- How do you and your team feel about the change that was implemented?
- Were there unintended consequences resulting from the change? (These would be considered balancing measures.) If yes, what were they and can/did you measure them?
- What data do you have now? Is it complete enough and typical enough to have confidence that this is a valid description of the impact of your change? Do you need to collect any additional data to really know what is happening?
- Looking back at your baseline data, how close did this first cycle of change get you to your aim?

**ACT: Lessons Learned—How to improve or sustain and disseminate the change**

- - Was the change an improvement? Yes or No?
- If No, what is your next step? Do you need to further refine the process, add something more, conduct a second cycle of change?
- If Yes, can you spread the change beyond your initial area of implementation? How will you plan to sustain the changes made? Are they sustainable?
- What did you learn from the test of change?
- What aspects of the change worked well?
- What aspects of the change were problematic?
- Are there any other types of measurement that may help you understand the problem better?

If you have not yet achieved your AIM, should you implement a second cycle of change? Most projects have some initial success after a first round of implementation, but additional ways of continuing to improve the process are often suggested by the first trial. For that reason, we encourage a second cycle of change.

### **Sustainability and Spread^[[27]](#footnote-27)^**

Coach the team on how to sustain progress and spread innovation to other clinics. Coaching on sustainability and spread will happen in months 10-12 of the project. After the 12-month project period has ended the champion and/or clinic sustainability team will be responsible for maintaining the innovation at the clinic and/or spreading it to other clinics or sites.

**How is an effective innovation sustained?**

To sustain improvement, changes need to become integrated into existing organizational structures and routines so that they are no longer noticed as separate from business as usual. While sustaining changes logically follows initial improvements, it is important to begin thinking early in the improvement process about what will be needed to make lasting change. It is important to ensure that any changes being implemented are not dependent solely on initial enthusiasm. Throughout the implementation process, you should consider questions such as:

- Who will be responsible for sustaining care coordination efforts on an ongoing basis?
- [How will the clinic continue to monitor the quality of care coordination activities after the project period?](#_bookmark59)
- [What types of ongoing organizational support does the clinic need to keep the new practices in](#_bookmark60) p[lace?](#_bookmark60)
- [How can the desired results be reinforced??](#_bookmark61)

Two tools that can help identify sustainability issues in planning and implementing the project are [**Tool GG: Sustainability Tool**](#_Tool_GG._Sustainability)^[[28]](#footnote-28)^ and [**Tool R: A3 Worksheet**](#_Tool_K:_A3)^[[29]](#footnote-29)^.

1. **What types of ongoing organizational support are needed to keep an innovation in place?**

Sustainability activities can include:

- Training for new employees and refresher training for current employees
- Prompt filling of staff vacancies by human resources
- Prompt provision of needed supplies and equipment by facility management
- Help from information technology staff to assist with regularly reporting data

The sustainability team will need to consider strategies to keep the clinic champions engaged and a method to replace the clinic champion when he/she changes responsibilities or positions. Similarly, the clinic sustainability team will need processes for keeping other staff engaged and replacing them when needed.

Communication is essential to keep staff involved and up to date. Consider suggesting ongoing information briefs in the clinic’s staff bulletin. Posters can also be used; rotating them every few weeks may be important in keeping staff engaged.

1. **How can the desired results be reinforced?**

Generating and maintaining excitement about change is critical to success. Given the obstacles to implementation, improvements in performance measures may not initially be evident. It is thus important to find small successes early on that can be rewarded.

- The coach can help the site identify short- and long-term outcomes to celebrate.
- Examples: providing certificates of appreciation to individuals contributing to make the program a success; ongoing information briefs in the staff bulletin; make the QI topic a standard part of yearly staff education fairs or similar events, etc.

1. **How to handle staff turnover while sustaining an innovation.** To address the barrier of staff turnover, ensure that orientation for new clinical staff is modified to include a focus on care coordination and that new staff are appropriately integrated into the clinic’s program. This will help to maintain a clinic culture that is positively oriented toward care coordination and to the innovation.

- Sustainability products such as an introductory letter to familiarize new staff with the QI project or process can be produced as a project deliverable.

1. To continue to reinforce desired results and sustain the program, be aware of the following obstacles that can occur:

- Old habits have a way of resurfacing. People may slowly go back to old approaches. This tendency supports the need for ongoing refresher training in the context of each clinic‘s needs.
- Practices that had become accepted may suddenly be more difficult to perform or the availability of needed resources may change. Such unintended consequences of quality improvement are well recognized.
- The coach should remember that the goal is to institutionalize what works (e.g., if the QI project is successful) so that it is sustained (e.g., national mandate, VISN approval).

# Project Evaluation

The coach will help the site develop measurement and data collection processes to evaluate the QI project. The coach needs to stress the importance of continued data collection for evaluation purposes beyond the scope of the CTAC project. The data collection and evaluation processes that will be modeled during CTAC should be used and updated continuously to ensure the sustainability of the QI project past the project period.

### **Ongoing Evaluation (Informal & Coach Directed)**

The coach will help the site identify components of the project that worked, components that could be improved, and components that failed and should be eliminated. Identifying these components will help determine which aspects of the QI project should be sustained. The coach should encourage the QI team to reflect on the implementation and evaluation processes.

### **Mid-Point and Final Reports**

Sites should use [**Tool S: Mid-Point Project Report Template**](#_Tool_L:_Mid-Point) and [**Tool T: Final Report Template**](#_Tool_M:_Final) to track their progress. The Mid-Point and Final Reports help sites:

- Outline their project’s purpose and goals;
- Describe the measures used to track project outcomes; and
- Identify early findings and needed modifications to achieve project goals (Mid-point)
- Identify key findings and results using tables and figures when appropriate
- Discuss implications and recommendations for sustaining successful processes

# Tips for Facilitating Effective Meetings^[[30]](#footnote-30)^

### **Tips for Facilitators**

These are general facilitation tips that may be useful when coaching implementation teams. The below information has been adapted from materials prepared by Aspire Consulting for the VA Team Development Measure (TDM) website: <http://www.queri.research.va.gov/tools/tdm.cfm>

1. Focus primarily on the process of the meeting and not the content. Generally, the team will provide the content based on the project and their own approach. Open the meeting with a summary of the previous meeting when appropriate. Recognize success and progress made.
2. Always remain neutral and avoid adding your personal ideas to the team discussion. Remember, their involvement is the most important since they are the ones working together on a routine basis. This experience is about them and not about you. Think of yourself as an enabling force to help the team get results.
3. Protect the team against personal attacks on ideas and philosophies. Encourage productive discussions that value all ideas and suggestions. Set some meeting ground rules that include “All ideas and questions are welcomed.”
4. Maintain a balanced focus on both positive (successes or strengths) and negative (challenges or areas of disagreement).
5. Ensure that everyone has an opportunity to participate and provide input. Everyone is different so expect a mixed level of participation. However, be aware of those who say nothing or those who want to contribute and are not being heard.
6. Clarify and summarize what has been said throughout the meeting. Phrases like, “Let’s summarize what we’ve accomplished so far…” or, “It sounds like everyone agrees on X, so let’s move on and discuss Y.”
7. Use questions and the agenda to keep the group on track. “Are we all in agreement on that issue?” or, “What does everyone think about that result? or, “Is everyone ready to move on?”
8. Close the meeting by recognizing progress and participation. Summarize all decisions made throughout the meeting. Be sure everyone agrees on actions and accountability associated with the team plan.
9. Be sure to schedule a follow-up meeting with the team. It is essential that they have a “next step” and a time set to measure progress against their action plan.

### **Language to Support Structure**

In addition to the agenda, your choice of words has the power to encourage team progress and keep things moving. Use these discussion skills to help facilitate team discussion:

1. Structure Statements: Set expectations and establish goals and structure to the conversation by sharing an overview of the planned topics. For example: “Our plan is to discuss A, B and then C...”
2. Validate Questions: Reconfirm that you understand what each person said and meant. For example: “If I understand you correctly, you are saying...” or, “Just to be sure everyone is on the same page, are you saying...”
3. Clarifying Questions/Statements: Acknowledge your limitations and encourage clarity. For example: “Since I do not have a clinical background, what do you mean by…” or “Since this may be different in your VISN/clinic/etc. from what I am familiar with, could you explain what you mean by…?”
4. Summary Statements: Identify what was accomplished at key points in the conversation. For example: “Thanks for a great discussion on that. Now we’ve covered XYZ, accomplishing our goal for that topic today.”
5. Transition Statements: Indicate that the current conversation is finished and close on a positive note before moving on. These statements often follow a summary statement. For example: “Let’s move on to talk about XYZ as our next topic on the agenda today.”
6. Statements of Closure: Indicate that the meeting is finished, highlight progress and results, and close on a positive note. For example: “Thanks for your active participation and great progress...” or, “Our next steps include...”

### **The 4 Ps**

Another approach for facilitating successful meetings is to structure meeting content using the 4 P’s: Purpose, People, Process and Progress.

1. **Purpose**—Begin with the end in mind by opening and closing each meeting with a main purpose. The purpose often includes a list of goals that define success. For example, the purpose of a meeting might be to:

- Connect as a team to choose a tool/procedure to implement
- Discuss results and highlight successes and opportunities
- Pinpoint priorities for action by assessing areas with the greatest differences in perspective
- Agree on realistic action steps and build a plan for success

1. **People**—Reinforce the importance of team and the idea that involvement leads to commitment.

- Encourage the team to identify primary team members or members of the core team. Who are our primary team members involved in every meeting? Which people are rotating team members or individuals that have secondary levels of involvement on an “as needed” basis?

1. **Process**—Highlight the process for accomplishing the goals of the meeting using the agenda as a primary tool.

- “Our process for accomplishing our meeting goals is driven by our agenda. Let’s review it in detail before we get started.”

1. **Progress**—Set an expectation for how progress will be measured for today’s meeting, and be sure that you reinforce how progress will be measured for action steps that are ongoing.
   - - “At the end of today’s meeting our success will be measured by...”
     - “What small tests of change will you apply between now and the next meeting? How will you measure your success?”
     - “What will you do to be sure the team stays committed to the action plan and how will you measure your results?”

### **Interactivity**

How you start discussions, keep them going, and close them is very important to the learning experience.

**Starting and keeping the discussion going**

- Be upbeat and positive to get people excited about the meeting.
- Emphasize the importance of sharing ideas by setting the expectation that the success of this meeting relies on active team discussion.
- Set a goal of getting at least three comments from the team when you lead a discussion by asking a question. Feel free to tell them that this is your goal so they know you will wait until you get some input.
- Establish “ground rules” for discussion and encourage the ongoing use of them to support team interactions. This is especially true if there is a lot of energy in the group and it’s hard to make progress due to lots of talking. Here are some ideas for ground rules:
  - Everyone listen respectfully to each other (even if they disagree).
  - The person who is speaking should not be interrupted.
  - No more than one person should speak at the same time.
  - All ideas are welcome.
  - If you disagree with someone, disagree with their ideas, but don't attack the person.
  - Try to understand others as much as you hope they try to understand you.
  - Assume positive intent.
- Ask open-ended questions to start the discussion and engage the group. Keep it simple with broad questions for discussion such as:
  - Does your clinic have any care coordination success stories? What are they?
  - What are the biggest barriers to care coordination?
- Keep the discussion going - Your job during the heart of the discussion is to keep things moving, keep the atmosphere respectful, and keep the group "on topic” in relation to the agenda.
- Encourage participation by thanking the team and recognizing key contributions. Say things like: “Great discussion” or “Thanks for the great teamwork.” This encouragement can be shown through words, positive facial expressions (i.e., smiling and nodding) and body language (i.e., facing the person talking and making eye contact). For phone interactions, positive encouragement can also be shown via e-mail after the call (i.e., sending someone a private email to thank them for a good contribution).
- Use probing questions to expand discussion when appropriate.
  - “What do you think might account for the variation in team members’ responses?”
  - “What are the things that we think are most important to work on?”
- Be patient and wait for responses if there is silence after you ask a question. Keep in mind that people need time to think. Wait at least 30 seconds before repeating or changing your question. This gives people time to think and shows them that you care more about their learning than about the speed of their responses.
- During the meeting, as possible, keep detailed notes to track group progress. [**Tool N: Meeting Call Log**](#_Tool_N._Meeting) can be a great tool to summarize the discussion and results of team meetings. The Call Log can be updated with more detail post-meeting and used to update meeting attendance at each meeting. You may also want to send meeting highlights and a discussion summary via e-mail after the meeting.

**Closing the Discussion**

It’s always a good idea to highlight the positive aspects of discussions and thank the team for their involvement. Guidelines for closing a discussion include:

- Summarize results by highlighting what the group accomplished and recapping agreements and action steps.
- Revisit unresolved issues or “Parking Lot” items and ask the team to make a commitment regarding action and timing for each one.
- Ask the group for feedback. Check in with the team by asking them for their thoughts about how the meeting went.
  - What went well?
  - What was difficult, and why?
  - What practices will you adopt for future meetings?
- Thank the group for their active participation. Be sure to let people know that when they add something to the discussion it is more valuable for everyone.

### **Handling Common Challenges**

Whenever we bring groups of people together for meetings, it’s normal to experience some challenges. Here is a list of common challenges and recommendations to overcome them.

| **Challenge** | **Recommendations** |
| --- | --- |
| Silence | - Wait 15 Seconds – people will respond after a short period of silence (15 seconds feels a lot longer than it sounds). - Say “I’d like to get one idea from each of you.” This sets a clear and fair expectation that everyone contributes. - Explain that this is a time for discussion, and input is important. This sends a message that everyone’s thoughts are valued. - Share that this is a collaborative meeting and input is essential. Stress that the QI project is for the site and by the site. Encourage ownership of the project. |
| Out of Control Discussion | - Recognize the group – “Great discussion everyone. Let’s regroup and summarize where we are.” - Use a transition statement – “We have great energy today. Let’s move on to our next topic.” - Stick to the agenda – “This is important and we have a lot to say about it, but it’s time to move on for now. Let’s post it on the ‘Parking Lot’ so we can come back to it at an appropriate time.” |
| High Levels of Emotion | - Acknowledge the emotion – “I can tell that this is important to you.” - Set a time limit – “I can see that this is a hot topic for all of us. Let’s take 5 minutes to process it, and if it’s not something we can resolve today we’ll come back to it at another time.” |
| Rigid or Inappropriate Responses | - Open the mind – “That’s one idea. Have you considered it in a different way? What other angles could you use to view it?” - Acknowledge all comments – “I’m glad you said something. I want to be sure that we’re all on the same page. When you say that, do we understand correctly that you mean…?” |

### **Coaching Fatigue**

Coaching that requires a long-term commitment and/or frequent interactions with the coached team can be emotionally and mentally fatiguing. Although coaching with negative outcomes can be draining, coaching that results in positive outcomes can also be emotionally and mentally taxing. Below are common causes that generate fatigue and recommendations to address them.

When possible, plan for the coach to have structured debrief sessions, especially after difficult coaching sessions. The debrief can happen with other coaches or program staff. It helps to debrief with someone who is familiar with the coaching and coached site.

| **Cause*** | **Recommendation** |
| --- | --- |
| Lack of progress/follow-through | - If an individual, reach out privately and ask if you can provide any support. - Reach out to champions/team leaders for help identifying the cause of the lack of progress. - Ask individual/group if the task in question should be reassigned to a different team member or if other team members can help support. - Re-evaluate project tasks with the group to assess whether they need to be changed or made more manageable. |
| Changes to core team (e.g., turnover) | - If advance notice is provided, identify who else on the team has similar skills/interests and can take over the role of the team member leaving. - Ask the departing team member to explain their process to the remaining group so that activities can continue. - If the champion/team leader is leaving, ask them for help identifying who on the team or in the clinic might be a good replacement. |
| Emotion/frustration directed at coach | - Keep calm and remain professional. - Assess your own emotions and determine if you are okay to continue with the call.   - If YES: “I can tell that parts of this process are frustrating. Why don’t we take a minute to figure out what we can all do to make it less frustrating…?”   - If NO: “I feel we’ve reached a point where we all need a break. Let’s all take some time to regroup and we can talk again (e.g., next week, in an hour, etc.).” - Reach out to team members individually via phone or message to make sure everything is okay with them. If necessary, outline the best ways they can privately communicate any frustration with you (e.g., not during the coaching session). |
| Mismatched expectations vis-à-vis coach | - Hold a meeting to review the project goals and to re-evaluate coaching and team-member expectations of the project and of each other. - Explain your role as the coach and the tasks you are able and unable to perform in support of the team. |
| Timeline and deliverables | - Create a plan for yourself with markers for when deliverables are due and how much time is left in the project timeline. - Expect activities to take longer than planned. - Identify the minimum work you/the project is willing to accept from the team being coached. |
| QI methods and data collection | - Engage early in discussions about the why and how of QI methods and data collection to establish buy-in and to highlight their value. - QI methods and data collection may require additional education or reframing of the concepts for staff to see value. Tie the data collection tasks to a bigger goal such as a report to leadership. |
| Managing team dynamics | - Distance-based coaching makes it difficult to visually assess team dynamics through body language and other social cues. Tone and silence can be good cues, but recognize that understanding how individual team members feel about each other may be more difficult to judge virtually. - The coach can be a good sounding board or mediator between team members in conflict. Offer to be the go-between to find resolution. |
| Communication | - Set clear parameters for how (e.g., phone, email, instant messaging) and how often communication between the team and the coach will happen. - Understand that communication between the team will happen outside of these set times. That communication may or may not be shared with you but will definitely impact team dynamics. Be flexible and process new information quickly. |

* The size of the group being coached can impact fatigue with larger sites often more difficult to manage.

# Tips for Effective Virtual Facilitation

The CTAC coach will primarily support the QI teams through virtual facilitation. The below information about effective virtual facilitation was borrowed with minor adaptations from the *Implementation Facilitation Training Manual: Using Implementation Facilitation to Improve Care in the Veterans Health Administration (Version 2).* The manual was developed by the Implementation Facilitation Training Manual Revisions Workgroups, chaired by Mona J. Ritchie, PhD, MSW of the QUERI for Team-based Behavioral Health *-* [*https://www.queri.research.va.gov/tools/implementation/Facilitation-Manual.pdf*](https://www.queri.research.va.gov/tools/implementation/Facilitation-Manual.pdf)

A. Advantages **and Disadvantages of Virtual Facilitation**

**Interpersonal Connections**

- Networking and interpersonal contact may be stifled (disadvantage)
- Improves likelihood of attendance and expands pool of likely participants (advantage)

**Engagement**

- Participants may become inattentive or absent without face-to-face proximity (disadvantage)
- Allows off-line work to continue (advantage)

**Documentation**

- Risk of over-monitoring or a culture of surveillance (disadvantage)
- Facilitates tracking and archiving of work-activities (advantage)

### **Additional Advice for Effective Virtual Implementation**

**Recommendations for Enhancing the Success of Virtual Implementation**

- Establish a backup plan in case technology fails
- Allow/schedule time for informal interaction – outside of regular meetings (emails/calls)
- Plan for increased time
  - Without being able to rely on non-verbal forms of communications, it may take longer to check-in with each participant and to establish consensus.
- Pay attention to time zone differences
- Establish frequent contact early
  - Check-in regularly
- Enhance efforts to engage leadership
  - May be more challenging when done remotely and will need additional attention
- Attend to levels of investment and competing priorities
  - Be cognizant of appropriate timing to take action
  - Possible that competing priorities may not be as obvious as they would be to an on-site facilitator
  - Identify competing priorities as early as possible
  - Formally ask sites about other local initiatives and site-specific concerns

**Conducting Virtual Meetings**

- Solicit input frequently
  - Especially from quiet participants
  - Provide opportunities for all participants to give input
- Have a plan for back-channel communication
  - Checking-in informally improves processes (e.g. individual emails, Lync messages)
  - Ask how the meetings are going

**Promote Shared Understanding**

- Confusion about purpose and roles is magnified when relying solely on remote communications
- Build in check-in points early in the process to summarize information, provide opportunities for questions, and ask stakeholders to describe their understanding of the purpose and goals
- Promote a common sense of purpose – use screen sharing and group brain-storming
- Establish understanding of context and organizational structure early – “typical patient” and organizational structure

**Using Supportive Behavior**

- Pursue increased flexibility
  - Although it is recommended that all interactions have an agenda, be prepared to alter the agenda as needed in terms of both content and process
- Listen actively
  - Consistently follow up on any statements that are not fully clear, double check intended meanings, and ask additional questions that might not be needed when interacting face-to-face.
- Exhibit energy and enthusiasm
  - Facilitators must be aware the they may need to intentionally do more and take active steps to communicate their enthusiasm than they might in person

# Summary

The Coaching Manual for the Coordination Toolkit and Coaching (CTAC) Project is intended to provide a basic template for distance coaching in support of care coordination interventions by interdisciplinary quality improvement teams in primary care. The Coaching Manual is updated quarterly to incorporate additional resources and to adapt existing material with feedback from ongoing coaching.

# Management Tools

This is a list of management tools that you may find useful as you coach your teams.

| Tool | Name | Use |
| --- | --- | --- |
| A | [**Organizational Readiness Interview Guide**](#_Tool_A:_Organizational_1) | Use to assess the readiness of a facility to implement a QI project. |
| B | [**Organizational Readiness Checklist**](#_Tool_B:_Organizational) | Use to ensure you have not skipped any essential steps in your implementation efforts. Can be used to monitor progress on completing the organizational readiness activities. |
| C | [**Introduction and Overview for Stakeholders**](#_Tool_C:_Introduction_1) | Template information sheet that can be used to enlist support of senior leaders for the project. |
| D | [**Leadership Support Assessment**](#_Tool_D:_Leadership) | Can be used to assess senior leadership support for the project. |
| E | [**Business Case Form**](#_Tool_E:_Business) | Can be used to create a high-level overview of the case for the project. The information can be presented to senior leaders who will decide whether to support the project. |
|  | ***Site Visit Materials*** | Tools F-J can be compiled into a Site Visit Packet – Additional tools can be added to the packet as appropriate |
| F | [**Site Visit Table of Contents & Agenda**](#_Tool_F:_Site) | Can be used to organize the site visit. The table of contents is an example of toolkit resources that can be used for a site visit. |
| G | [**CTAC Project Staff List**](#_Tool_G:_CTAC) | Briefly introduces each person on the CTAC team. |
| H | [**CTAC Project Description**](#_Tool_H:_CTAC) | Brief introduction to the CTAC project. |
| I | [**QI Project Proposal Template and Sample**](#_Tool_F:_Sample) | Basic outline of the project including problems, goals, resources, and measures. |
| J | [**Patient Experience Survey (Hassles Scale)**](#_Tool_J._Patient_1) | Patient experience survey; focused on the 16-item Hassles Scale ( addresses problems accessing/receiving VA care) and additional patient information items. |
| K | [**Tool Catalog**](#_Tool_K:_Tool_1) | Lists care coordination tools and their descriptions. |
| L | [**CTAC Project Timeline for Participating Sites**](#_Tool_L:_CTAC_1) | Provides a potential timeline for the 12-month CTAC project. |
| M | [**Sample Weekly Timeline and Plan (Weeks 1-8)**](#_Tool_M:_Sample) | Use to keep the team on track and to document progress/barriers. |
| N | [**Meeting Call Log**](#_Tool_N._Meeting) | Tool to summarize discussions and results of team meetings. |
| O | [**Quarterly Leadership Call Topics**](#_Tool_G:_Quarterly) | Use to keep site leaders informed of project progress and keep them apprised of any changes/issues. |
| P | [**QI Project Action Plan Template and Sample**](#_Tool_H:_Quality) | Provides a framework for outlining steps that will be needed to design and implement a new program at the facility or clinic. |
| Q | [**SMART Goals Template**](#_Tool_J:_SMART) **and Sample** | A template to help the team define the goals and objectives of the QI project so that they are specific, measureable, achievable, realistic, and time-based. |
| R | [**A3 Worksheet**](#_Tool_K:_A3) | Helps to identify the project’s problem statement, aims, goals, current state, target state and plan of action. |
| S | [**Mid-Point Project Template**](#_Tool_L:_Mid-Point) | Use to track project progress and intermediate findings. |
| T | [**Final Project Template**](#_Tool_M:_Final) | Use to track project progress, results, and future sustainability plans. |
| U | [**Clinical Query Form**](#_Tool_U:_Clinical) | Use to request clinical support from CTAC project team. |
| V | [**Team Contact and Information Sheet**](#_Tool_O:_Interdisciplinary) | A contact and information sheet for the project site team and the Greater Los Angeles CTAC team. |
| W | [**Interdisciplinary Team**](#_Tool_W:_Interdisciplinary) | Identifies people from different disciplines to take part on the implementation team that will oversee the project. |
| X | [**Quality Improvement Process**](#_Tool_P:_Quality) | Identifies the extent to which the project has necessary resources for quality improvement in its environment. |
| Y | [**Stakeholder Analysis**](#_Tool_Y:_Stakeholder) | Helps program initiators identify which departments and individuals will have an interest in their project, where barriers might exist, and what actions need to be taken to obtain the buy-in and participation of those departments and individuals. |
| Z | [**PDSA Worksheet for Testing Change**](#_Tool_Z:_PDSA) | A worksheet to document a test of change for the project. |
| AA | [**Current Process Analysis**](#_Tool_T:_Current) | Describes key processes in the organization where project activities could or should happen. |
| BB | [**Assessing Staff Education and Training**](#_Tool_U:_Assessing) | Assesses current staff practices and facilitates integration of new practices. |
| CC | [**Managing Change Checklist**](#_Tool_V:_Managing) | Used to monitor your progress on completing the managing change activities. |
| DD | [**Implementation Checklist**](#_Tool_W._Implementation) | Can be used to monitor progress on implementation project. |
| EE | [**Assigning Responsibilities**](#_Tool_X._Assigning) | Determines who will be responsible for each task. |
| FF | [**Staff Roles**](#_Tool_Y._Staff) | Gives an example of how responsibilities may be assigned among different team members at the clinic where the project will be implemented. |
| GG | [**Sustainability Tool**](#_Tool_Z._Sustainability) | Can be used to identify sustainability issues in planning and implementing the new project. |

## Tool A: Organizational Readiness Interview Guide

Care Coordination QUERI: Project 1

**MASTER INTERVIEW GUIDE**

Version 12.05.16

- *INTRODUCE YOURSELF*

**Hello, my name is [*name of interviewer*].** *Introduce self and other team members on the phone.*

- *INTRODUCE THE PROJECT (This is QI not Research in the traditional sense.)*

The purpose of this quality improvement project is to **match clinics with an intervention for supporting best practices in care coordination**. Leadership at your facility has suggested your clinic as a potential match. We want to take this time to talk with you about your clinic and some of the challenges you face in delivering care, particularly with regard to effective care coordination.

**Note to interviewer:** *GOALS: (1) To decide if a clinic could participate in the project; (2) Gather baseline information for later on, to compare how well the clinic did in the project; (3) Facilitation: important to know the clinic’s strengths and weaknesses to identify an appropriate QI project.*

- *EXPLAIN THE PURPOSE OF THE INTERVIEW*

This interview will help us to better understand care coordination in your clinic. We will be interviewing people from multiple clinics to gain a variety of perspectives.

- *DESCRIBE THE AUDIO RECORDING AND CONFIDENTIALITY*

We would like to audio record this interview so that we have an accurate record of your thoughts. Please be assured that the recording and your transcript will be kept confidential. Neither leadership nor co-workers at your facility or VISN will have access to any of your responses, nor will they be able to connect your responses to you personally. Our project has been determined to be a non-research quality improvement activity by the Office of Primary Care in VA Central Office, and was also reviewed by the VA Greater Los Angeles Healthcare System’s IRB and determined to be non-research. Once your interview has been transcribed, only a site identifier will be linked to the transcripts, while any information linking you to the transcript will be destroyed. The audio recording will be destroyed as soon as the transcript is verified and analyzed by project staff.

To help ensure confidentiality, it would help if, wherever possible, you would refrain from mentioning the name of your facility or mentioning specific names of other staff members during the interview. If, at any time, you feel that the questions are too sensitive, I would be happy to turn off the recorder during that portion of questioning. You may also skip any questions you wish during the interview.

**Note to interviewer:** *If you don’t record make sure to debrief immediately after the interview****.***

**Do you have any questions for me?** *[Answer any questions]*

**About how much time do you have? Are you ready to begin?**

**I’m going to start recording now.**

**I. INTRODUCTION**

**I’m going to start with some general questions.**

**1. First, would you please describe your role within your facility?**

*PROBES*

- What is your title and role within your organization?
- Whom do you report to?
- How long have you been part of this organization?
- If changes in role, what prompted those changes? Probe for whether previous role is related to primary care/care coordination.
- Ask about prior experiences with care coordination and/or in other VA centers.

**II. CLINIC NEEDS AND STRUCTURES**

**Note to interviewer:** *Can ask #2 & 3 at the end if respondent has limited time for interview.*

**2. Can you tell me a little bit about your clinic?**

*PROBES*

- What it is like to work in your clinic?
- How are the PACT teamlets in your clinic set up?
- Are there any staffing vacancies within teamlets right now?
  - Probe about provider turnover
- Has there been any turnover in staff?
- Ask about non-clinical staff and their level of medical knowledge
- Ask about administration/administrative culture

**3. Can you tell me a little bit about your patient population?**

*PROBES: Care coordination depends a lot on the patients’ needs*

- What are the most common patient concerns in your clinic?
- Are most of your patients dual VA/non-VA care users, or mostly VA users, or some combination?
- [Looking beyond physical health, do patients in your clinic struggle with a lot of concerns, such as substance abuse, mental illness, homelessness, and/or lack of social support?]
- IF NECESSARY: What is a typical patient?

**4. What are the biggest problems your clinic is facing right now?**

**5. Are there problems related to care coordination in your clinic? We are particularly interested in issues coordinating patients’ care between this clinic and other parts of the outpatient system (for example, specialty care, lab, radiology, pharmacy, or outpatient care outside the VA) and making sure the right information gets back to the patient.**

*PROBES*

- Could you tell me about coordination problems you encounter between:
  - VA and care provided in the community?
  - PACT and specialty care?
- Could you tell me about coordination problems you encounter between:
  - PACT and other services, like the lab or radiology?
- Does your clinic have any current or past initiatives to improve care coordination? If yes, please describe.
- Performance measures around care coordination you are trying to improve.

**6. On a scale of 1 to 10, with 1 being “not a priority” and 10 being “highest priority”:**

**Considering all the priorities your clinic has over the next year, what is the priority of your clinic for improving coordination of care for Veterans?**

**Note to interviewer:** *Most common misconception: Care post-hospital discharge not focus of this project.*

**III. HISTORY OF PRACTICE CHANGE**

**7. I would like to know a little bit about how your clinic has changed things when improvement is needed. For example, if you had trouble getting test results back to patients in a timely manner and this was something you wanted to improve, how did you go about making that change?**

*PROBES*

- [If yes,] how does that generally come about?
- Does clinic staff meet regularly to suggest, discuss, or learn about potential process improvement efforts in the clinic?
- Can you give me an example of a recent practice change or new intervention? How did that go?
- What worked? What didn’t work? Is it still in place?
- Who was involved in making this change happen? Did any challenges or tensions arise as the changes were negotiated within the clinic?

**We are almost finished. I have just a few remaining questions for you.**

**8. If you were going to move toward implementing a new practice or process change related to care coordination, what potential obstacles would you be concerned about?**

**9. If you were moving toward trying to implement a change, what kinds of resources are there to support this kind of change in your clinic?**

**For example:**

- **Release time**
- **Data reports to see how your clinic is doing**
- **Having a person to go to with questions**
- **Having people who help with making changes, on the job training**
- **IT support?**

**V. CLOSING**

**Note to interviewer:** *By this time in the interview the answer to #10 may be clear. You may confirm this by referencing a specific program or practice change when asking the question, if appropriate.*

**10. If your clinic were to try and implement one new program or practice change right now, what do you think it should be?**

*PROBES*

- If you had to make a change with the resources you have available, what would it be?
- Ask about time constraints

**That is the last question in the interview….**

**11. Do you have any other thoughts, comments or questions about anything we’ve talked about today?**

**One final thought on our end --** we would like to follow up with the staff and providers at your clinic with a short 5-10 minute online survey. The questions in the online survey would be different than the ones we asked you today, and would focus on understanding your clinic’s unique culture. Specifically, this survey would help us get a broader perspective on how clinic staff and providers feel about working at the clinic and with each other, again, to help us understand whether the clinic would be a good fit for this project. We would send an e-mail with a link to the survey at the beginning of [Month]. Would you be able to provide us with a list of the staff and provider names in your clinic?

**Thank you very much for taking the time to speak with us!**

## Tool B: Organizational Readiness Checklist

| **Background:** This tool can be used to monitor your progress on completing the organizational readiness activities.  **Reference:** Adapted from a form developed by the AHRQ Falls Toolkit Research Team.  **How to use this tool:** Complete the checklist. This assessment is best suited for clinic site leads, practice managers, and administrators.  Use this tool to ensure you have not skipped any essential steps in your implementation efforts. |
| --- |

**Organizational Readiness Checklist**

| **Readiness Question** | **Assessment in Your Organization** | **Yes** | **No** |
| --- | --- | --- | --- |
| Does the organization promote a culture of change? | Facility or clinic culture focuses on a systems approach to change. |  |  |
| Why is change needed? | Facility or clinic’s specific reasons for change have been identified. |  |  |
| Do organizational members understand why change is needed? | Staff attitudes about care coordination have been assessed. |  |  |
|  | Assessment results have been analyzed to suggest awareness-building needs. |  |  |
| Is there a sense of urgency about the change? | Supporters who have a sense of urgency have been identified. |  |  |
|  | Efforts are underway to generate a sense of urgency if lacking. |  |  |
| Is there leadership support for this effort? | Leadership support has been assessed. |  |  |
|  | If necessary, efforts are underway to generate this support. |  |  |
|  | Senior leader champion or sponsor has been identified. |  |  |
| Who will take ownership of this effort? | A leader has been identified for the implementation effort. |  |  |
|  | This leader is now involved in the subsequent planning steps. |  |  |
| What kinds of resources are needed? | A preliminary list of needed human and material resources has been developed. |  |  |
|  | Commitments to provide those resources have been obtained or are forthcoming. |  |  |

## Tool C: Introduction and Overview for Stakeholders

| **Background:** This template can serve as a letter to key players in your facility or clinic to introduce them to the goals and purpose of your project.  **Reference:** Adapted from a template developed by the AHRQ Falls Toolkit Research Team.  **How to use this tool:** Adapt this letter as needed and present it to senior leaders to enlist their support before initiating your project. You may want to use [**Tool Y: Stakeholder Analysis**](#_Tool_Q:_Stakeholder) to identify individuals and departments that may have an interest in your project.  The letter template is provided below. |
| --- |

**Letter Template**

Dear [***Name***]:

We would like to inform you about a project in [***facility or clinic name***] to improve care coordination for our primary care patients. We hope that you will support this exciting new endeavor.

**Project Information**: We will be implementing a project to **[*Add information about the tool that will be implemented]****.*

**Benefits**: We expect this project to ***[Add information about expected outcomes]***

**Possible effect for you/your area:** This project may require changes in the *[****any pertinent department/clinic/team/other organizational section for which the leader is responsible****].* For example, the efforts of:

**[*Add a list of groups, services, and people who will be affected by the program. Include details about the issues each group will need to consider.]***

- Physicians:
- Nurses:
- Physician Assistants:
- Psychologists:
- Social Workers:
- Pharmacists:
- Care Managers:

**What will happen? [*Add details about how the program will be carried out*]**

**Everyone has a role:** Most important in this effort is a shift of thinking and culture. Your support in helping [***facility or clinic name***] staff make this shift is essential to the success of this effort. Thank you.

## Tool D: Leadership Support Assessment

| **Background:** This tool can be used to assess senior leadership support for your project.  **Reference:** Adapted from a form developed by the AHRQ Falls Toolkit Research Team, based on Public Health Ontario’s Facility-Level Situation Assessment: <http://www.publichealthontario.ca/en/eRepository/facility-level-situation-assessment.pdf>  **How to use this tool:** Complete the checklist. This assessment is best suited for clinic site leads, practice managers, and administrators.  Review the responses to ascertain the level of leadership support. If the response to several of these items is “no,” it could threaten the success of your improvement process. Analyze the areas where support is not evident and take steps to inform leadership about the urgency to change. |
| --- |

| **Leadership Support Assessment** | **Yes** | **No** |
| --- | --- | --- |
| The importance of care coordination is clearly articulated in the organization’s strategic plan. |  |  |
| There are dedicated resources (e.g., staff time) allocated for this type of project. |  |  |
| The organization has staff members who provide education and training on care coordination. |  |  |
| Care coordination is a priority within the facility. |  |  |
| The facility has implemented prior improvement projects in primary care. |  |  |
| There are visible role models/champions for this type of project |  |  |

## Tool E: Business Case Form

| **Background:** This tool can be used to create a high-level overview of the case for the project. The information gathered in this tool may be presented to the senior leaders who will decide whether to support your project.  **Reference:** The form was adapted from a template developed by Project Agency to help write a business case. Available at: [www.businessballs.com/project%20management%20templates.pdf](http://www.businessballs.com/project%20management%20templates.pdf)  **How to use this tool:** Complete the form with all the required information. In some cases, to complete an element of the form (e.g., section on initial estimates of cost and time), additional work will be required. This form is best suited for a facility or clinic administrator. Present the completed form to the senior leader who would support your project, and discuss the potential benefits. This leader may also find it valuable for the finance department to calculate the return on investment (ROI).  ROI = Net returns from improvement actions/Investment in improvement actions.  Additional information on ROI estimation is available in AHRQ’s QI^TM^ Toolkit Roadmap: <http://www.ahrq.gov/professionals/systems/hospital/qitoolkit/qiroadmap.html#roi> |
| --- |

**Business Case**

| Project Background (keep this brief) |
| --- |
| General Aims |
| Initial Risks |
| Expected Outcomes |
| Benefits of Implementing This Project |
| Initial Estimates of Cost and Time  $: Time: |
| Outcomes of the Business Case |
| Decision Form (Project Sponsor) |
| Date |

## Tool F: Site Visit Table of Contents & Agenda


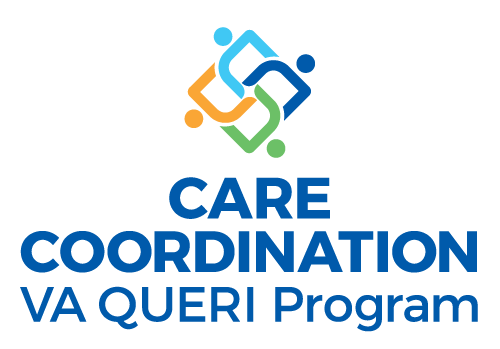


## Table of Contents

I. Project Information

- Project Team List
- Project Description
- Project Timeline

II. Patient Survey

- The Hassles Scale
- Patient Survey Results

III. Project Documents

- Project Proposal Template and Example
- Action Plan Template and Example

IV. References

- Performance Reports
- Toolkit Website and Tool List
- Systematic Review


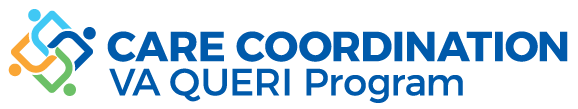


Coordination Toolkit and Coaching (CTAC) Project

[Site Name]

**[Date and time]**

AGENDA

| 1:00 pm | **Meeting of Clinic Quality Improvement Team and Coordination Toolkit and Coaching (CTAC) Project Team**   - Introductions - Coordination Toolkit and Coaching (CTAC) project summary and aims - Packet walkthrough including site specific patient survey data - Site discussions to date - Discussion of: - Clinic needs and priorities for care coordination - QI opportunities and resources - Effect on patient experience (Hassles scale) - Project Proposal - Roles and responsibilities - Coaching call schedule - Introduction to the Project Action Plan (as time permits) - Questions and Discussion - Next Steps   *Site Clinic Participants:*   - *Name, clinic role* |
| --- | --- |
| 3:30 pm | **Clinic Tour** |
| **Care Coordination Toolkit:** <https://vaww.visn10.portal.va.gov/sites/Toolkits/toolkit/Pages/Home.aspx> | |

## Tool G: CTAC Project Staff List

**
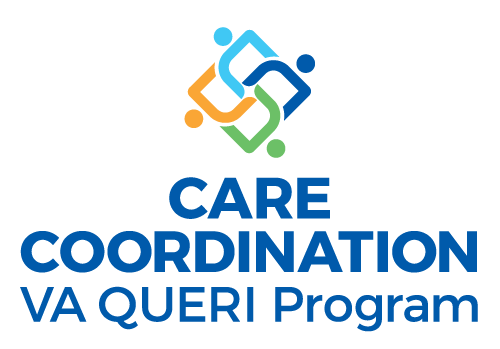
**

**Project Team List**

**David Ganz, MD, PhD** is the Principal Investigator (PI) for the CTAC project and Corresponding PI for the Care Coordination QUERI Program at the VA Greater Los Angeles Healthcare system. He is also a physician in the Geriatric Research, Education, and Clinical Center (GRECC) and Core Investigator in the HSR&D Center for the Study of Healthcare Innovation, Implementation and Policy (CSHIIP). He is also Associate Professor of Medicine at UCLA, and Adjunct Natural Scientist at RAND. He serves as Associate Director of the VA Quality Scholars program's Los Angeles site. Dr. Ganz developed the AHRQ Fall Prevention Toolkit for Hospitals, which involved collaboration with six geographically diverse hospitals, and features examples of successful implementation from the participating hospitals and other operations sites. Contact: [David.Ganz@va.gov](mailto:David.Ganz@va.gov)

**Jenny Barnard** is the CTAC Project Manager. She has over a decade of experience coordinating projects and programs in QUERI, HSR&D, the Center for Applied Systems Engineering (VA-CASE, the Veterans Engineering Resource Center based in Indianapolis, IN), and the Greater Los Angeles Geriatrics Research Education and Clinical Center (GRECC). She was part of the multi-site team who compiled, posted, and disseminated the web-based Quality Improvement Toolkit Series and the PACT Toolkit. Contact: [Jenny.Barnard@va.gov](mailto:Jenny.Barnard@va.gov)

**Tanya Olmos-Ochoa, PhD, MPH** is a Project Coach for CTAC. She is also a Social Science Specialist at the Mental Illness Research, Education and Clinical Center (MIRECC) for VISN22. She has experience in delivering research training to a variety of health care providers, including nurses and community health workers. She has a PhD from the UCLA Fielding School of Public Health in Health Policy and Management with a specialty in organizational behavior. Contact: [Tanya.Olmos@va.gov](mailto:Tanya.Olmos@va.gov)

**Neetu Chawla, PhD, MPH** is a Project Coach for CTAC and a Health Science Specialist. She has experience in working with provider teams and evaluating new policies implemented within hospitals and healthcare systems, such as the breastfeeding policies at Boston Medical Center and survivorship care planning within Kaiser Permanente Northern California. She has a PhD in Health Services Research from the University of California Los Angeles Fielding School of Public Health and an MPH in Community Health Sciences from the same institution. Contact: [neetu_chawla@hotmail.com](mailto:neetu_chawla@hotmail.com)

## Tool H: CTAC Project Description

**
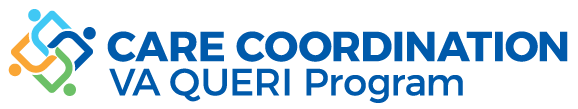
**

**Project Description**

The Improving PACT Coordination across Settings and Services: **Coordination Toolkit and Coaching (CTAC)** **project** aims to disseminate strategies for coordination of care for high-risk Veterans via an online toolkit, while evaluating the benefits of adding a distance-coaching strategy to assist sites with deploying the toolkit’s tools. The project’s focus is on care coordination across outpatient settings (e.g., from PACT to specialty care).

**Project summary:**

- 12-month project
- Weekly coaching calls (coached sites only)
- Patient survey (Hassles scale) administered at baseline and 12 months
- Phone interviews with champion, clinic team and leadership

**Project aims:**

1. Improving Veteran experience of care (as measured by the Hassles Scale)
2. Improving communication and coordination between PACT providers and specialists
3. Reducing inappropriate use of acute care services

**The project provides:**

- An online toolkit to support better care coordination for vulnerable patients visiting PACT
- Random assignment of participating clinics to either a toolkit or a combined toolkit/distance coaching strategy
- A QI approach with PDSA cycles of improvement, designed to support clinics in a locally initiated effort (coached sites only)

**Strategies being compared:**

- **Toolkit only**
- Priority-setting meeting with leadership to determine a topic of focus
  - - Access to online Care Coordination Toolkit
- **Toolkit and Distance Coaching**
- All “toolkit only” items, plus:
- In-person site visit at start
- Distance coaching by phone/video
- Coach will focus on quality improvement, project management, timeline and deliverables, and understanding data
- **All sites**
- Facility director signs endorsement letter allowing Veterans to participate in 20-item survey
- At the conclusion of the project CTAC team can provide consultation on spread of newly adopted tool(s) to other clinics

## Tool I: Quality Improvement Project Proposal Template and Sample

**Quality Improvement Project Proposal Template**

**To:
From:
Date:**

______________________________________________________________________________

**Innovation Title:**

**Problem:**

**Goals/Aim**^**:**

**Individuals responsible for meeting the project timeline:**

**Release Time Needed and Resources:**

**Measures and Action Plan**:

**Timeline:**

^Aim: Specific objectives the innovation seeks to address
^Goal: Overarching purpose or issue addressed by the innovation

**Tool I: Quality Improvement Project Proposal Template and Sample**


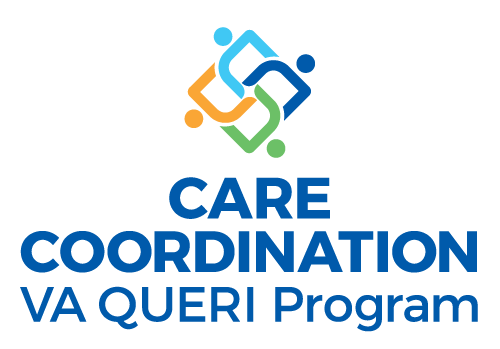


**Quality Improvement Project Proposal Sample**

**To:** CTAC Team
**From:** <<site>>
**Date:** 11/3/16

______________________________________________________________________________

**Innovation Title:** 1) Coordinating pain management with community providers to improve quality of care while maintaining safety issues concerning Veterans who use the Choice program.

**Problem:** 1) Very little, if any, coordination with community providers in tracking pain management treatment of Veterans. There is a safety issue concerning Opioid prescriptions.
 **Goals/Aim:** Seamless and timely coordination with community providers, specifically in tracking opioid treatments for Veterans in the <<cities>> geographical areas.

**Individuals responsible for meeting the project timeline:**

<<team members>>

**Release Time Needed and Resources:**

Release time for project champion Approx. 20-40 hrs. total time

Release time for Dr. Neill Approx. 10-20 hrs. total time

Release time for Pharmacy Approx. 10-20 hrs. total time

Release time for Choice Champion Approx. 5-15 hrs. total time

**Measures and Action Plan**:

Create a seamless coordination plan so that VA providers and community providers will have the tools in place to deliver timely information and care regarding Opioid Treatments & SAR’s to the correct points of contact.

**Timeline:**

Pilot Program: October 2016 – March 2017
^Aim: Specific objectives the innovation seeks to address
^Goal: Overarching purpose or issue addressed by the innovation

## Tool J. Patient Experience Survey (Hassles Scale)


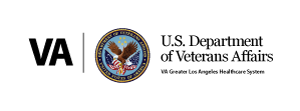
Save time by completing the survey online at [www.VAHealthcareExperience.com](http://www.VAHealthcareExperience.com)

**
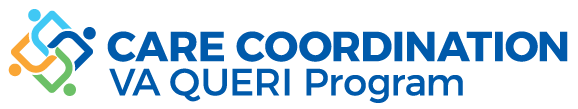
**

**Healthcare Experience Questionnaire**

We would like to start with a few questions about where you get your healthcare.

1. During the past 12 months, have you been seen by:

| Check one   - VA health care providers only - Both VA and non-VA health care providers - Non-VA healthcare providers only - Don’t know | **Please continue with the survey** |
| --- | --- |
|  | |
| - No health care providers | **We do not need you to complete the remainder of this survey. Please mail back your survey using the pre-paid envelope provided in your survey packet.** |

1. During the past 12 months, did you get any healthcare from a clinic or office that is not at a VA facility, but that the VA paid for? For example, through the Veterans Choice program or Fee basis care.

Check one

- Yes
- No
- Don’t know

1. During the past 12 months, have you used My HealtheVet to do any of the following:

Check all that apply

- Make an appointment for VA healthcare
- Contact my doctor using secure messaging
- Check on lab or test results
- Other reason (Specify: __________________________________________________________)

OR

- I did not use MyHealtheVet in the past 12 months

These next questions are about problems that people may experience when getting their healthcare. Please tell us about your experiences with these problems when you got healthcare during the past 12 months, either at VA or outside of VA.

1. During the past 12 months, how much of a problem, if at all, has each of the following been for you?

|  | **A very big problem for you** | **A big problem for you** | **A moderate problem for you** | **A small problem for you** | **Not a problem at all for you** |
| --- | --- | --- | --- | --- | --- |
|  | Check one on each line | | | | |
| 1. Lack of information about your medical conditions. | □ | □ | □ | □ | □ |
| 1. Lack of information about which treatment options are best for your medical condition. | □ | □ | □ | □ | □ |
| 1. Lack of information about why your medications have been prescribed to you. | □ | □ | □ | □ | □ |
| 1. Problems getting your medications refilled on time. | □ | □ | □ | □ | □ |
| 1. Uncertainty about when or how to take your medications. | □ | □ | □ | □ | □ |
| 1. Side effects from your medications. | □ | □ | □ | □ | □ |
| 1. Lack of information about why you’ve been referred to a specialist. | □ | □ | □ | □ | □ |
| 1. Having to wait a long time to get an appointment for specialty providers or clinics. | □ | □ | □ | □ | □ |
| 1. Poor communication between different healthcare providers. | □ | □ | □ | □ | □ |
| 1. Disagreements between your providers about your diagnoses or the best treatment for you. | □ | □ | □ | □ | □ |
| 1. Lack of information about why you need lab tests or x-rays. | □ | □ | □ | □ | □ |
| 1. Having to wait too long to find out about the results of lab tests or x-rays. | □ | □ | □ | □ | □ |
| 1. Difficulty getting questions answered or   getting medical advice between  scheduled appointments. | □ | □ | □ | □ | □ |
| 1. Lack of time to discuss all your problems with your health care provider during scheduled appointments. | □ | □ | □ | □ | □ |
| 1. Having your concerns ignored or overlooked by your healthcare providers. | □ | □ | □ | □ | □ |
| 1. Medical appointments that interfere with your work, family, or hobbies. | □ | □ | □ | □ | □ |

Finally, we would like to know a little bit more about you.

1. In general, how would you rate your overall *physical* health? *Check one*

- Excellent
- Very Good
- Good
- Fair
- Poor

1. In general, how would you rate your overall *mental or emotional* health? *Check one*

- Excellent
- Very Good
- Good
- Fair
- Poor

1. What is your age? *Check one*

- Under 24
- 25 to 34
- 35 to 44
- 45 to 54
- 55 to 64
- 65 to 74
- 75 to 84
- 85 or older

1. What is your gender?

- Male
- Female
- Other

1. Did you graduate from high school or earn a GED? *Check one*

- Yes
- No

1. What is your race/ethnicity or origin? *Check all that apply*

- White
- Black or African American
- Hispanic, Latino, or Spanish
- Asian or Asian American
- Native Hawaiian or Pacific Islander
- American Indian or Alaska Native
- Other_________________________________________
- Decline to answer

1. Are you currently married, in a civil union or domestic partnership, or living with a partner? *Check one*

- Yes
- No

**Thank you for completing this survey!**

Please mail back in the pre-paid envelope provided in your survey packet.

## Tool K: Tool Catalog

| **Tool Name** | **Tool Description** |
| --- | --- |
| **Tool List Abbreviated VA Formulary** | The Primary Care Service in the VA Palo Alto Healthcare System worked with its pharmacy department to create an abbreviated and searchable VA formulary. This resource was posted on the healthcare system’s external web page and is updated periodically for accuracy. |
| **After-Visit Instructions** | A customizable document instructing patients about where to go before leaving the VA the day of their appointment, as well as any follow-up actions which need to be taken. Includes contact information for specialty clinics as well as a map of the campus, which can be used to direct patients to their next destination on campus. |
| **VAIL After-Visit Summary** | An electronic tool that produces a customizable, printable patient summary that can be provided to a patient after his/her primary care office visit to summarize visit content and subsequent action steps, if appropriate. |
| **Audio Renewal Medication Renewal via Telephone** | Allows patients to request a renewal of their prescription directly from within the AudioCARE telephone refill system 24 hours a day, 7 days a week. |
| **Care Coordination Agreement Template** | A template with the purpose of facilitating timely access and patient-centered care for patients by promoting an effective relationship between the PACT team and the specialty care team during the management of a patient’s care. |
| **Clinic Information Pamphlet** | A template that clinics can customize to create a clinic information pamphlet for patients that includes pertinent information about the clinic. Information may include appointment-making instructions, provider contact information, prescription refill instructions, and other clinic details. |
| **Co-Managing the Veteran Patient’s Care and Medications** | A two-page document that includes a letter to help facilitate communication between a VA provider and an outside healthcare provider regarding management of the Veteran patient and instructions for obtaining medications through the VA, and a guide for providers (non-VA) to request a prescription medication that is non-formulary. |
| **Medication Log** | A one-page form with sections to be filled in by a member of the PACT team for the patient. Spaces are provided to fill in a patient's medication details and healthcare provider name and contact information. The medication list includes details that can help patients remember when to take their medications and what dose to take. |
| **Patient Decision Guide** | A worksheet for patients to use when faced with a medical decision. The form includes questions to ask the provider during the medical appointment, space to take notes, and guidance on how to proceed with making a decision. |
| **Pertinent Data Sets** | A comprehensive list of clinical conditions and for each, a standard set of information to be included in a referral request, to help ensure a meaningful visit when the referred patient visits the relevant specialist. While the tool consists of multiple data sets, not all need to be implemented. |
| **Pharmacy Safety** | A patient hand-out that explains the role of the pharmacist, pharmacy and patient during the process of obtaining medications. |
| **Primary Care Info Wallet Card** | A customizable wallet-sized card that can be printed, populated with a patient’s primary care team information and given to the patient. The back of the card includes a list of information patient’s should provide when leaving a message at the clinic. |
| **Questions Before, During and After your Appointment** | A guide that includes tips, ideas, and questions for patients to use before, during, and after their appointment that will help facilitate a successful healthcare visit. |
| **Relaxation and Meditation: An approach to self-management of mental health in primary care** | Information and tools for implementing a program that teaches patients relaxation and meditation skills, with the potential benefit of decreasing the number of referrals to specialty mental health services. |
| **Save A Trip – Key Phone and Web Contact Information for Primary Care Patients** | A one-page resource for patients, explaining and illustrating the difference between routine, non-urgent, and urgent medical situations. The sheet explains to patients what actions to take in the case of each of these situations, and provides relevant contact information. |
| **Tips for Improving Communication with your Primary Care Team** | A two-page handout that can be mailed to patients in a pre-visit packet or given to patients at check-in, before their appointment. The tool includes tips for communicating with the primary care team, questions for patients to think about before their appointment and space to write down answers. This tool can help ensure all of a patient’s questions and concerns are addressed during their medical appointment. |

## Tool L: CTAC Project Timeline for Participating Sites

**Coordination Toolkit and Coaching (CTAC)**

**Project Timeline for Participating Sites**

| **Requirements for facility as a whole** | |
| --- | --- |
| **Pre-project** |  |
| Leadership meeting | 1-2 one-hour phone meetings with key members of facility leadership (e.g., primary care director, nurse executive, chief of staff, facility director). Purpose is to ensure buy-in for the project. |
| Facility director signs letter endorsing patient experience survey | Survey endorsement letter sent out to Veterans with the survey mailing. |
| **Month 6** |  |
| Stakeholder interview | 1-hour phone interview with one facility level leader. |
| **Month 12** |  |
| Stakeholder interview | 1-hour phone interview with one facility level leader. |
| Final leadership meeting | 1 one-hour phone meeting with key members of facility leadership (e.g., primary care director, nurse executive, chief of staff, facility director).  Purpose is to review project results and discuss project sustainability, if applicable. |

| **Requirements for non-coached clinic(s)** | |
| --- | --- |
| **Pre-project** |  |
| Clinic readiness interviews | 1-hour phone interview with one lead at each potential clinic. |
| Online organizational readiness survey | A follow-up to the clinic readiness interviews, a brief online survey sent via e-mail to all PACT Team members of each clinic |
| Designate a project champion |  |
| **Month 6** |  |
| Stakeholder interview | 1-hour phone interview with the clinic’s project champion. |
| **Month 12** |  |
| Stakeholder interview | 1-hour phone interviews with the clinic’s project champion. |

| **Requirements for coached clinic(s)** | |
| --- | --- |
| **Pre-project** |  |
| Clinic readiness interviews | 1-hour phone interview with one lead at each potential clinic. |
| Online organizational readiness survey | A follow-up to the clinic readiness interviews, a brief online survey sent via e-mail to all PACT Team members of each clinic. |
| Designate a project champion |  |
| **Month 1** |  |
| Brief QI project proposal | Template provided |
| Project kick-off meeting | An in-person, ½ day meeting in which the coach and other GLA staff visit the coached clinic(s). |
| Weekly meetings between coach, champion and team | 1-hour phone meetings |
| **Month 2** |  |
| Weekly meetings between coach, champion and team | 1-hour phone meetings |
| Monthly all coached clinics call | 1-hour phone meeting including all coached project clinics, across sites. |
| **Month 3** |  |
| Weekly meetings between coach, champion and team | 1-hour phone meetings |
| Quarterly leadership meeting | If needed. Champion may update leadership separately. |
| Monthly all coached clinics call | 1-hour phone meeting including all coached project clinics, across sites. |
| **Month 4** |  |
| Weekly meetings between coach, champion and team | 1-hour phone meetings |
| Monthly all coached clinics call | 1-hour phone meeting including all coached project clinics, across sites. |
| **Month 5** |  |
| Weekly meetings between coach, champion and team | 1-hour phone meetings |
| Monthly all coached clinics call | 1-hour phone meeting including all coached project clinics, across sites. |
| **Month 6** |  |
| Weekly meetings between coach, champion and team | 1-hour phone meetings |
| Stakeholder interviews | 1-hour phone interviews with:  -The clinic’s project champion  -One frontline provider |
| Mid-way project report | Template provided |
| Quarterly leadership meeting | If needed. Champion may update leadership separately. |
| Monthly all coached clinics call | 1-hour phone meeting including all coached project clinics, across sites. |
| **Month 7** |  |
| Weekly meetings between coach, champion and team | 1-hour phone meetings |
| Monthly all coached clinics call | 1-hour phone meeting including all coached project clinics, across sites. |
| **Month 8** |  |
| Weekly meetings between coach, champion and team | 1-hour phone meetings |
| Monthly all coached clinics call | 1-hour phone meeting including all coached project clinics, across sites. |
| **Month 9** |  |
| Weekly meetings between coach, champion and team | 1-hour phone meetings |
| Quarterly leadership meeting | If needed. Champion may update leadership separately. |
| Monthly all coached clinics call | 1-hour phone meeting including all coached project clinics, across sites. |
| **Month 10** |  |
| Weekly meetings between coach, champion and team | 1-hour phone meetings |
| Monthly all coached clinics call | 1-hour phone meeting including all coached project clinics, across sites. |
| **Month 11** |  |
| Weekly meetings between coach, champion and team | 1-hour phone meetings |
| Monthly all coached clinics call | 1-hour phone meeting including all coached project clinics, across sites. |
| **Month 12** |  |
| Weekly meetings between coach, champion and team | 1-hour phone meetings |
| Monthly all coached clinics call | 1-hour phone meeting including all coached project clinics, across sites. |
| Stakeholder interviews | 1-hour phone interviews with:  -The project champion  -One frontline provider |
| Final project report and summary brief | A template is provided for the final report plus a 1-2 page summary of project and recommendations to leadership for next steps. |

##

**Tool L: CTAC Project Timeline for Participating Sites (Continued)**

| Months | 0 | 1 | 2 | 3 | 4 | 5 | 6 | 7 | 8 | 9 | 10 | 11 | 12 |
| --- | --- | --- | --- | --- | --- | --- | --- | --- | --- | --- | --- | --- | --- |
| **Clinic readiness interviews**  1-hour phone interview with one lead at each potential clinic. |  |  |  |  |  |  |  |  |  |  |  |  |  |
| **Online organizational readiness survey**  A follow-up to the clinic readiness interviews, a brief online survey sent via e-mail to all PACT Team members of each clinic. |  |  |  |  |  |  |  |  |  |  |  |  |  |
| **Designate a project champion** |  |  |  |  |  |  |  |  |  |  |  |  |  |
| **Brief QI project proposal**  Template provided |  |  |  |  |  |  |  |  |  |  |  |  |  |
| **Project kick-off meeting**  An in-person, ½ day meeting in which the coach and other GLA staff visit the coached clinic(s). |  |  |  |  |  |  |  |  |  |  |  |  |  |
| **Weekly meetings between coach, champion and team**  1-hour phone meetings |  |  |  |  |  |  |  |  |  |  |  |  |  |
| **Monthly all coached clinics call**  1-hour phone meeting including all coached project clinics, across sites. |  |  |  |  |  |  |  |  |  |  |  |  |  |
| **Quarterly leadership meeting**  If needed. Champion may update leadership separately. |  |  |  |  |  |  |  |  |  |  |  |  |  |
| **Stakeholder interviews**  1-hour phone interviews with:  -The clinic’s project champion  -One frontline provider |  |  |  |  |  |  |  |  |  |  |  |  |  |
| **Mid-way project report**  Template provided |  |  |  |  |  |  |  |  |  |  |  |  |  |
| **Final project report and summary brief**  A template is provided for the final report plus a 1-2 page summary of project and recommendations to leadership for next steps. |  |  |  |  |  |  |  |  |  |  |  |  |  |

## Tool M: Sample Weekly Timeline and Plan (Weeks 1-8)

The CTAC project spans 12 months, and while it is important for project sites to make initial progress at the start of the project, there can be great variation in the speed at which a team is formed and a project starts moving forward. Therefore, project teams will vary in their timeline and progress, especially in the first few months. The 12-month period allows for projects that may require a slower start, or need extra time to assemble their team. The following timeline provides guidance for the first 8 weeks of the project and includes suggestions for progress during these first few months. Tasks, action items, and outcomes will vary by site.

| **Week 1 – Week 2** | **Tasks:**   - Introductions - CTAC team and clinic site team (including champion and others from coached site, if present) - Discuss weekly team meeting schedule. If possible, pick a tentative weekly day and time. It can always change later if needed. - CTAC team shares project materials, reviews project focus, as needed (some on the clinic team may not have CTAC project background knowledge) - Discuss coach’s role in team meetings (may need to revisit this as new team members are added in the coming weeks) - Discuss quarterly leadership meeting schedule (to get on the calendar of leadership attendees) - Introduce the Project Proposal form - Review and revise Project Proposal, as needed - Finalize Project Proposal (complete this before moving on to the Action Plan) | **Action items for week 1-2:**   - Champion to work on and complete Project Proposal form. - Champion to meet with leadership to discuss and get sign-off on finalized Project Proposal form. - Coach to send Outlook invite for recurring weekly meetings. - Champion to identify and contact potential project team members. |
| --- | --- | --- |
| **Week 3 – Week 4** | **Tasks:**   - Introduce the Action Plan form - Discuss Action Plan during the calls. Team can revise between calls. Focus on: - Defining SMART Goals - Aims/measures - Once SMART Goals and Aims/measures are finalized, move on to the remainder of the Action Plan. Focus on project timeline - If complete team is not already assembled, discuss potential team members. (What type of people will we need on the team based on the type of project? Pharmacist, Psychologist, etc.) | **Action items for week 3-4:**   - Champion/team work on and complete first draft of Action Plan. - Revisions to Action Plan form continue. - If needed, champion contacts additional potential team members for inclusion on the project team. |
| **Week 5 – Week 6** | **Tasks:**   - Finalize Action Plan. If not finished with Action Plan, continue to focus on it. - Start discussion of existing sources for baseline data on chosen topic. - Discuss process mapping and how it applies to the chosen topic/project. Introduce example process maps (can use the examples in the coaching manual) and Visio program. - If time, start to work on process map together. | **Action items for week 5-6:**   - Final revisions to Action Plan, if needed. - Champion requests Visio program on their computer (or computer they typically use for the weekly calls). - Champion and team work on process map. |
| **Week 7 – Week 8** | **Tasks:**   - Continue discussion about baseline data, including a plan for collecting data (measurement section of Action Plan). - Review process map and edit as needed - Finalize process map - Discuss quarterly leadership meeting (date, attendees, presentation) | - Gather baseline data for needs assessment. - Champion and team continue to refine process map. - Send doodle poll or possible dates to leadership participants for 1^st^ leadership call |

## Tool N. Meeting Call Log

| **[DATE]**   - Introductions - Introduced project and pilot period expectations - Scheduled recurring weekly meetings | **Week 1** |
| --- | --- |
| **Prior to this call the facility champion received:**   - Site visit PP slides (since she was not involved at the time of the site visit) - The link to the Care Coordination Toolkit   **During this call:**   - Introductions - Overview of the project and detailed plans for the pilot period - Agreed upon a schedule of weekly meetings   **Action items:**  Greater Los Angeles team to send champion:   - [Facility] performance report (PACT compass and SHEP data) - QI project proposal form and example - Hassles survey - Outlook meeting invite for recurring meetings | |

| **[DATE]**   - Reviewed Project Proposal form - Introduced Action Plan form | **Week 2** |
| --- | --- |
| **During this call:**   - Reviewed the champion’s draft QI Project Proposal form (Tool I). - Discussed the possible parameters of the proposed project including narrowing down the two projects the champion had listed on the form – specifically deciding whether to focus on the educational piece or the business process piece. - Discussed the possibility of having a main priority and a sub-priority. - Brainstormed possible team members, including making contact with someone from the pharmacy. - Reviewed the fillable Action Plan Template (Tool P). - Champion noted that she is tracking her time spent on this project.   **Action items:**   - Champion to edit the Project Proposal form - Champion to start drafting the Action Plan form   **Note:** Although not an assigned action item, after this meeting the champion invited 3 individuals to attend the next meeting: [Names, Titles] | |

| **[DATE]**   - Worked on Action Plan form - Discussed task of flow mapping current pharmacy processes using Microsoft Visio | **Week 3**  Note: Team formed by champion and invited to the call – [Names, Titles] |
| --- | --- |
| **During this call:**   - Discussed the draft Action Plan template including the chosen project topic of [Detailed Topic Description]. - Discussed how a process flow mapping exercise will help provide insight into the details of this process and where changes could be made. - Additional tasks include identifying the high volume community providers and obtaining baseline data/measurements about how many Choice provider prescriptions are coming in monthly and how many of those prescriptions involve opiates.   **Action items:**   - Champion to obtain Microsoft Visio program on her computer - GLA team to send Visio YouTube instructional video to provide details about how to use the program <https://www.youtube.com/playlist?list=PLTtplW6mJ7fWSIvHScqHCkaA4T0HhaWU-> - Champion to draft a Visio flow map of pharmacy process - Champion to edit and revise Action Plan form based on discussion during today’s call   **Note:** [Technology Prep]: Before the 4^th^ call, GLA and champion had a short call to test out the screen sharing function of Adobe Connect and practice sharing her Visio screen in preparation for Week 4 call. | |

| **[DATE]**   - Presentation of draft process flow map - Discussed the survey process and provided survey endorsement letter - Reviewed Action Plan form – focused on refining SMART Goal #1. | **Week 4** |
| --- | --- |
| **During this call:**   - The champion shared her draft Visio flow map of the pharmacy process for opiate prescriptions. The group talked through the flow diagram, discussing changes in wording and structure/processes that need to be made. - The pharmacist described the baseline data she obtained regarding the volume of [VA Facility] prescriptions. - We reviewed the Action Plan form, focusing on SMART Goal #1, discussing ways to create a more specific and measurable goal. Specifically including the definition of a potentially unsafe prescription and how improvement would be measured, and then looking at how many potentially unsafe prescriptions were coming through over a given period of time.   **Action Items:**   - Pharmacist to think through a plan for piloting a triage of the prescriptions for 1 month. - QI team to revisit SMART Goal #1, making it more specific and measurable. - GLA team to send champion the entire survey mailing packet. - Champion to put endorsement letter on Facility letterhead and obtain the Director’s signature on the letter. | |

| **[DATE]**   - Revisited Project Action Plan – specifically SMART GOAL #1. - Reviewed Visio Process Map - Discussed Endorsement Letter and signature - Chief of Medicine retirement | **Week 5** |
| --- | --- |
| **During this call:**   - Discussed further defining SMART Goal #1. - We discussed the data collected by the pharmacist to find the baseline # of prescriptions coming through the pharmacy each month. - The next step is to map out how much work each prescription requires. Need to define the triggers for each scenario – how does the pharmacist know that something different needs to be done with these specific prescriptions? Create the path we want the providers to follow and create blocks for the process we want to stop. - Balancing measures were discussed – these are used to ensure that an unexpected consequence or equally unacceptable process is not created when the current process is changed. The state prescription drug monitoring program (PDMP) would help in this regard, since it allows [facility] pharmacists to see whether Veterans filled opiate prescriptions outside the VA. - Pharmacist also discussed new version of SMART Goal #3   **Action Items:**   - Team will add content to the Project Action Plan form – SMART Goals #1, #2, and #3 as well as the “measurement” sections. This may include web links to guidelines and/or benchmarks. - Edits to flow diagram will be made to flesh out the incoming prescription process. - Champion and team will start to think through specific activities to implement the project and will begin to look at the timeline in the Project Action Plan form. - Discussed potential replacement on team for Chief of Medicine due to retirement. - QI team tracking the time they are spending on this project and will include this information in the “Resources” section of the Action Plan document. | |

## Tool O: Quarterly Leadership Call Topics

| **Call #** | **Date** | **Topics** |
| --- | --- | --- |
| 1 | Q1 | Present action plan and the initial summary of first QI cycle of improvement. Leadership will help with troubleshooting barriers to QI implementation (e.g. personnel, resources, space, software, etc.). |
| 2 | Q2  (Mid-Point) | Using mid-project review document, present initial results of QI project focused on implementation or process outcomes (e.g., feasibility, adoption, resource implications in terms of workload, acceptability to providers and staff). |
| 3 | Q3 | Discuss to institutionalization of QI project. What can be done to make the project more sustainable? Work with leadership to develop a plan to maintain aspects of the project that work. |
| 4 | Q4 | Present draft of final report, with summary of results, implications for the facility, progress and sustainability, and additional steps needed to institutionalize the project. |

## Tool P: Quality Improvement Project Action Plan Template and Sample


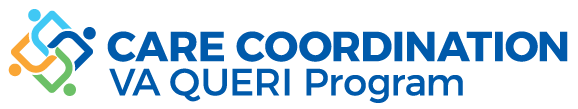

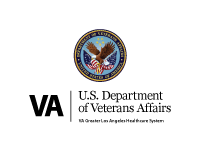


CTAC Project Proposed Action Plan

| Project Team Members |
| --- |
| Team Members & Positions:  Clinic or PACT Team:  Project Champion, Position:  Project Champion Phone:  Project Champion Email: |
| Intended Project: |
| 1. We plan to implement the following quality improvement project: 2. Adopting this project will enhance our program/practice in the following ways: 3. The target population/patients for our quality improvement project are: |

| Statement of Goals |
| --- |
| Please describe the best practice you plan to complete in order to achieve your overall quality improvement project goal. Use S.M.A.R.T. goals (Specific, Measureable, Achievable, Realistic/Results Oriented and Time Dated) to guide your plans.  S.M.A.R.T. Goal Example:  “By February 2015, all planning meetings will be paperless by using tablets so notes can be shared electronically to facilitate communication.”   - - Specific: What, Why, How     - What: all planning meetings will be paperless     - Why: to facilitate communication     - How: by using tablets   - Measurable: whether or not all meetings are paperless by 2/2015   - Achievable: participants have tablets and know how to use them   - Results Oriented: notes are shared electronically   - Time Dated: by 2/2015 |

| S.M.A.R.T. Goal #1 |
| --- |
| Goal description:  Specific: What will the goal accomplish? Why and How will it be accomplished?  What:  Why:  How:  Measurable: How will you measure whether or not the goal has been reached?  Achievable: Do you have the necessary knowledge, skills, and resources to accomplish this goal?  Results Oriented: What is the reason, purpose or benefit of accomplishing this goal?  Time Dated: What is the completion date? |

| S.M.A.R.T. Goal #2 |
| --- |
| Goal description:  Specific: What will the goal accomplish? Why and How will it be accomplished?  What:  Why:  How:  Measurable: How will you measure whether or not the goal has been reached?  Achievable: Do you have the necessary knowledge, skills, and resources to accomplish this goal?  Results Oriented: What is the reason, purpose or benefit of accomplishing this goal?  Time Dated: What is the completion date? |
| S.M.A.R.T. Goal #3 |
| Goal description:  Specific: What will the goal accomplish? Why and How will it be accomplished?  What:  Why:  How:  Measurable: How will you measure whether or not the goal has been reached?  Achievable: Do you have the necessary knowledge, skills, and resources to accomplish this goal?  Results Oriented: What is the reason, purpose or benefit of accomplishing this goal?  Time Dated: What is the completion date? |

| Assessment |
| --- |
| Which QI methods do you plan to use during your quality improvement project? (Flow Chart, Plan, Do, Study, Act cycle, etc.)  *Enter description here.* |
| Measurement |
| Which source of data do you plan to use to measure your project process and outcomes? (Administrative data, chart audits, surveys, etc.)  *Enter description here.* |
| Thinking through activities to implement your project |
| 1. Commitment from Organizational Leadership: Identify who needs to be aware of your action plan and goals for this QI project. Are they at the table already? If not, how will you get buy-in from necessary stakeholders?   We will need to community with the following people:   \| **Person’s name and title** \| **Who will communicate with them?** \| \| --- \| --- \| \| 1. \|  \| \| 2. \|  \| \| 3. \|  \| \| 4. \|  \|  1. Resources: Identify what other resources you will need to accomplish your plan. Resources may include: personnel, time, financial requirements, leadership, and support from a committee or administrator.   We will need the following resources:        You will be asked how much time you spent on this project:   1. Track time of [activity & team member]: 2. Track time of [activity & team member]: 3. Track time of [activity & team member]: 4. Track time of [activity & team member]: 5. Barriers: Please identify potential obstacles or barriers to implementing your plan.   We anticipate the following obstacles/barriers:       5. Strategies to overcome barriers:   We will use these strategies to overcome the obstacles outlined above: |

| Time Frame |
| --- |
| The quality improvement project should be completed over the course of 12 months. In order to stay on track for delivering your innovation, below is a Gantt chart for you to complete. Please list your S.M.A.R.T. goals, who will lead the effort, and whether or not you will need the assistance of your coach. At this point you should only complete “proposed” (top line), you’ll complete “actual” in real time. S.MA.R.T. Goals #2 & #3 are on the next page.   \| **S.M.A.R.T. GOAL #1** \| **WHO WILL LEAD**  **THIS EFFORT?** \| **NEED COACH’S**  **ASSISTANCE?** \|  \| **1** \| **2** \| **3** \| **4** \| **5** \| **6** \| **7** \| **8** \| **9** \| **10** \| **11** \| **12** \| \| --- \| --- \| --- \| --- \| --- \| --- \| --- \| --- \| --- \| --- \| --- \| --- \| --- \| --- \| --- \| --- \| \| **1.** \|  \|  \| **Proposed** \|  \|  \|  \|  \|  \|  \|  \|  \|  \|  \|  \|  \| \| Actual \|  \|  \|  \|  \|  \|  \|  \|  \|  \|  \|  \|  \| \| **2.** \|  \|  \| **Proposed** \|  \|  \|  \|  \|  \|  \|  \|  \|  \|  \|  \|  \| \| Actual \|  \|  \|  \|  \|  \|  \|  \|  \|  \|  \|  \|  \| \| **3.** \|  \|  \| **Proposed** \|  \|  \|  \|  \|  \|  \|  \|  \|  \|  \|  \|  \| \| Actual \|  \|  \|  \|  \|  \|  \|  \|  \|  \|  \|  \|  \| \| **4.** \|  \|  \| **Proposed** \|  \|  \|  \|  \|  \|  \|  \|  \|  \|  \|  \|  \| \| Actual \|  \|  \|  \|  \|  \|  \|  \|  \|  \|  \|  \|  \| \| **S.M.A.R.T. GOAL #2** \| **WHO WILL LEAD**  **THIS EFFORT?** \| **NEED COACH’S**  **ASSISTANCE?** \|  \| **1** \| **2** \| **3** \| **4** \| **5** \| **6** \| **7** \| **8** \| **9** \| **10** \| **11** \| **12** \| \| **1.** \|  \|  \| **Proposed** \|  \|  \|  \|  \|  \|  \|  \|  \|  \|  \|  \|  \| \| Actual \|  \|  \|  \|  \|  \|  \|  \|  \|  \|  \|  \|  \| \| **2.** \|  \|  \| **Proposed** \|  \|  \|  \|  \|  \|  \|  \|  \|  \|  \|  \|  \| \| Actual \|  \|  \|  \|  \|  \|  \|  \|  \|  \|  \|  \|  \| \| **3.** \|  \|  \| **Proposed** \|  \|  \|  \|  \|  \|  \|  \|  \|  \|  \|  \|  \| \| Actual \|  \|  \|  \|  \|  \|  \|  \|  \|  \|  \|  \|  \| \| **4.** \|  \|  \| **Proposed** \|  \|  \|  \|  \|  \|  \|  \|  \|  \|  \|  \|  \| \| Actual \|  \|  \|  \|  \|  \|  \|  \|  \|  \|  \|  \|  \| \| **S.M.A.R.T. GOAL #3** \| **WHO WILL LEAD**  **THIS EFFORT?** \| **NEED COACH’S**  **ASSISTANCE?** \|  \| **1** \| **2** \| **3** \| **4** \| **5** \| **6** \| **7** \| **8** \| **9** \| **10** \| **11** \| **12** \| \| **1.** \|  \|  \| **Proposed** \|  \|  \|  \|  \|  \|  \|  \|  \|  \|  \|  \|  \| \| Actual \|  \|  \|  \|  \|  \|  \|  \|  \|  \|  \|  \|  \| \| **2.** \|  \|  \| **Proposed** \|  \|  \|  \|  \|  \|  \|  \|  \|  \|  \|  \|  \| \| Actual \|  \|  \|  \|  \|  \|  \|  \|  \|  \|  \|  \|  \| \| **3.** \|  \|  \| **Proposed** \|  \|  \|  \|  \|  \|  \|  \|  \|  \|  \|  \|  \| \| Actual \|  \|  \|  \|  \|  \|  \|  \|  \|  \|  \|  \|  \| \| **4.** \|  \|  \| **Proposed** \|  \|  \|  \|  \|  \|  \|  \|  \|  \|  \|  \|  \| \| Actual \|  \|  \|  \|  \|  \|  \|  \|  \|  \|  \|  \|  \| |

**Tool P: Quality Improvement Project Action Plan Template and Sample**

| 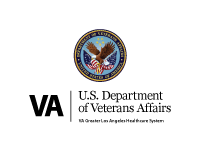 | 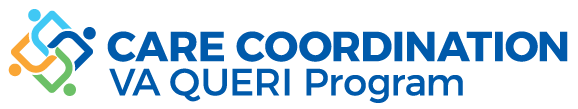 |
| --- | --- |
| CTAC Project Proposed Action Plan – Sample | |
| **CONTACT INFORMATION** | |
| **Project Champion, Position: <<staff name>>**  **Team Members & Positions:**  <<team members>>  **Project Champion Contact Information:**  **Phone:** <<phone>>  **Email:** <<email>> | |

| **Intended Project:** |
| --- |
| **1) We plan to implement the following quality improvement project:**   \| Coordinating pain management with community providers to improve quality of care while addressing safety issues concerning Veterans who use the Choice program. \| \| --- \|   **2) Adopting this project will enhance our program/practice in the following ways:**  1. Decrease unsafe prescriptions and avoid new prescriptions to high risk patients.  2. Exchange clear communication of clinical information between VA & community providers.  3. Education regarding universal precautions in compliance with VA/CDC.  **3) The target population/patients for our quality improvement project are:**  1. Veterans in the <<cities>> catchment area who are utilizing the CHOICE program.  a. Specifically, High Risk and Prior Risk patients. |
| **S.M.A.R.T. Goal #1** |
| \| Reduce CHOICE opioid prescription new starts not meeting CDC guidelines #4 and 5 to zero. \| \| --- \|   **Specific:** This will ensure safe pain management for veterans receiving CHOICE opioid prescriptions.  **What:**   - **CDC Guideline #4:** When starting opioid therapy for chronic pain, clinicians should prescribe immediate-release opioids instead of extended-release/long-acting (ER/LA) opioids. - **CDC Guideline #5:** When opioids are started, clinicians should prescribe the lowest effective dosage. Clinicians should use caution when prescribing opioids at any dosage, should carefully reassess evidence of individual benefits and risks when considering increasing dosage to morphine milligram equivalents (MME)/day, and should avoid increasing dosage to ≥90 MME/day or carefully justify a decision to titrate dosage to ≥90 MME/day.   **Why:** Veterans are at increased risk for adverse events from opioid use.  **How:** All CHOICE opioid prescriptions received by SORCC pharmacy will be directed to PACT/Pain pharmacist.  Guideline #4: If the SORCC pharmacy receives a new opioid start prescription written for extended release/long acting opioid, the PACT/Pain pharmacist will contact the prescribing provider’s office to request the new prescription be written for a VA formulary short-acting opioid.  Guideline #5: If the MME dosage≥ 90 MME, the PACT/Pain pharmacist will contact the prescribing provider’s office for the following criteria for use (CFU) documentation::   - Signed Consent documenting risks & benefits as well as patient and provider responsibilities - Recent State Prescription Drug Monitoring data - Risk Assessment - Recent Urine Drug Screen   **Measurable:** Measured by Data which is by PACT/Pain Pharmacist- All opioid CHOICE prescriptions, prescriptions that trigger action, CHOICE provider responses.  **Achievable:**  We have the necessary knowledge, skills and resources to accomplish this goal here at VA SORCC, however, staff constraints with availability of time remains a problem.  **Results Oriented:** The reason or purpose of accomplishing this goal is the benefit to the Veteran to ensure safe opioid use for pain management and compliance with CDC guidelines.  **Time Dated:** January 31^st^, 2017 |
| **S.M.A.R.T. Goal #2** |
| \| Ensure 100% compliance with CDC Guideline #8 for Veterans receiving new CHOICE opioid prescriptions meeting documented criteria. \| \| --- \|   **Specific:** Safe pain management and risk mitigation for veterans receiving CHOICE opioid prescriptions.  **What:** Clinicians should incorporate into the management plan strategies to mitigate risk, including considering offering naloxone when factors that increase risk for opioid overdose, such as history of overdose, history of substance use disorder, higher opioid dosages (≥50 MME/day), or concurrent benzodiazepine use, are present.  **Why:** Veterans are at higher risk for adverse events from opioid use.  **How:** All CHOICE opioid prescriptions received by SORCC pharmacy will be directed to PACT/Pain pharmacist.   - All opioid prescriptions for Veterans with documented history of overdose, history of Substance Use Disorder, or MED ≥ 50 or concurrent benzodiazepine use will be contracted for education and dispensing of naloxone.   **Measurable:** This will be measured by VISN 20 Opioid Risk Registry, OEND Dashboard, and the STORM Dashboard.  **Achievable**: We have the necessary knowledge, skills, and resources to accomplish this goal.  **Results Oriented:** The reason or purpose of accomplishing this goal is to ensure Veterans who are at risk for overdose have access to the lifesaving medication naloxone.  **Time Dated:** January 31^st^, 2017   \| **S.M.A.R.T. Goal #3** \| \| --- \| \| \| Exchange clear communication of clinical information between VA & community providers regarding universal precautions in compliance with pain management with VA/CDC guidelines. \| \| --- \|   **Specific:** This will bring VA and Community Providers together in an atmosphere of cooperation. This will provide seamless coordination of tracking opioid treatment.  **What/Why:** To keep Veterans safe.  **How:** Provide 2-3 in-service events to community providers before March 31^st^, 2017. Establishing relationship and communication with community providers by bringing them together in a face to face event.  **Measurable:**   - 1. Need a list of CHOICE Providers   2. Draft a standardized education information letter for CHOICE Primary Care & Pain Specialists, and also CHOICE Surgeons.   3. VA Pain Champion Provider & Pharmacy to do education at the in- services.   **Achievable**: Resources to accomplish this goal:______________  <<champions and other staff>>  **Results Oriented:** The reason or purpose of accomplishing this goal is to ensure that community providers are aware of VA and CDC’s guidelines for appropriate prescribing of opiates.  **Time Dated:** April 30, 2017 \| |

| **AssessmenT** |
| --- |
| Which QI methods do you plan to use during your quality improvement project?   1. Pilot test of change 2. Audit and feedback 3. Flow Chart (see below by <<staff>>)    |
| **MEASUREMENT:** |
| Which sources of data do you plan to use to measure your project process and outcomes? (Administrative data, chart audits, surveys, etc.)  Administrative data on filled prescriptions |
| **thinking through activities to implement YOUR PROJECT** |
| 1. **Commitment from Organizational Leadership:** Identify who needs to be aware of your action plan and goals for this QI project. Are they at the table already? If not, how will you get buy-in from necessary stakeholders?   **We will need to communicate with the following people:**   \| **Person’s name and title** \| **Who will communicate with them?** \| \| --- \| --- \| \| <<Chief of Staff>> \| Care Coordination Team \| \| Community Providers who write pain medication prescriptions \| <<Clinical Pharmacy Specialist>> \| \| Community Providers \| <<Choice Champion>> \| \| VA SORCC Staff  Community Providers \| <<Care Coordination Champion>> \|  1. **Resources:** Identify what other resources you will need to accomplish your plan. Resources may include: personnel, time, financial requirements, leadership, and support from a committee or administrator.  - Continuity of leadership. - 1 hour/week for all team members for project team meetings - X hours/week from <<physician>> and Y hours/week from <<staff>> to implement SMART goals #1 and #2. - B hours/week from <<physician>> and C hours/week from <<physician>> to implement SMART goal #3.   **C. Barriers:** Please identify potential obstacles or barriers to implementing your plan**.**  *Is this a durable plan of change?*  *Can we use the Program Drug Monitoring Program that Oregon state now has in place?*  **We anticipate the following obstacles/barriers:**   1. AD/PAC staffing, extra duties to already overburdened staff. 2. Limited knowledge of community providers regarding appropriate prescribing. 3. Already complicated workflow for handling of CHOICE prescriptions   **D. Strategies to overcome barriers:**   1. Use a pilot test to verify feasibility of approach 2. In-service prescribing providers in the community about appropriate prescribing 3. Develop standard workflow for processing of CHOICE prescriptions |

| TIME FRAME |
| --- |
| The quality improvement project should be completed over the course of 6 months (pilot only). In order to stay on track for delivering your innovation, below is a Gantt chart for you to complete. Please list your S.M.A.R.T. goals, who will lead the effort, and whether or not you will need the assistance of your coach. At this point you should only complete “proposed” (top line), you’ll complete “actual” in real time.   \| **S.M.A.R.T. GOAL #1** \| **WHO WILL LEAD**  **THIS EFFORT?** \| **NEED COACH’S**  **ASSISTANCE?** \|  \| **Dec** \| **Jan** \| **Feb** \| **Mar** \| **Apr** \| **May** \| \| --- \| --- \| --- \| --- \| --- \| --- \| --- \| --- \| --- \| --- \| \| Decrease CHOICE opioid prescription new starts not meeting CDC guidelines #4 and #5 \| <<staff>> \| **Yes, for evaluation** \| **Proposed** \| **X** \| **X** \|  \|  \|  \|  \| \| Actual \| **X** \| **X** \|  \|  \|  \|  \| \| **S.M.A.R.T. GOAL #2** \| **WHO WILL LEAD**  **THIS EFFORT?** \| **NEED COACH’S**  **ASSISTANCE?** \|  \| **Dec** \| **Jan** \| **Feb** \| **Mar** \| **Apr** \| **May** \| \| Ensure compliance with CDC guideline #8 for Veterans receiving CHOICE opioid prescriptions. \| <<staff>> \| **Yes, for evaluation** \| **Proposed** \| **X** \| **X** \|  \|  \|  \|  \| \| Actual \| **X** \| **X** \|  \|  \|  \|  \| \| **S.M.A.R.T. GOAL #3** \| **WHO WILL LEAD**  **THIS EFFORT?** \| **NEED COACH’S**  **ASSISTANCE?** \|  \| **Dec** \| **Jan** \| **Feb** \| **Mar** \| **Apr** \| **May** \| \| Exchange clear communication of clinical information between VA & community providers regarding universal precautions in compliance with pain management with VA/CDC.  (Getting letter included with new CHOICE primary care authorization) \| <<staff>> \| **Yes, for potential educational materials** \| **Proposed** \|  \|  \| **X** \| **X** \|  \|  \| \| Actual \|  \|  \|  \| **X** \|  \|  \| |

## Tool Q: SMART Goals Template and Sample

This tool uses an example from the Clinician Guide to SMART Goal-setting developed by the VA National Center for Health Promotion and Disease Prevention (NCP) to describe SMART Goals^[[31]](#footnote-31)^.

**Example 1:** If the goal of your project is to help providers facilitate Veterans’ management of their own health care, your *Not-so-SMART* goal might be:

- Providers will help Veterans to better understand the medications they are taking

To make this goal into a SMART goal, use the following template:

| **Key Component** | **Objective** |
| --- | --- |
| **S**pecific – What is the specific task? |  |
| **M**easurable – What are the standards or parameters? |  |
| **A**chievable – Is the task feasible? |  |
| **R**ealistic – Are sufficient resources available? |  |
| **T**ime-Bound – What are the start and end dates? |  |
| **SMART Objective:** | |

| **Key Component** | **Objective** |
| --- | --- |
| **S**pecific – What is the specific task? | When prescribing a new medicine to a Veteran on ≥ 4 existing prescription drugs, ask the Veteran to indicate names and frequency of medications he/she is currently taking. Cross-check the list against the electronic health record, and reconcile discrepancies. |
| **M**easurable – What are the standards or parameters? | Denominator: Number of Veterans with ≥ 4 prescription drugs prescribed a new medicine during the project period (defined via automated pharmacy data). Numerator: Number of Veterans who had a medication review (defined by completion of clinical reminder). |
| **A**chievable – Is the task feasible? | These activities are within the scope of practice of PACT team members, and there are tools/resources to facilitate medication discussion. |
| **R**ealistic – Are sufficient resources available? | Yes, to move proportion with review from baseline of 50% to target of 75% by month six. |
| **T**ime-Bound – What are the start and end dates? | Six months between January 2017 and June 2017 |
| **SMART Objective:** By the month of June 2017, for Veterans on ≥ 4 existing prescription drugs being prescribed a new medication, increase the proportion with a medication review to 75%. | |

## Tool R: A3 Worksheet


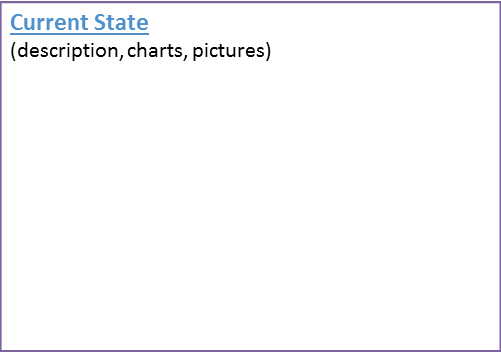

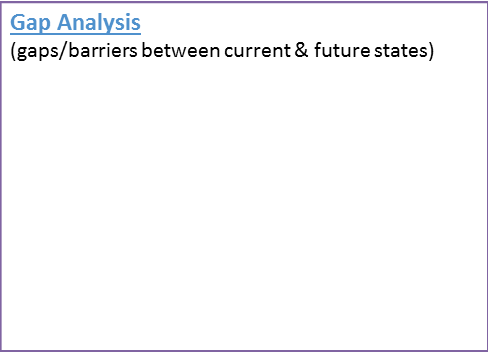

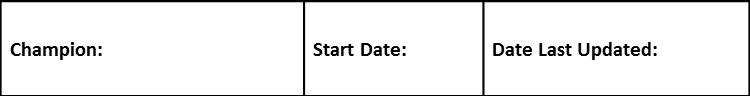

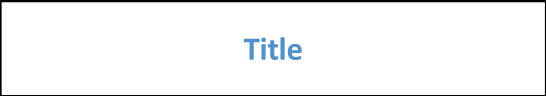

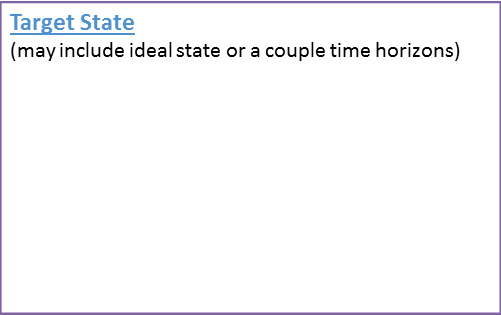

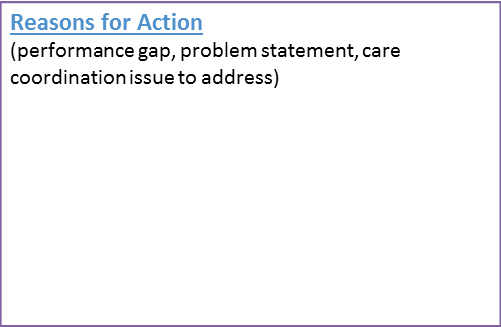

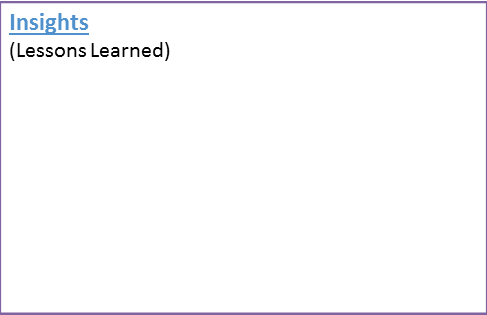

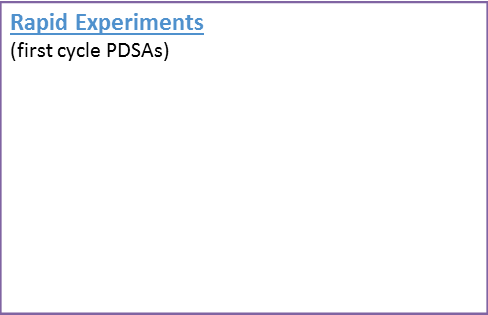

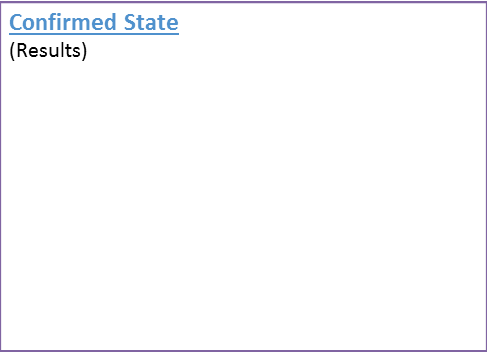

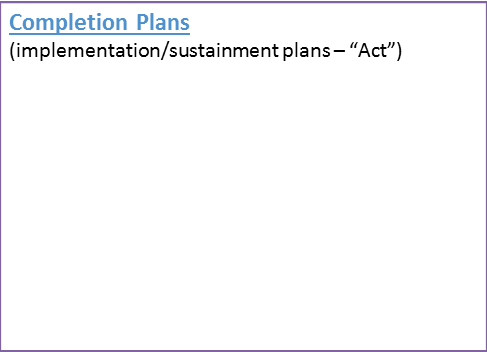

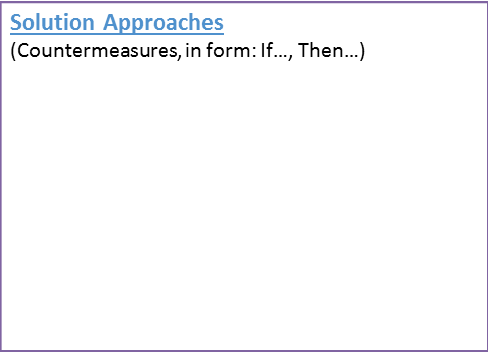

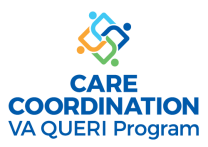


## Tool S: Mid-Point Project Report Template

**PROJECT TITLE/DESCRIPTION**

**BACKGROUND**

- Briefly describe your clinic (ex. Size, number of PACT teams, urban/rural, any unique features of the Veteran population you care for, etc.)
- Describe the problem your clinic identified.

**PURPOSE OF THE PROJECT**

- Describe your project (include title of your project).
- Describe the reason you chose to work on addressing this problem.

**PROJECT GOALS**

- Describe the project goals (May use the SMART Goals from the Project Action Plan).

**METHODS**

- Measure development for each goal (include SMART goals here):
  - The measure(s) developed to track the goal(s)
  - The data source(s) for the measure(s)
  - The method for analysis
- What did you do during the project?
  - Project Activities – Describe what you did
- How did you evaluate whether your project was successful?
  - You can use information from the Action Plan that is helpful to you.

**INTERIM FINDINGS/FINDINGS TO DATE**

- What are the early findings?
- What needs to be modified to improve outcomes?

## Tool T: Final Project Report Template

**PROJECT TITLE/DESCRIPTION**

**BACKGROUND**

- Briefly describe your clinic (ex. Size, number of PACT teams, urban/rural, any unique features of the Veteran population you care for, etc.)
- Describe the problem your clinic identified.

**PURPOSE OF THE PROJECT**

- Describe your project (include title of your project).
- Describe the reason you chose to work on addressing this problem.

**PROJECT GOALS**

- Describe the project goals (May use the SMART Goals from the Project Action Plan).

**METHODS**

- Measure development for each goal (include SMART goals here):
  - The measure(s) developed to track the goal(s)
  - The data source(s) for the measure(s)
  - The method for analysis
- What did you do during the project?
  - Project Activities – Describe what you did
- How did you evaluate whether your project was successful?
  - You can use information from the Action Plan that is helpful to you.

**FINDINGS**

- What are the results?
  - Include key findings and discuss them in this section.
  - Any tables and/or figures related to the key findings can be referenced in this section and included in the Appendices.

**IMPLICATIONS AND RECOMMENDATIONS**

- Lessons learned
  - Explain what the results mean and what you learned from them.
- Recommendations
  - What do you recommend that facility, VISN, or VA Central Office leadership do based on the results?
  - Describe planned/needed sustainability efforts.

**APPENDICES**

- Flow charts (e.g. process charts, Gantt chart, Fishbone diagram, etc.)
- Tables (e.g. glossary of terms and acronyms)
- Figures (e.g. letters developed, flyers, etc.)

## Tool U: Clinical Query Form

| **Clinical Query Form** | | | |
| --- | --- | --- | --- |
| **Clinic Name &**  **Champion Name:** |  | **Meeting Date:** |  |
|  |  | **Next Meeting Date:** |  |
| **Response Need:** | **□ Urgent** | **□ Not Urgent** | |
|  | | | |
| **Meeting Summary:** |  | | |
| **Need for Clinical Query:** |  | | |
| **Clinical Response:** |  | | |

## Tool V: Team Contact and Information Sheet

| VA Facility |  |
| --- | --- |
| Clinic Name |  |
| Name and Title of VISN Leader Endorsing the Project (if applicable) |  |
| Name and Title of Facility Leader Endorsing the Project |  |

|  | **Picture**  [ask each team member for a digital photo and insert here] (optional) | **Name** | **Position** | **Contact Information**  **(email and telephone)** |
| --- | --- | --- | --- | --- |
| **1** |  |  | Project Champion |  |
| **2** |  |  | Coach |  |
| **3** |  |  |  |  |
| **4** |  |  |  |  |
| **5** |  |  |  |  |
| **6** |  |  |  |  |
| **7** |  |  |  |  |
| **8** |  |  |  |  |
| **9** |  |  |  |  |
| **10** |  |  |  |  |

## Tool W: Interdisciplinary Team

| **Background:** Crucial to the introduction of a new project is the creation of an interdisciplinary implementation team that will oversee the effort. This tool can be used to identify people from different disciplines to take part on the implementation team.  **Reference:** Adapted from a tool developed by the AHRQ Falls Toolkit Research Team  **How to use the tool:** Use the list to form your implementation team. This tool should be filled out by the implementation team leader. List the names of possible team members form each department or discipline and their area of expertise.  The core implementation team should be a reasonable size (e.g., 6-12 people) to be effective. Additional staff may be included on an “as needed” basis. When you create a new team or invite new members to a team, make sure to set aside time for introductions at the beginning of your team meeting. |
| --- |

**Tool W: Interdisciplinary Team (Continued)**

| **Position/Discipline** | **Names of Possible Implementation Team Members from Each Area** | **Area of Expertise** |
| --- | --- | --- |
| **PACT Teamlet** | | |
| Primary Care Providers (e.g., Physician, physician assistants, NP) |  |  |
| Nurse care managers |  |  |
| LPN/LVN |  |  |
| Clerk/AMSA |  |  |
| **Extended PACT (varies by facility)** | | |
| Social Worker |  |  |
| Pharmacists |  |  |
| Psychologist |  |  |
| Physical therapists |  |  |
| Occupational therapists |  |  |
| Educator |  |  |
| Registered dietitian |  |  |
| Health Tech |  |  |
| **Organizational Management** | | |
| Senior manager |  |  |
| Quality improvement/safety/risk manager |  |  |
| Facility leadership |  |  |
| **Facilities and Environment** | | |
| Environmental services staff |  |  |
| Facilities engineer |  |  |
| **Other** | | |
| Information systems staff |  |  |
| Patient representative |  |  |
| Volunteer |  |  |

## Tool X: Quality Improvement Process

| **Background:** This tool will help you and your team identify the extent to which you have the resources for quality improvement (QI) in your organization. The form is one part of a larger tool developed by the Turning Point Initiative. This part of the tool assesses if an organization has the needed systems in place to improve quality and performance.  **Reference:** Public Health Foundation. Performance Management Self-Assessment Tool. The entire tool is available at: <http://www.phf.org/focusareas/performancemanagement/Pages/Access_the_Performance_Management_Self_Assessment_Tool.aspx>  **How to use the tool:** This tool should be filled out by the implementation team leader (or individual designated by the leader) in consultation with the QI department. The “you” refers to your organization as a whole. Check the box that most accurately describes your organization’s current resources. If you find that your organization has fully operationalized QI processes, connect the new project with these existing processes. If some processes are missing, advocate for them to be put into place in the context of your new project. |
| --- |

**Quality Improvement Process**

| **Assessment Question** | **No** | **Somewhat** | **Yes (fully operational)** |
| --- | --- | --- | --- |
| Do you have a process(es) to improve quality or performance? |  |  |  |
| Is an entity or person responsible for decision making based on performance reports (e.g., top management team, governing or advisory board) |  |  |  |
| Is there a regular timetable for your QI process? |  |  |  |
| Are the steps in the process communicated? |  |  |  |
| Are managers and employees evaluated for their performance improvement efforts (i.e., is performance improvement in their job descriptions)? |  |  |  |
| Are performance reports used regularly for decision making? |  |  |  |
| **Is performance information used to do the following? (check all that apply)** | | | |
| Determine areas for more analysis or evaluation. |  |  |  |
| Set priorities and allocate/redirect resources. |  |  |  |
| Inform policymakers of the observed or potential impact of decisions under their consideration. |  |  |  |
| Do you have the capacity to take action to improve performance when needed? |  |  |  |
| Do you have processes to manage changes in policies, projects, or infrastructure? |  |  |  |
| Do managers have the authority to make certain changes to improve performance? |  |  |  |
| **(Cont’d) Is performance information used to do the following? (check all that apply)** | **No** | **Somewhat** | **Yes (fully operational)** |
| Does staff have the authority to make certain changes to improve performance? |  |  |  |
| Does the organization regularly develop performance improvement or QI plans that specify timelines, actions, and responsible parties? |  |  |  |
| Is there a process or mechanism to coordinate QI efforts among divisions, or organizations that share the same performance targets? |  |  |  |
| Is QI training available to managers and staff? |  |  |  |
| Are personnel and financial resources allocated to your QI process? |  |  |  |

## Tool Y: Stakeholder Analysis

| **Background:** The purpose of stakeholder analysis is to help project initiators identify which departments and individuals will have an interest in their new project, where barriers might exist, and what actions need to be taken to obtain the buy-in and participation of those departments and individuals.  **Reference:** This tool was adapted from a template developed by Project Agency, a company focused on effective project management, and is available at: <http://projectagency.co.uk/documents/b316stakeholderform.pdf>  **How to use this tool:** Complete the form with information regarding all the individuals you consider key stakeholders. You may need to set up a meeting with them to obtain their answers. Examples: information technology officer, quality improvement (QI) department, ambulatory care, mental health, nursing, pharmacy, social work, medicine.  This form should be completed by the individuals interested in and affected by the new project.  Once the form is completed, identify actions needed to involve all stakeholders in the project. Ensure that all identified needs have been addressed before proceeding with implementation of the project. For example, the project may need process assistance from the QI department. Since this project may be competing with other QI priorities, it may be important to determine who shapes the QI agenda and how to get this project prioritized at a higher level.  An example is shown in the form below. A blank form follows. |
| --- |

| **Stakeholder** | **Interest or requirement in the project** | **What the project needs from the stakeholder** | **Perceived attitudes and risks** | **Actions to take** |
| --- | --- | --- | --- | --- |
| **Example:** Health Information Systems Officer | Gatekeeper for making any changes to the electronic health record (EHR) system. Not necessarily interested in the project beyond his general mandate to keep the EHR tied to clinical documentation needs. | The project may need to add or make changes to any parts of the EHR that concern care coordination. | May not want to make changes until other changes are also in process, or other changes may already be in process. | Seek information about the process for requesting/making these kinds of changes and how this person relates in the overall organizational structure to project leaders/advocates. |

**Tool Y: Stakeholder Analysis (Continued)**

| **Stakeholder** | **Interest or requirement in the project** | **What the project needs from stakeholder** | **Perceived attitudes and risks** | **Actions to take** |
| --- | --- | --- | --- | --- |
|  |  |  |  |  |
|  |  |  |  |  |
|  |  |  |  |  |
|  |  |  |  |  |
|  |  |  |  |  |
|  |  |  |  |  |
|  |  |  |  |  |
|  |  |  |  |  |
|  |  |  |  |  |

## Tool Z: PDSA Worksheet for Testing Change

***AIM:*** (overall goal you wish to achieve) – *Every goal will require multiple smaller test of change*

| **Describe your first (or next) test of change:** | **Person responsible** | **When to be done** | **Where to be done** |
| --- | --- | --- | --- |
|  |  |  |  |

***Plan***

| **List the tasks needed to set up this test of change** | **Person responsible** | **When to be done** | **Where to be done** |
| --- | --- | --- | --- |
|  |  |  |  |

| **Predict what will happen when the test is carried out** | **Measures to determine if prediction succeeds** |
| --- | --- |
|  |  |

***Do* Describe what actually happened when you ran the test**

***Study* Describe the measured results and how they compared to the predictions**

***Act* Describe what modifications to the plan will be made for the next cycle from what you learned**

Institute for Healthcare Improvement

## Tool AA: Current Process Analysis

| **Background:** Before beginning a quality improvement initiative, you need to understand your current methods. This tool can be used to describe key processes in your organization where project activities could or should happen.  **Reference:** Adapted from Quality Partners of Rhode Island (New name: Healthcentric Advisors). QI Worksheet E, Current Process Analysis.  **How to use this tool:**   - Identify who will conduct the mapping and who will be on the mapping team. The mapping team should include at least two frontline staff on the implementation team and at least one person who has experience with process maps. Try to use the same team members if more than one process is mapped. - Have the implementation team identify and define every step in the current process. - Define a beginning, an end, and a methodology for all the processes to be mapped. For example, some processes are mapped through the method of direct observation of the process taking place, while others can be mapped by knowledgeable stakeholders talking through and documenting each step in the process. - When defining a process, think about staff roles in the process, the tools or materials staff use, and the flow of activities. - Everything is a process, whether it is seeing a patient, or managing a clinic. Identify key processes involving the proposed project. The goal of defining a process is to hone in on vulnerabilities and potential issues/failures in the current process. - Examples of processes might include patient check-in in clinic (e.g., when does it occur, who does it) or delivering feedback.   Determine if there are any gaps and problems in your current processes, and use the results of this analysis to systematically change these processes. |
| --- |

**Process Analysis Procedures**

- Take time to brainstorm and listen to every team member. Make sure the process is understood and documented.
- Make each step in the process very specific.
- Use one post-it note, index card, or scrap piece of paper for each step in the process. Lay out each step, move steps, and add and remove steps until the team agrees on the final process.
- If a process does not exist (for example, there is no process to ensure a medication review on an annual basis), identify the related processes (for example, the process for prescribing medications).
- If the process is different on different days of the week, identify each individual process.

**Tool AA: Current Process Analysis (continued)**

**Example: Process for Making Buttered Toast**

| **Step** | **Definition** |
| --- | --- |
| **1** | Check to see if there is bread, butter, knife, and toaster. |
| **2** | If supplies are missing, go to the store and purchase them. |
| **3** | Check to see if the toaster is plugged in – if not, plug in the toaster. |
| **4** | Check setting on toaster – adjust to darker or lighter as preferred. |
| **5** | Put a slice of bread in toaster. |
| **6** | Turn toaster on. |
| **7** | Wait for bread to toast. |
| **8** | When toast is ready, remove from toaster and put on plate. |
| **9** | Use knife to cut pat of butter. |
| **10** | Use knife to spread butter on toast. |

**Identify the steps of your defined process:**

- Press people for details.
- At the end of the gap analysis, compile the results in a document that displays each step so that team members have the map of the current process in front of them during the team discussion (Step 2).

**Hold team discussion**

**Evaluate your current process as you define it:**

- What policies and procedures do we have in place for this process?
- What forms do we use?
- How does our physical environment support or hinder this process?
- Which staff is involved in this process?
- Which parts of this process do not work?
- Do we duplicate any work unnecessarily? Where?
- Are there any delays in the process? Why?

**Continue asking questions that are important in learning more about this process.**

## Tool BB: Assessing Staff Education and Training

| **Background:** The purpose of this tool is to assess current staff education practices and to facilitate the integration of new knowledge – related to the project – into existing or new practices.  **Reference:** Adapted from Facility Assessment Checklist developed by Quality Partners of Rhode Island. Available at: <http://healthinsight.org/Internal/assets/Nursing%20Home/PRU%20-%20Facility%20Assessment%20Checklist.pdf>  **How to use this tool:** Complete the form by checking the response that best describes your facility. This tool should be filled out by the implementation team leader or designee in collaboration with the other team members. |
| --- |

**Facility Assessment**

Date:

1. **Does your facility or clinic have initial and ongoing education related to the proposed project for all relevant staff?**

☐ **No**. If no, this is an area for improvement. ☐ This is an area we are working on. ☐ Yes.

1. **Does your facility‘s education program related to the proposed project include the following components?**

|  | **Yes** | **No** | **Person Responsible** | **Comments** |
| --- | --- | --- | --- | --- |
| Are new staff assessed for their need for education on topics related to the project? |  |  |  |  |
| Are current staff provided with ongoing education on the principles related to the project? |  |  |  |  |
| Does education of staff provide discipline-specific education for issues related to the project? |  |  |  |  |
| If applicable, is there a designated clinical expert available at the facility to answer questions from all staff about the project? |  |  |  |  |
| Is the education provided at the appropriate level for the learner? |  |  |  |  |
| Does the education include staff training on documentation methods related to the proposed project? |  |  |  |  |

1. **In which areas of knowledge does the assessment suggest staff need more education?**

## Tool CC: Managing Change Checklist

| **Background:** This tool can be used to monitor your progress on completing the managing change activities.  **Reference:** Adapted from a tool developed by the AHRQ Falls Toolkit Research Team.  **How to use this tool:** The implementation team leader (or individual designated by the leader) should complete the checklist upon starting his/her role as leader and review the checklist quarterly thereafter.  Use this tool to ensure you have not skipped any essential steps in your project efforts. |
| --- |

**Managing Change Checklist**

| **Task** | **Status** |
| --- | --- |
| Implementation team composition |  |
| Team leader identified and in place |  |
| Members with necessary expertise/role identified and invited |  |
| Linkage to senior leadership defined and established |  |
| Team startup |  |
| Team agenda and charge clearly stated |  |
| Necessary training and resources in place for team to get started |  |
| Assessment |  |
| Current state of practices assessed |  |
| Current practice and policies systematically examined |  |
| Challenges to good practice identified at organization and practice levels |  |
| Staff knowledge assessed |  |
| Starting the work of redesign |  |
| Approaches to redesign explored and chosen |  |
| Gap analysis conducted between current practice and recommended practice |  |
| Setting goals and plans for change |  |
| Specific goals set |  |
| Plan initiated for making changes to meet those goals |  |
| Preliminary plan in place for sustaining the changes |  |

## Tool DD. Implementation Checklist

| **Background:** This tool can be used to monitor your progress on implementing the project.  **Reference:** Developed by the AHRQ Falls Toolkit Research Team.  **How to use this tool:** The implementation team leader (or individual designated by the leader) should complete the checklist.  Use this tool to ensure you have not skipped any essential steps in your project efforts. |
| --- |

**Implementing best practices checklist**

| **Task** | **Date Completed** |
| --- | --- |
| **Roles and Responsibilities of Staff** | |
| **Assign specific roles and responsibilities to:** | |
| Members of the Clinic Team |  |
| Clinic champion |  |
| **Organizing the Work** | |
| Identify paths of ongoing communication and reporting |  |
| Develop mechanisms to address accountability |  |
| Identify strategies for building new practices into daily routine |  |
| Refine preliminary implementation plan |  |
| Ensure support from key stakeholders |  |
| Initiate plan to pilot test new practices |  |
| Establish strategy for engaging staff |  |
| Create education plans to help staff learn new practices |  |

## Tool EE. Assigning Responsibilities

| **Background:** This tool can be used to determine who will be responsible for each task identified in the project. One way to generate interest and buy-in from the staff is to ask them to self-assign their responsibilities from a prioritized list of tasks that need to be accomplished.  **Reference:** Developed by the AHRQ Falls Toolkit Research Team.  **How to use this tool:** Complete the table by entering the different tasks and the specific individuals who will be responsible for completing each task. This tool should be filled out by the implementation team leader in collaboration with the other team members.  Use this tool to assign and clarify the roles and responsibilities of each staff member. Types of staff and the types of responsibilities they might take on are summarized in [**Tool FF: Staff Roles**](#_Tool_Y._Staff) |
| --- |

| **Task** | **Who will be responsible?** |
| --- | --- |
| Example:  Work with facility Clinical Application Coordinators to install the note template. | Example:  Systems Redesign Specialist |
|  |  |
|  |  |
|  |  |
|  |  |
|  |  |
|  |  |

## Tool FF. Staff Roles

| **Background:** This table gives an example of how responsibilities may be assigned among different team members at the clinic where the program will be implemented.  **Reference:** Developed by the AHRQ Falls Toolkit Research Team.  **How to use this tool:** The implementation team leader can use this tool to help assign specific individuals or groups to each task in [**Tool EE: Assigning Responsibilities**](#_Tool_X._Assigning). |
| --- |

| **Staff** | **Roles** |
| --- | --- |
| Physician |  |
| Nurse care manager |  |
| Nurse practitioner |  |
| Physician assistant |  |
| Pharmacist |  |
| Clerk |  |
| Licensed practical/vocational nurse |  |
| Psychologist |  |
| Social Worker |  |

## Tool GG. Sustainability Tool

| **Background:** This tool can be used to identify sustainability issues in planning and implementing your new project.  **Reference:** Adapted from AHRQ Falls Prevention Toolkit Sustainability Tool (<http://www.ahrq.gov/professionals/systems/hospital/fallpxtoolkit/fallpxtk-tool6a.html>).  Original source: Edwards JC, Feldman PH, Sangl J, et al. Sustainability of partnership projects: a conceptual framework and checklist. Jt Comm J Qual Patient Saf 2007;33(12 Suppl):37-47.  **How to use this tool:** The implementation team leader (or individual designated by the leader) should complete this checklist. |
| --- |

| **Elements of Sustainability** | **Notes** |
| --- | --- |
| Identity (Goals) | |
| Is guiding vision clearly specified? |  |
| Is change goal focused (not too encompassing) and actionable? |  |
| Is “sustainability goal” clarified (i.e., what will be sustained?) and at what level is this goal?   - Specific process or outcome - General capacity to improve on more than one outcome or process - Partnership itself |  |
| *Problem solving:* If vision and goals are not clearly specified, focused, and actionable, what strategies will be adopted to address this issue? |  |
| Infrastructure | |
| Human resources |  |
| - Are the internal/external human resources in place to sustain the effort going forward (describe—e.g., team, senior leadership, champion, opinion leader)? |  |
| - Are external supports in place to sustain the effort going forward (describe—e.g., mentors, advisory group, professional associations, community advocates)? |  |
| Technical resources |  |
| - Are materials developed and accessible if sought? |  |
| - Are there list serves, meetings, and other mechanisms to promote ongoing communication? |  |
| - Is training and technical support available to develop and maintain necessary skills? |  |
| - Are information systems in place to support the effort going forward? |  |
| Financial resources |  |
| - Is funding adequate for the time period required to achieve the change goal? |  |
| - Is funding source stable for the time period needed to accomplish guiding vision? |  |
| *Problem solving:* If key elements are lacking, has a strategy been developed to address this issue? |  |
| Incentives |  |
| Is project perceived to add value within the organization (i.e., people can see something in it for them)? |  |
| Can value be measured quantitatively (i.e., via a return on investment calculation)? |  |
| Are other intangible values/incentives perceived (e.g., improved reputation, pride, sense of accomplishment)? Describe. |  |
| Is the project perceived as having disincentives? Describe. |  |
| *Problem solving:* If positive incentives are inadequate or disincentives are identified, have strategies been proposed to address this issue? |  |
| Incremental Opportunities for Participation |  |
| Can the project goals be best achieved with varied levels and types of participation?  If **yes**, then continue to next two questions. |  |
| Are there opportunities for varied geographic participation (e.g., a single clinic versus an entire facility)? |  |
| 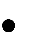 If yes, what types of varied geographic participation opportunities are available? |  |
| 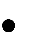 Is the geographic scale workable? |  |
| Are varied roles for participation in the project provided? If **yes**, what varied roles for participation are provided? |  |
| - Observer role |  |
| - Technical assistance role |  |
| - Data collection role (e.g., review charts or incident reports) |  |
| - Advisor or consultant role |  |
| - Implementer role |  |
| - Changing role throughout the project |  |
| - Can pick and choose among offered activities |  |
| - Can opt out and then opt back in later |  |
| - Other |  |
| *Problem solving:* If goals can be achieved with varied levels and types of participation but no provision has been made for participation in different ways, what strategies can be used to address this issue? |  |
| Integration |  |
| Are change goals aligned with strategic goals of participating entities (macro level)? |  |
| Are change goals integrated with other performance measures and reward systems of participating entities (macro level)? |  |
| Are change goals integrated with existing programs, policies/procedures, and information systems of participating entities (micro level)? |  |
| *Problem solving:* If change goals are not aligned and integrated with the strategic goals, performance measures, reward systems, programs, policies/ procedures, and information systems of participating entities, what strategies can be used to address this issue? |  |

# Appendix A: Resources

**Quality Improvement (QI)/Implementation Research (IR) Ethics and Compliance Toolkit** – An online toolkit with resources to help researchers identify and address the ethical issues to consider when conducting quality improvement and implementation research projects. <http://vaww.portal.gla.med.va.gov/sites/Research/HSRD/CIPRS/QIEthics/Pages/default.aspx>

**VAIL Team Communication Exercise (TEX) II Toolkit** – An online toolkit to assist PACT teamlets improve team function, including: task work, team work, and team processes. <http://vaww.portal.gla.med.va.gov/sites/Research/HSRD/VAILPCC/vtkits/t2/Pages/tk_home.aspx>

**Team Development Measure (TDM)** – A tool to help with team evaluation and identification of more effective team strategies to improve cohesion, communication, team roles, and team goals. An overview of the measure can be found here: <https://vaww.infoshare.va.gov/sites/primarycare/DemoLabs/TDM/default.aspx?PageView=Shared&InitialTabId=Ribbon.WebPartPage&VisibilityContext=WSSWebPartPage>

The tool can be found here: <http://vaww.vssc.med.va.gov/TeamDevelopmentMeasure/>

**QUERI Quality Improvement (QI) Methods webpage and selection tool** – A website for QUERI researchers with links to useful quality improvement methods and tools. <http://www.queri.research.va.gov/implementation/quality_improvement/default.cfm>

**VA Virtual Teams Handbook: Creating engaged and effective teams** – A handbook designed by the VHA National Center for Organization Development (NCOD) with resources and tools for working with virtual teams.

<http://vaww.va.gov/NCOD/docs/virtualteamshandbook.PDF>

1. David A. Ganz, MD, PhD; Lisa Rubenstein, MD; Jenny Barnard; Nina Smith, MPH; Hemen Saifu, MPH, Tanya Olmos-Ochoa, PhD, MPH [↑](#footnote-ref-1)
2. Additional contributions were made from many sources and are cited in the body of the manual. [↑](#footnote-ref-2)
3. Adapted from VA Office of Academic Affiliations (OAA) Centers of Excellence in Primary Care Education Implementation Kit [↑](#footnote-ref-3)
4. Ibid. [↑](#footnote-ref-4)
5. Adapted from VISN 22 PACT Demonstration Laboratory Evidence Based Quality Improvement (EBQI) Toolkit (VAIL) [↑](#footnote-ref-5)
6. Adapted from the UCLA Scan project, which borrowed from the VA Geriatric Scholars Program [↑](#footnote-ref-6)
7. Adapted from the CDC’s Public Health Information Network Communities of Practice [↑](#footnote-ref-7)
8. Adapted from the UCLA SCAN project, which borrowed from the VA Geriatric Scholars Program [↑](#footnote-ref-8)
9. Adapted from National Center for Patient Safety’s Virtual Breakthrough Series (NCPS) Initial Team Summary [↑](#footnote-ref-9)
10. Adapted from VA Office of Academic Affiliations (OAA) Centers of Excellence in Primary Care Education Implementation Kit [↑](#footnote-ref-10)
11. Adapted from AHRQ – Practice Facilitation Handbook: Module 14. Creating Quality Improvement Teams and QI Plans; <https://www.ahrq.gov/professionals/prevention-chronic-care/improve/system/pfhandbook/mod14.html> [↑](#footnote-ref-11)
12. Adapted from VA Office of Academic Affiliations (OAA) Centers of Excellence in Primary Care Education Implementation Kit [↑](#footnote-ref-12)
13. Ibid. [↑](#footnote-ref-13)
14. Adapted from VA Geriatric Scholars Program: The Foundations of Health Quality Improvement [↑](#footnote-ref-14)
15. Adapted from VA Office of Academic Affiliations (OAA) Centers of Excellence in Primary Care Education Implementation Kit [↑](#footnote-ref-15)
16. Chang, E.T., Wells, K.B., Young, A.S., Stockdale, S., Johnson, M.D., Fickel, J.J., Jou, K., & Rubenstein, L.V. (2014). The anatomy of primary care and mental health clinician communication: a quality improvement case study. *Journal of General Internal Medicine, 29(2),* S598-606. [↑](#footnote-ref-16)
17. Ibid. [↑](#footnote-ref-17)
18. Ibid. [↑](#footnote-ref-18)
19. Adapted from VA Office of Academic Affiliations (OAA) Centers of Excellence in Primary Care Education Implementation Kit [↑](#footnote-ref-19)
20. Adapted from Aspire Consulting, VISN 21 Team Development Measure (TDM) Facilitation Manual [↑](#footnote-ref-20)
21. Adapted from VA Office of Academic Affiliations (OAA) Centers of Excellence in Primary Care Education Implementation Kit [↑](#footnote-ref-21)
22. Adapted from the UCLA SCAN program, which borrowed from the VA Geriatric Scholars Program [↑](#footnote-ref-22)
23. Adapted from VA Office of Academic Affiliations (OAA) Centers of Excellence in Primary Care Education Implementation Kit [↑](#footnote-ref-23)
24. Adapted from VA Geriatric Scholars Program: The Foundations of Health Quality Improvement [↑](#footnote-ref-24)
25. Ibid. [↑](#footnote-ref-25)
26. Adapted from VA Office of Academic Affiliations (OAA) Centers of Excellence in Primary Care Education Implementation Kit [↑](#footnote-ref-26)
27. Ibid. [↑](#footnote-ref-27)
28. Ibid. [↑](#footnote-ref-28)
29. Adapted from a worksheet created by the New England VERC [↑](#footnote-ref-29)
30. Adapted from Aspire Consulting, VISN 21 Team Development Measure (TDM) Facilitation Manual [↑](#footnote-ref-30)
31. http://vaww.infoshare.va.gov/sites/prevention/NCP_Training_Resources/Shared%20Documents/HLM%20Training%20and%20Resources/Clinician%20Guide%20to%20SMART%20Goal-Setting%20Using%20the%20My%20Health%20Choices%20Tool%2010.22.12.docx [↑](#footnote-ref-31)
