# Supplementary material for: Toolkit and distance coaching strategies: a mixed methods evaluation of a trial to implement care coordination quality improvement projects in primary care
Source: BMC Health Serv Res. 2021 Aug 14;21:817. doi: 10.1186/s12913-021-06850-1 (PMC8364700; doi:10.1186/s12913-021-06850-1)
Supplement: Supplementary file 4 — Additional file 4. Guide for Assessing Site CTAC QI Project Complexity. Dimensions and criteria used to assess the complexity of sites’ CTAC projects. [file 12913_2021_6850_MOESM4_ESM.docx]

**Additional file 4. Guide for Assessing Site CTAC QI Project Complexity**

The following is a modified version of the Complexity Assessment Tool for Systematic Reviews (iCAT_SR).(1)

| Core Dimension | Assessment Criteria | Assessment Level | Description with Examples |
| --- | --- | --- | --- |
| 1. Active components included in the intervention in relation to usual care | *More than one component and delivered as a bundle* | *3* | The intervention includes more than one component and some or all of these components need to be delivered as a bundle (e.g. program to improve percentage of patients with controlled blood pressure with a processes and tools for monitoring, educating, and adjusting patient medications). |
|  | *More than one component* | *2* | The intervention includes more than one component (e.g., patient brochure, new walk-in patient workflow). These components may be integrated into a package. |
|  | *One component* | *1* | The intervention includes one component only (e.g., patient letter with specialty clinic contact information). |
| 2. Behavior or actions of intervention recipients or participants to which the intervention is directed | *Multi-target* | *3* | Intervention directed at three or more behaviors or actions (e.g., educate patients, change nurse and clerk workflows, create formal communication channels between nursing and clerks). |
|  | *Dual target* | *2* | Intervention directed at two behaviors or actions (e.g., change in nurse scrubbing practices to identify patients likely to no-show, designated staff call patients on list). |
|  | *Single target* | *1* | Intervention directed at one behavior or action only (e.g. patient behavior to contact relevant clinics and services to meet their needs). |
| 3. Organizational levels and categories targeted by the intervention | *Multi-level* | *3* | Intervention directed at two or more levels (e.g., clinic [hours of operation], and categories of individuals [nurses and clerks, and patients]) |
|  | *Multi-category* | *2* | Intervention directed at two or more categories of individuals within the individual level (e.g. nurses and patients). |
|  | *Single category* | *1* | Intervention directed only at single category of individuals within the individual level (e.g. professionals or patients or policy makers). |
| 4. The degree of tailoring intended or flexibility permitted across sites or individuals in applying or implementing the intervention | *Highly tailored/flexible* | *3* | High degree of variation in implementation from teams within the clinic permitted and/or intervention designed to tailor to individuals or specific implementation settings (e.g. teams allowed to adapt and deliver patient education materials as they would like). |
|  | *Moderately tailored/flexible* | *2* | Some variation in implementation from team to team within the clinic permitted (i.e. some components of the intervention are tailored/flexible while others are not) (e.g. teams determine who is tasked with calling patients at risk of no-showing). |
|  | *Inflexible* | *1* | Intervention implementation highly standardized with minimal variation from team to team within the clinic (e.g. new workflows and scripted patient education intended to standardize clinic practice). |
| 5. The level of skill required by those delivering the intervention in order to meet the intervention objectives | *High level skills* | *3* | Extensive specialized skills required, i.e. new skills in addition to expected existing skills AND/OR the extension of existing skills to a highly specialized area AND/OR skills requiring extensive additional training. (e.g. providers perform new evidence-based clinical intervention) NOTE: not observed |
|  | *Intermediate level skills* | *2* | Some specialized skills required, i.e. a small extension to the expected existing skills of professionals, decision makers or consumers. (e.g. nurses trained in motivational interviewing) |
|  | *Basic skills* | *1* | No specialized skills required (e.g., handing out a brochure to patients). |
| 6. The level of skill required for the targeted behavior by those receiving the intervention in order to meet the intervention objectives | *High level skills* | *3* | Extensive specialized skills required. |
|  | *Intermediate level skills* | *2* | Some specialized skills required (e.g. patients or caregivers (self-)administer an infusion drug). |
|  | *Basic skills* | *1* | No specialized skills required (e.g. patients use brochure to call pharmacy when need a prescription refilled). |

1. Lewin S, Hendry M, Chandler J, Oxman AD, Michie S, Shepperd S, et al. Assessing the complexity of interventions within systematic reviews: development, content and use of a new tool (iCAT_SR). BMC Med Res Methodol. 2017 Dec;17(1):76.
