# Supplementary material for: Toolkit and distance coaching strategies: a mixed methods evaluation of a trial to implement care coordination quality improvement projects in primary care
Source: BMC Health Serv Res. 2021 Aug 14;21:817. doi: 10.1186/s12913-021-06850-1 (PMC8364700; doi:10.1186/s12913-021-06850-1)
Supplement: Supplementary file 5 — Additional file 5. Site project and outcomes table. Summary table of each CTAC site’s project, use of CTAC tools, project complexity rating, and reported project outcomes at 12 months, and implementation outcomes at 12 and 18 months. [file 12913_2021_6850_MOESM5_ESM.docx]

**Additional File 5. Site project and outcomes table**

| Site Number | System | Coached (Y/N) | Project Description | Used a CTAC Tool (Y/N) | Project Complexity Assessment (possible score range 0-18) | Project Outcome(s) at 12 months* | Implementation Outcomes at 12 months | Implementation Outcomes at 18 months |
| --- | --- | --- | --- | --- | --- | --- | --- | --- |
| 1 | A | Y | Improve the process for walk-in patients to address multiple patient needs:  (1) medication refill and renewal (tailored patient education brochure**, new nurse and scripted clerk workflows)  (2) release of information (patient education slides, workflow)  (3) new symptoms (triage nurse)  (4) lab orders (clerk and nurse workflows, patient education) | Y | 11 | Decrease in number of unscheduled nursing visits | (1) Workflows and brochure adopted and +/- consistently used across the clinic (304 patients received it over a 10-week period); brochure spread to another clinic and pharmacy  (2) Workflow about to be implemented, slides not yet available  (3) Triage nurse piloted for 2 weeks but not adopted  (4) Developed and distributed labs workflow map for clerks, multiple aspects of this project pending at 12 months | Staff turnover and lack of oversight for project components contribute to inconsistent ongoing use, but all components sustained to some degree.  New initiatives have taken focus, CTAC projects not a focus for any ongoing work.  Adaptations: Patient brochure integrated into new patient orientation packet.  Have used some of the brochure, lab value, and Release of Information & applied to chronic disease management (hypertension, diabetes) |
| 2 | A | Y | Develop a clinic brochure for patients** to improve patient-staff relationships, and improve clinic workflows for handling walk-in patients with certain needs (including standardizing patient education** about how to handle those needs in the future). | Y | 11 | Almost all of a sample of Veterans who received the brochure liked it. | Brochures and workflows adopted, +/- consistently used across the clinic. Pre-implementation recorded 14 walk-in patients during a 19 day period versus 5 walk-in patients during a seven day period post-implementation.  Spread: Several other clinics have asked for brochure. | No ongoing monitoring of brochure use, general awareness of needed updates to the brochure but no system in place to get needed feedback for or to make those updates |
| 3 | A | N | Implement Save a Trip Form** to support patient self-management | Y | 7 | None reported/tracked | Teams given flexibility in whether and how form adopted; at 12 months, at least one person from each PACT team using it in some capacity | Most teams still giving form to walk-in patients, but some also giving to new patients. |
| 4 | A | N | Initial project idea: Improve Veterans’ perceptions of clinic access | N | 0 | Nothing implemented | Nothing implemented | Nothing implemented |
| 5 | B | Y | Improve the medication reconciliation process during patient clinic visits:  (1) Customize medication lists in the EHR (2) Pre-appointment calls and letters to patients to remind them what they need to bring to their visit  (3) Clinic medication reconciliation workflows for clerks and nurses (e.g., instructing patients how to mark up their med lists)  (4) Clinic medication brochure** | Y | 12 | Reduction in volume of medication refill and renewal phone calls | (1) Changing the medication list was not feasible  (2), (3) and (4) Reminder calls and letters, workflow, and brochure integrated into processes around clinic visits. During a seven week period, 60 brochure were distributed to patients. | Brochure still being used in all teams; certain people are responsible for monitoring brochure needs and making copies. Unclear whether medication lists are still being used by providers or if reminder letters are still being sent.  Spread: Brochure spread to at least 2 other clinics; two of the project leads continue to work through formal primary care channels for its spread throughout local primary care (e.g., in new patient orientation kit) and pharmacy service. |
| 6 | B | N | Reduce the number of specialty consult discontinuations by giving patients a form letter with consult clinic phone number | N | 8 | During test period, decrease in number of consult discontinuations | Piloted form with one provider for one month | At 18 months, recent decision to implement form in the clinic |
| 7 | C | Y | (1) Educate pre-diabetic patients about diabetes prevention and enroll them in a Healthy Living class (staff education, patient mailers).  (2) Adapt the process developed for pre-diabetic patients for patients with A1C >9 and no A1C test in the past 12 months. | N | 12 | Increased number of referrals to Healthy Living class; during a four month period, 29% of enrollees heard about the class from the QI project outreach mailer.  Post-class improvements in A1C scores and percentage with knowledge deficits. | (1) Process of identifying, educating, and referring pre-diabetic patients adopted by all clinic teams, though not always consistently conducted. Planned spread throughout broader clinic.  (2) Mailings adopted as part of intensive case management of high-risk patients (A1C >9). Some teams adopted letter for use with patients with A1C >9 and no A1C test in the past 12 months. | Decreased referrals to Healthy Living class, unclear how many teams still using the process.  Spread: No spread yet, but planned presentation with leadership about project soon after 18-month interview was thought to have the potential to initiate spread. |
| 8 | C | N | Reduce the number of unscheduled appointments using Save a Trip Form** and standardize key talking points for nurses using form (including promoting use of secure messaging). | Y | 7 | No significant change in percentage of unscheduled nursing visits.  Increased volume of secure messaging (saw a similar trend in 2 clinics not involved in project), but the number of people signing up for secure messaging was unchanged. | Not all teams fully implementing the form but used by the 10 teams in the clinic. Spread: Form adopted by people in at least two other clinics. | At 18 months, in use with 23 teams.  Adaptations: In preparation of new VA policy on community care, updated form to include more information and feedback gathered from patients and staff.  Spread: Leadership wants to spread across local primary care clinics. |
| 9 | D | Y | Improve the process for managing unscheduled patient visits to the clinic and help prevent unnecessary walk-in visits through patient education brochure**, improved workflows, and formalized nurse-clerk communication. | Y | 11 | Patients and staff satisfied with new brochure, and staff generally happy with new workflows and perceived improved communication.  Reduced volume of walk-ins for non-medical issues. | Most staff have adopted the brochures and workflows. Several other clinics have expressed interest in brochure or workflows.  Clinic nursing and medical support staff management instituted regular meetings. | Brochures are still being distributed and the workflow implemented.  Spread: The brochure was shared with other clinics and some nurses, and sounds like it is being used in adapted forms elsewhere in local system. |
| 10 | D | N | Decrease missed opportunity rate using the Daily Missed Opportunity Rate report to call patients with a 20% or more chance of not showing up for appointments. | N | 12 | No show rate decreased | Customized daily missed opportunities report made available to all teams, but teams decide whether and how to use it. | No show rate is about the same as at 12 months. Processes still being implemented. Clinic daily missed opportunity report monitored as part of other clinic monitoring activities. |
| 11 | E | Y | (1) Improve the process for managing unscheduled patient visits to the clinic (new walk-in tracking and triage form, nurse and clerk workflows) and help prevent unnecessary walk-in visits through patient education (clinic brochure**, script for brochure).  (2) Extended clinic hours to better meet needs and desires of patients. | Y | 12 | A sample of Veterans who received the brochure provided mostly positive feedback. | (1) New processes and patient education implemented clinic wide. Brochure incorporated into New Patient Orientation. Portions planned to be implemented in another clinic.  (2) Not implemented. | (1) Triage form, workflows, and brochures sustained use. Co-champions responsible for making updates to materials as needed, and one person responsible for making brochure copies.  Spread: Triage form adopted by another clinic.  (2) Extended clinic hours still not approved by system leadership due to, in part, concerns about clinic staffing adequacy but is still seen as a possibility. |
| 12 | E | N | Improve the percentage of patients with controlled blood pressure through follow-up with semimonthly nursing visits for blood pressure monitoring and patient education (including a medication tracker**), and as needed medication management from provider. | Y | 11 | No systematic data reported. | Piloted with one provider.  At 12 months, had recently been implemented clinic-wide with pharmacist providing follow-up and patients encouraged to attend new hypertension class; unclear the degree to which teams utilizing this process. | No information available. |

*As measured and reported by the sites. Sites used different measures and sampling frames, and some noted that they did not know for sure whether trends in tracked metrics reflected true project impacts. Coached sites often did much data collection related to the development of their tools and processes (e.g., brochure usability testing, feedback from staff on proposed workflow changes) but did less to measure impacts of implementation of their interventions.

**Utilized a CTAC toolkit
