# Supplementary material for: Toolkit and distance coaching strategies: a mixed methods evaluation of a trial to implement care coordination quality improvement projects in primary care
Source: BMC Health Serv Res. 2021 Aug 14;21:817. doi: 10.1186/s12913-021-06850-1 (PMC8364700; doi:10.1186/s12913-021-06850-1)
Supplement: Supplementary file 6 — Additional file 6. Barriers and Facilitators to Project Implementation. Detailed description of the barriers and facilitators sites encountered when implementing their QI projects. [file 12913_2021_6850_MOESM6_ESM.docx]

**Additional File 6:** **Barriers and Facilitators to Project Implementation**

Several factors were important influences across sites (see Figure 6.1). Clinic staffing pressures, champion and staff competing priorities, and practice silos represented barriers at many sites. For coached sites planning introduction of tools involving the electronic health record, inflexible technology or lack of access to resources to help them utilize the technologies were major barriers. Having an adept champion, who was able to engage other staff and help problem solve challenges, and interdisciplinary collaboration on the CTAC project appear to support successful implementation.

Figure 6.1. Barriers and facilitators to site implementation

**
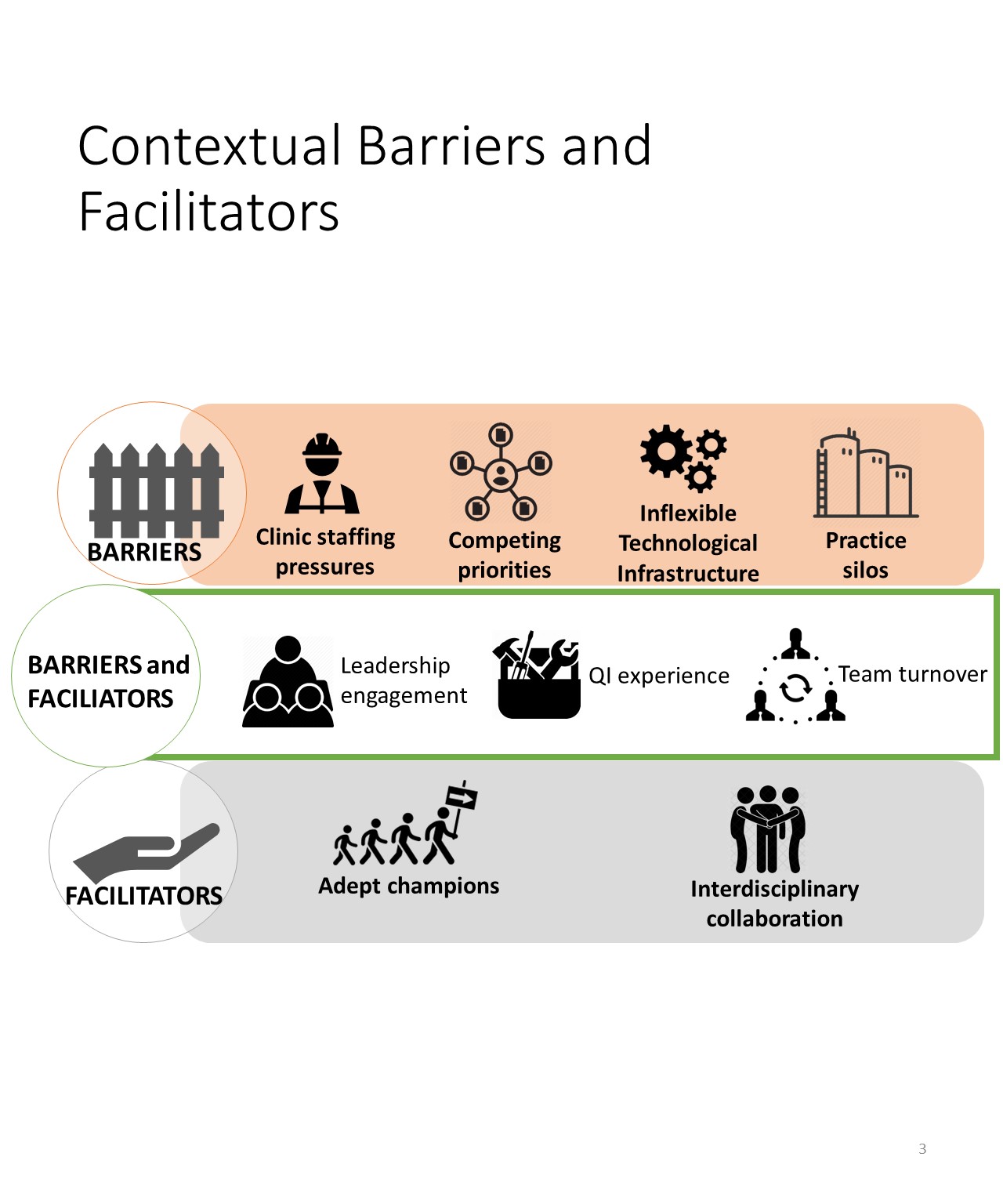
**

Several factors could support or hinder implementation depending on the circumstances. Clinic leadership engagement with the local QI team was often helpful: leaders brought insight about organizational priorities and could frame projects within a larger organizational context, which brought meaning and purpose, and leaders could also provide needed resources (e.g., protected time). However, leaders often had spotty engagement: their presence could disrupt project momentum if they made suggestions or questioned decisions that altered established plans. QI experience was variable among champions and teams. For coached and non-coached sites, champions with knowledge and skills could help shepherd a project forward. But for coached sites, when that QI experience differed from the approaches the CTAC coach drew upon, that difference could create conflict and make it difficult for the coach to gain buy-in, especially for data collection during times of staffing shortage. QI team turnover could also have variable impacts on implementation. Losing team members could result in a loss of momentum; however, for teams that were struggling, new members could bring new energy and ideas, as well as could shift team dynamics in positive ways which could help move projects forward.
